# First report of computational protein–ligand docking to evaluate susceptibility to HIV integrase inhibitors in HIV-infected Iranian patients

**DOI:** 10.1016/j.bbrep.2022.101254

**Published:** 2022-03-29

**Authors:** Farzane Ghasabi, Ava Hashempour, Nastaran Khodadad, Soudabeh Bemani, Parisa Keshani, Mohamad Javad Shekiba, Zahra Hasanshahi

**Affiliations:** Shiraz HIV/AIDS Research Center, Institute of Health, Shiraz University of Medical Sciences, Shiraz, Iran

**Keywords:** HIV, Integrase inhibitors, CRF35-AD, Drug resistance, Integrase, Molecular docking, INT, Integrase, INTIs, Integrase inhibitors (INTIs), Resistance-associated mutations, RAMs, Naturally occurring polymorphisms, NOPs, Post-translational modification, PTM, Human immunodeficiency virus, HIV, Antiretroviral therapy, ART, N-terminal domain, NTD, Catalytic core domain, CCD, C-terminal domain, CTD, Raltegravir, RAL, Elvitegravir, EVG, Dolutegravir, DTG, Bictegravir, BIC, Cabotegravir, CBT, Grand average hydropathy, GRAVY, Injecting drug users, IDUs, Behavioral Diseases Consultation Center, BDCC

## Abstract

**Background:**

Iran has recently included integrase (INT) inhibitors (INTIs) in the first‐line treatment regimen in human immunodeficiency virus (HIV)-infected patients. However, there is no bioinformatics data to elaborate the impact of resistance-associated mutations (RAMs) and naturally occurring polymorphisms (NOPs) on INTIs treatment outcome in Iranian patients.

**Method:**

In this cross-sectional survey, 850 HIV-1-infected patients enrolled; of them, 78 samples had successful sequencing results for INT gene. Several analyses were performed including docking screening, genotypic resistance, secondary/tertiary structures, post-translational modification (PTM), immune epitopes, etc.

**Result:**

The average docking energy (E value) of different samples with elvitegravir (EVG) and raltegravir (RAL) was more than other INTIs. Phylogenetic tree analysis and Stanford HIV Subtyping program revealed HIV-1 CRF35-AD was the predominant subtype (94.9%) in our cases; in any event, online subtyping tools confirmed A1 as the most frequent subtype. For the first time, CRF-01B and BF were identified as new subtypes in Iran. Decreased CD4 count was associated with several factors: poor or unstable adherence, naïve treatment, and drug user status.

**Conclusion:**

As the first bioinformatic report on HIV-integrase from Iran, this study indicates that EVG and RAL are the optimal INTIs in first-line antiretroviral therapy (ART) in Iranian patients. Some conserved motifs and specific amino acids in INT-protein binding sites have characterized that mutation(s) in them may disrupt INT-drugs interaction and cause a significant loss in susceptibility to INTIs. Good adherence, treatment of naïve patients, and monitoring injection drug users are fundamental factors to control HIV infection in Iran effectively.

## Highlights

• Integrase inhibitors (INTIs) are the first‐line treatment in HIV-infected patients.
• The average docking energy in elvitegravir and raltegravir was more than other INTIs.
• Phylogenic tree analysis revealed HIV-1 CRF35-AD was the predominant subtype.
• For the first time, CRF-01B and BF were identified as new subtypes in Iran.

## 1 Introduction

Deadly disease outbreaks and emerging viral diseases inflict severe public health in the developed and developing countries [1] Among these, AIDS continues to be a significant problem worldwide. HIV-1 has three essential enzyme uses for its replication; integrase (INT) is one of them that catalyzes viral integration. HIV-INT consists of three structural and functional domains: the N-terminal domain (NTD, residues 1–49), the catalytic core domain (CCD, residues 50–212), and the C-terminal domain (CTD, residues 213–288). It also contains a conserved DDE motif encompassing amino acids Asp64, Asp116, and Glu152 in the CCD necessary for drug binding and enzyme activity [2]. As a result of the drug resistance development across currently available drugs, WHO has put forth the use of INTIs: raltegravir (RAL) and elvitegravir (EVG) as the first-generation inhibitors, dolutegravir (DTG) and bictegravir (BIC), along with the late-phase clinically trialed cabotegravir (CAB), as the second-generation INTIs [3]. First-generation INTIs have a relatively low genetic barrier to resistance, whereas second-generation INTIs confer to a higher genetic barrier against RAMs [2]. Treatment failure occurs due to HIV mutations, poor adherence, variations in pharmacokinetics [4], etc. Two categories of mutations are related to INTIs drug resistance: RAMs and NOPs as the primary and secondary pathways, respectively. NOPs are subtype-specific polymorphic mutations that affect INT DNA binding affinity in the presence of RAMs [5]. Little is known about the potency of mutations influencing susceptibility to INTIs in CRF35-AD subtype virus treatment.

In this study, for the first time computational methods and molecular analysis were done to assess the influence of RAMs and NOPs on docking energy between INTIs and INT protein complexes in Iranian patients.

Since INTIs are currently advised in Iran when patients do not respond to first- and second-line ART, more information on the drug susceptibility, primary, and secondary drug resistance mutations profile of INTIs is required to guide its implementation across the country.

Moreover, posttranslational modifications analysis was performed on patient's INT sequences.

## 2 Method

### 2.1 Study population

This cross-sectional study was conducted from June 2017 to June 2020, before the initiation of Iran's national HIV treatment program and the introduction of INTIs. Plasma samples were collected for viral load assay from 850 HIV-infected patients originating from the south of Iran; they were either antiretroviral therapy-naïve or RTIs and/or PIs treatment-experienced patients with a viral load above 1000 IU/ml enrolling at the Behavioral Diseases Consultation Center (BDCC) affiliated with Shiraz University of Medical Sciences. Medication adherence means a ratio of the number of pills doses taken in patients to the number of doses prescribed over a given period [6]. According to the self-report inventory, the patients' adherence level were classified into good, unstable, or poor adherence groups. Patients with an excellent history of adherence and intermittent phases of non-adherence were placed in good and unstable adherence groups, respectively. The poor adherence category was related to patients who could rarely adhere to ARTs [7]. In this report, poor and unstable are defined as reduced adherence.

### 2.2 CD4 count and biochemical tests

Using “FACSPresto Near-Patient CD4 Counter (BD Biosciences)”, CD4 T-cell count was performed. Aspartate aminotransferase (AST) and Alanine aminotransferase (ALT) levels (IU/L) in the serum samples were measured using the commercial enzymatic kits of Biorex-Fars Company (Shiraz, Iran) and DIRUI Automatic Biochemistry Machine.

### 2.3 HIV viral load, RT-nested-PCR, and sequencing

Serum RNA was extracted by the QIAamp Viral RNA Mini Extraction Kit (Qiagen, Germany), and Artus HI Virus-1 RT-PCR kit (Qiagen) was utilized to define viral load in all samples. HIV-INT region was amplified by RT-nested PCR using the primers listed in Table 1. Finally, the positive amplicons were purified by a gel extraction kit (QiagenGmbH, Hilden, Germany) followed by Sanger sequencing of both DNA strands with the limit of detection ∼15–20%. (Niagene Noor Company, Iran).

**Table 1:** List of primers used in this study and thermal-cycling conditions of nested-PCR.

Primers	Sequence	Location (AB703607)	Products length	PCR (I) and (II) Programs
Outer Forward	TGGAGGAGGAGATATGAGGG	6832–6851	733	94 (2 min) 35 cycles 94 (30 s) 40 (30 s) 72 (60 s) 72 (5 min)
Outer Reverse	AAGGTGAGTATCCCTGCCTAAC	7544–7565		
Inner Forward	TTCATTGGGTTCTTAGGAGCAG	6977–6998	527	
Inner Reverse	ATCCTATTAAGCCTCCTACTATC	7483–7505		

### 2.4 Drug resistance analyses

Based on the RAMs and the high frequent NOPs detected in the INT sequences, 78 patients were clustered into 11 groups (1-11) and 7 models including five INTIs mutated models (RAL, EVG, CAB, DTG, and BIC mutated models) and two mutants: mutant 1 and mutant 2. The high frequent NOPs in the INT protein is shown in Table 2.

**Table 2:** High Frequent NOPs in the INT protein.

List of NOPs	Percentage
N39S	29.5
V72I	2.6
V112I	33.3
G134S	35.9
M203I	61.5
Q216H	24.4
V249I	1.3

In this study, INTs of all 11 groups and 7 models are called mutated INT genes/proteins.

Each group encompasses distinct mutations (Table 3); also, five INTIs mutated models and mutant 1 and 2 were generated by substitution of the exact mutation(s) (Table 4) in the INT reference gene (GenBank accession number: AB703607). For example, to build CAB mutated model, all RAMs presented in our integrase genes that were attributed to the failure of CAB were inserted in INT reference gene. In addition, mutant 1 and mutant 2 were built by the insertion of all RAMs and high prevalent NOPs in the INT reference sequence. Nucleotide and amino acid sequences of mutated INTs genes/proteins are shown in Supplemental Table 1.

**Table 3:** Characteristic of mutated INT genes/proteins in groups 1–11.

Groups	RAMs and/or high frequent NOPs in patient's genes
Ref. (AB703607)	NA (not applicable)
Group 1	The four most frequent NOPs [N39 S^d^ (29.5%), V112I^d^ (33.3%), G134S^d^ (35.9%), M203I^d^ (61.5%)]
Group 2	The three most frequent NOPs [N39 S^d^ (29.5%), V112I^d^ (33.3%), M203I^d^ (61.5%)]
Group 3	The three most frequent NOPs [V112I^d^ (33.3%), G134S^d^ (35.9%), M203I^d^ (61.5%)]
Group 4	The three most frequent NOPs [G134S^d^ (35.9%), M203I^d^ (61.5%), Q216H^d^ (24.4%)]
Group 5	Major RAM [R263K^b^ (1.3%)]
Group 6	Accessory RAM [L74 M^a^ (6.4%)], The most frequent NOP [V112T^d^ (33.3%)]
Group 7	Accessory RAM [S230 N^a^ (6.4%)]
Group 8	Minor RAM [L74I^c^ (5.1%)]
Group 9	Accessory RAM [L74 M^a^ (6.4%)]
Group 10	Accessory RAM [Q95K^a^ (3.8%)]
Group 11	Accessory RAM [G163R^a^ (1.3%)]

a Accessory RAM.

b Major RAM.

c Minor RAM.

d The most frequent NOP.

**Table 4:** Characteristic of mutated INT genes/proteins in 7 models.

Models	Positions of amino acids inserted in reference gene
BIC mutated model (All RAMs associated with BIC failure treatment)	M50I^a^ (1.3%), V72I^d^ (2.6%), V249I^d^ (1.3%), R263K^b^ (1.3%)
DTG mutated models (All RAMs associated with DTG failure treatment)	M50I^a^ (1.3%), V72I^d^ (2.6%), S230 N^a^ (6.4%), V249I^d^ (1.3%), R263K^b^ (1.3%)
CAB mutated models (All RAMs associated with CAB failure treatment)	M50I^a^ (1.3%), L74 M^a^ (6.4%), G163R^a^ (1.3%), R263K^b^ (1.3%)
BIC mutated models (All RAMs associated with EVG failure treatment)	Q95K^a^ (3.8%), R263K^b^ (1.3%)
RAL mutated models (All RAMs associated with RAL failure treatment)	L74 M^a^ (6.4%), G163R^a^ (1.3%), S230 N^a^ (6.4%)
Mutant 1 (All RAMs and the most frequent NOPs)	N39S^d^ (29.5%), V112I^d^ (33.3%), G134S^d^ (35.9%), M203I^d^ (61.5%), Q216H^d^ (24.4%), R263K^b^ (1.3%), L74 M^a^ (6.4%), S230 N^a^ (6.4%), Q95K^a^ (3.8%), G163R^a^ (1.3%), M50I^a^ (1.3%), V72I^d^ (2.6%), V249I^d^ (1.3%)
Mutant 2 (All RAMs and the most frequent NOPs)	N39S^d^ (29.5%), V112T^d^ (33.3%), G134S^d^ (35.9%), M203I^d^ (61.5%), Q216H^d^ (24.4%), R263K^b^ (1.3%), L74I^c^ (5.1%), S230 N^a^ (6.4%), Q95K^a^ (3.8%), G163R^a^ (1.3%), M50I^a^ (1.3%), V72I^d^ (2.6%), V249T^d^ (1.3%)

a Accessory RAM.

b Major RAM.

c Minor RAM.

d The most frequent NOP.

### 2.5 ProtParam; physico-chemical properties

“Expasy's ProtParam” [8] (Table 5a) was employed to estimate various INT protein properties including extinction coefficient, theoretical isoelectric point (pI), molecular weight, instability index, grand average hydropathy (GRAVY), and aliphatic index [[9], [10], [11]].

**Table 5:** List of links used in this study.

Table	Software	URL	Function
**5a**	ProtParam	http://expasy.org/tools/protparam.html	Physico-chemical properties
**5b**	GPS 5.0 kinase	http://gps.biocuckoo.cn/wsresult.php	Phosphorylation sites prediction
	PhosphoSVM	http://sysbio.unl.edu/PhosphoSVM/prediction.php	
	Phos3D	http://phos3d.mpimp-golm.mpg.de/	
	NetPhos 3.1	http://www.cbs.dtu.dk/services/NetPhos/	
	SCRATCH	http://scratch.proteomics.ics.uci.edu/	Disulfide bands prediction
	DIANNA	http://clavius.bc.edu/∼clotelab/DiANNA	
	VADAR	http://vadar.wishartlab.com/	
	DbD2	http://cptweb.cpt.wayne.edu/DbD2/index.php	
	PIC	http://pic.mbu.iisc.ernet.in/job.html	
	NetOGlyc 4.0	http://www.cbs.dtu.dk/services/NetOGlyc/	Glycosylation sites prediction
	GlycoMine	https://glycomine.erc.monash.edu/Lab/GlycoMine	
	GPP	https://comp.chem.nottingham.ac.uk/cgi-bin/glyco/bin/getparams.cgi	
	NetCGlyc 1.0	http://www.cbs.dtu.dk/services/NetCGlyc	
	NetNGlyc 1.0	http://www.cbs.dtu.dk/services/NetNGlyc/	
	JASSA	http://www.jassa.fr/	SUMOylation sites prediction
	SUMOgo,	http://predictor.nchu.edu.tw/SUMOgo/	
	SUMOplot,	https://www.abcepta.com/sumoplot	
	GPS-SUMO	http://sumosp.biocuckoo.org/	
	RUBI	http://old.protein.bio.unipd.it/rubi/	Ubiquitylation sites prediction
**5c**	SOPMA	https://npsa-prabi.ibcp.fr/NPSA/npsa_sopma.html	Secondary structure prediction
**5d**	I-TASSER	https://zhanglab.ccmb.med.umich.edu/I-TASSER/	Tertiary structure prediction
	GalaxyRefine	http://galaxy.seoklab.org/cgi-bin/submit.cgi?type=REFINE	Protein refinement
	ProSA-web	https://prosa.services.came.sbg.ac.at/prosa.php	Protein model validation
	RAMPAGE	http://www.ebi.ac.uk/thornton-srv/databases/pdbsum/Generate.html	
	ERRAT	https://servicesn.mbi.ucla.edu/ERRAT/	
	Qmean	https://swissmodel.expasy.org/qmean/	
**5e**	BcePred	http://ailab-projects1.ist.psu.edu:8080/bcpred/predict.html	Linear B cell epitopes
	Bepipred	http://www.cbs.dtu.dk/services/BepiPred/	
	Ellipro	http://tools.iedb.org/ellipro/	Discontinuous B cell epitopes
	VaxiJen	http://www.ddg-pharmfac.net/vaxijen/VaxiJen/VaxiJen.html	Prediction of the most probable protective antigens
**5f**	Stanford HIV Subtyping program	https://hivdb.stanford.edu/page/hiv-subtyper/	HIV subtyping
	REGA HIV-1 Subtyping	https://www.genomedetective.com/app/typingtool/hiv	
	NCBI Genotyping	https://www.ncbi.nlm.nih.gov/projects/genotyping/formpage.cgi	
	Geno2pheno	https://integrase.geno2pheno.org/	
	HIV-GRADE	http://www.hiv-grade.de/grade/deployed/grade.pl?program=hivalg	
	COMET	https://comet.lih.lu/	
	jpHMM	http://jphmm.gobics.de/submission_hiv.html	

### 2.6 Post-modification changes

PTMs of proteins are involved in the attachment of functional groups or small proteins such as disulfide bridging, phosphorylation, glycosylation, etc. to specific residue in the protein that alters the charge and the structure of the protein. NetPhos [11,12], DISPHOS [13,14], and NetPhosK [15,16] were performed to assess the kinas specific phosphorylation sites in INT proteins, and SCRATCH [13], DIANNA [14], VADAR [17], DbD2 [18], and PIC server [19] were used to define the disulfide bonds. Disulfide post-modification analyses showed that mutations might impact the number of bonds and bond patterns. Formation of the disulfide bond locks the structure in place and mostly increases the stability and half-life of the proteins [20]. Glycosylation is one of the most widespread and versatile protein modifications required for progeny formation and proper folding of viral proteins. Glycosylation sites were predicted via GPP Prediction Server [21], NetOGlyc [22] and GlycoMine [23]; moreover, SUMOylation sites were characterized by JASSA [24], SUMOgo [25], SUMOplot [26], and GPS-SUMO [27]. Finally, Ubiquitylation site was suggested using RUBI tools [28] (Table 5b).

### 2.7 SOPMA: secondary structure

Secondary structural features can change the binding pocket properties or affect the stability of the whole protein [29] that influence the accessibility of drugs to a protein's active site. This structure was interpreted using “SOPMA” software [13,30,31] (Table 5c). Four conformational states were suggested in all mutated INT proteins and reference, including Helix, Sheet, turn, and coil with the window width and similarity threshold of 17 and 8, respectively.

### 2.8 Tertiary structures and model evaluation

To determine the tertiary structure, amino acid sequences of the mutated INT proteins were submitted in “I-TASSER” [32,33]. Among the various models suggested by I-TASSER, the highest C-score model was selected as the best one and went through the refinement process in “GalaxyRefine” [16,34]. Finally, all suggested 3D structures were evaluated for the reliability, quality, and stereochemistry by “ProSA-web” [35], ‘RAMPAGE” [36], “ERRAT” [37], and “Qmean” [8,10] (Table 5d).

### 2.9 Ligand receptor docking and visualization

The higher docking score represented better binding affinity, indicating the strong attachment of integrase inhibitors to integrase proteins to suppress HIV functions that would contribute to the promising treatment outcome. The impact of RAMs and high frequent NOPs on INTIs treatment outcome was evaluated through docking analysis of INTs with BIC, EVG, DTG, RAL, and CAB drugs. For this, the PDB format of five INTIs was obtained from the DrugBank database (https://go.drugbank.com/) [38]. To find the possible interaction between mutated INT proteins and INTIs, “Hex” software [39] was employed for docking, and visualization of the amino acids interacting with INTIs was assessed by Discovery Studio software [40].

### 2.10 B-cell epitopes prediction

To assess B-cell linear epitope, a consensus sequence generated by the alignment of all 78 samples was summited in “BcePred” [41], “Bepipred” [42], and Ellipro [43] online programs. The most probable potential antigens were selected via VaxiJen” software [8] (Table 5e).

### 2.11 HIV-1 subtype classification and phylogenetic analysis

Phylogenetic tree and seven online subtyping software (REGA HIV-1 subtyping [44], Stanford HIV subtyping program [45], NCBI genotyping [46], Geno2pheno INT [47], HIV-GRADE [48], COMET [49], and jpHMM [50]) were applied to declare the subtype of all 78 patients (Table 5f). Phylogenic analysis was performed on the sample sequences along with 89 reference strains from various subtypes sequences (Supplemental Table 2), using MEGA software [51]. The neighbor-joining method constructed the phylogenetic tree based on the Kimura 2‐parameter distance matrix listed in the MEGA software. Additionally, the statistical significance of the phylogenetic tree was evaluated by the bootstrap method (1000 replicates).

### 2.12 Statistical analysis

As the data was nonparametric, median (Q1 and 3) and frequency (%) were used for quantitative and quantitative data analysis and Kruskal‐Wallis and Mann‐Whitney tests were used to analyze the data. To interpret the relationship between quantitative variables, we employed Spearman coefficient correlation and for quantitative variables, chi-squared test was used. Differences with a P-value of 0.05 were regarded as statistically significant. Data management was performed using SPSS version 20.0 [52].

## 3 Result

### 3.1 Demographic characteristics, CD4 count, and HIV viral load

Of 850 patients for whom HIV viral load test was performed, only 151 individuals had a viral load exceeding 1000 copies/ml that were considered for RT-nested-PCR during the study period (June 2017–June 2020). This period was before the availability of INTIs in Iran, so it was chosen to provide baseline antiviral resistance data and to ensure the patients were INTIs-naive. The appropriate result in RT-nested-PCR and sequencing was achieved for 78 patients: 62 treatment patients and 16 naïve-treatment individuals. The demographic and laboratory characteristics of 78 patients are summarized in Table 6.

**Table 6:** The demographic data of patients and clinical characteristics.

Parameters	Frequency/Median		
Gender: female/male	26/52 (33/67%)		
Age, median (Min-Max)	39 (3–57)	≤45	29 (37%)
		44≤age≤35	49 (63%)
Years of HIV diagnosis, median (Min-Max)	8.5 (1–17)	9≥	40 (51%)
		10≤	37(47%)
WHO clinical stage	Stage 1	30 (38%)	
	Stage 2	30 (38%)	
	Stage 3	20 (26%)	
	Stage 4	3 (4%)	
Rout of HIV Transmission	a IDU		39 (50%)
	Sexual		24 (34.6%)
	Mother-to-child transmission		7 (9%)
	Unknown		5 (6.4%)
Treatment status	Treated with RTIs and/or PIs		62
	Naïve		16
Symptom y/n (%)	52/26 (67/33%)		
	Gastrointestinal symptoms(%)		42 (54%)
	Respiratory symptoms (%)		7 (9%)
	Neurologic symptoms (%)		13 (16.7%)
	Skin symptoms (%)		5 (6.4%)
HBsAg positive	1 (1.3%)		
HCVAb positive	37 (47%)		
CD4 cell count (cells/mm2), median (Min-Max)	250 (8–1427)	<200	35 (45%)
		200 ≤ CD4 <500	34 (44%)
		≤500	9 (11%)
HIV viral load (log10copies/mL), median (Min-Max)	214301	(3707-12 × 106)	
		1000–9999	5 (6%)
		10000–99999	19 (24%)
		≥100000	54 (70%)
AST (IU/L), median (Min-Max)	32.5 (11–200)		
ALT (IU/L), median (Min-Max)	26 (5–172)		
Integrase mutation, median (Min-Max)	6 (1–17)	b NOPs	61 (78%)
		NOPs + c RAMs	17 (22)
Adherence category	Good	16%	
	Unstable	44%	
	Poor	40%	
Susceptibility to INTIs (resistance/susceptible) (%)	5/73 (6.4/93.6%)		

a : Intravenous drug use.

b : Natural occurring mutations.

c : Resistant associated mutations.

**Fig. 1:**
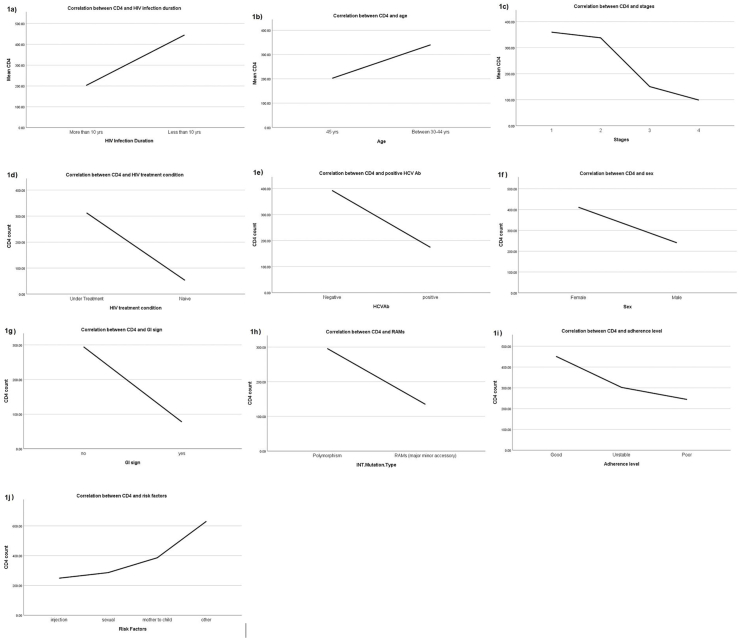
Correlation between lower CD4 counts and more prolonged HIV infection (1a), older age (1b), later stage of HIV infection (1c), naïve treatment patients (1d), seroconversion to anti-HCV antibody positive status (1e), male gender (1f), the symptom of gastrointestinal diseases (1g), the emergence of RAMs (1h), reduced adherence to previous ART regime(s) (1i), and being injection drug users (IDUs) (1j).

**Fig. 2:**
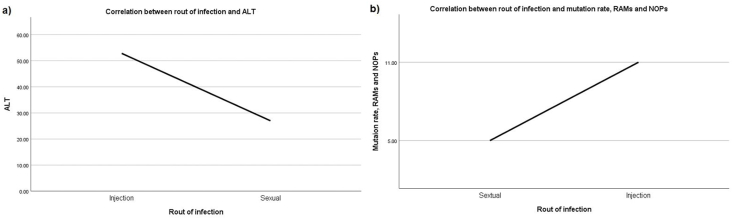
Correlation between patients infected with HIV through sex, IDUs with higher level of ALT (a), mutation rate, RAMs, and NOPs (b).

Among treatment-experiment people, only 16% were categorized as good adherence, whereas 84% had reduced adherence. A significant correlation was observed between lower CD4 count and more prolonged HIV infection, older age, later stage of HIV infection, naïve treatment patients, seroconversion to anti-HCV antibody positive status, male gender, the symptom of gastrointestinal diseases, the emergence of RAMs, reduced adherence to previous ART regime(s), and being injection drug users (IDUs) (p < 0.05) (Fig. 1). In addition, compared with patients infected with HIV through sex, IDUs showed a significantly higher level of ALT, mutation rate, RAMs, and NOPs (p < 0.05) (Fig. 2).

The median CD4^+^ T cell count and viral load in patients with good adherence were 451 cells/mm^3^, 213000 copies/ml; in naïve and reduced adherence patients, they were 264 cells/mm^3^ and 991000 copies/ml that were significantly different. The factors associated with CD4 count decline included the presence of any symptoms, naïve treatment status, reduced adherence even to reverse transcriptase and/or protease inhibitors, IDUs status, in men gender, >45 years old, longer duration of HIV infection, and co-infection with HCV. Patients with CD4 levels of 100 or less, or more than 500 cells/mm^3^ showed a viral load of more than one million; furthermore, patients with neurological and gastrointestinal symptoms corresponded to significantly higher viral load (p < 0.05)*.*

### 3.2 Genotypic drug resistance analysis

INT nucleotide sequences and related amino acid sequences were aligned and compared with a reference sequence using CLC Main Workbench software (CLC bio, Boston, MA, USA). The presence of RAMs and NOPs was identified using the Stanford University genotypic resistance interpretation algorithm, HIVdb version 8.3 (http://hivdb.stanford.edu/).

### 3.3 RAMs and NOPs in patients and INT domains

Sequenced samples were screened for the presence of RAMs and NOPs in HIV-INT region in all patients (Table 7). The data showed that only one major (R263K) and minor (L74I) resistance mutation against INTIs were present in our samples. Accessory mutations were found in 14/78 (17.94%) patients: L74 M (5 cases, 6.41%), Q95K (3 cases, 3.8%), G163R (1 case, 1.3%), and S230 N (5 cases, 6.4%). Of which, one sequence had two accessory mutations, namely L74 M and S230 N.

**Table 7:** RAMs and NOPs founded in HIV-INT proteins.

RAMS	R263K (major), L74I (minor), S230 N, L74 M, Q95K, G163R, M50I, V72I, and V249I (accessory mutations)
NOPs	I31V, M50L, L101I/M, V112A/I/L/T, A124 N/D/T, I201 T/V, T218S, L234I/V, A265 T/V, R269S, S119 C/P, S24 A/G, I31V, L63 N/I, Q62G, C65I, H67R, L68I, K71Q, A76G, S81G, I84V, P90 A/S, 216 N/H/K, K219 N, N222K, D232E, P233 N, A239S, L241I, K244E, G134 N/S, I135 T/V, E167D, I208 L/M, D256 E/T, A91S, G94I, 96Q, L102I/V, P109T, K111 R/S, V113I, H114R/Q/G, D116V, S123 N/G, Q252S, N254 R/K, S255 E/G/T, I257V, K266T, I267 N/P, I268H, D270S, Y271 S/P, K273T, F126V, Q137H, F139Y, I141V, N144D, V150A, I162L, A169T, E170D, H171Y, K274R, M275L, G277D, E48G, K188E, R199I, R204K, L172I/P, K173R, A175P, F181L, A205S, T210A/I, K211R

Additionally, NOPs were found in all patients and the frequencies were different among participants; the most frequent NOPs were M203I (48 cases, 62.3%) followed by G134S (28 cases, 36.4%), V112I (26 cases, 33.8%), N39P (21 cases, 27.3%), and G216 (19 cases, 24.7%).

The distribution of mutations in three INT domains was different, and the catalytic or functional domain was found to harbor the maximum number of RAMs (L74 M, Q95K, and G163R) and the most frequent NOPs [M203I (61.5%), G134S (35.9%), V112I (33.3%) and N39S (29.5%)] (Table 8).

**Table 8:** Mutations distribution in three domains of INT protein.

Domain	Frequency	Mutations
NTD (1-46)	1	V37I, A38P, D41 N, C43Y, E48G, A49T
	2	S24 A/G, L45V
	4	I36Q/L/K/V
	5	I31V, V32I, E35Q/G/K
	22	N39 S/T
CCD (50–212)	1	M50L, V54I, C56T, S57V, P58R, G59Q, W61C, Q62G, L63 N/I, C65I, L68I, A76G, S81G, I84V, G94I, E96Q, P109T, D116V, T122S, Q137H, A169T, E170D, H171Y, K173R, A175P, K188E, G190K, R199I, D202 N, R204K, I141V, N144D, G149R, V150A, I162L, G163R,E212Q
	2	V72I, A91S, S123 N/G, F181L, I201 T/V, T210A/I,
	3	H67R, Q95K, L102I/V, H114R/Q/G, L172I/P, A205S, I208 L/M, K211R
	4	K71Q, P90 A/S, K111 R/S
	5	L101I/M, A124 N/D/T
	6	M60I, V113I
	7	S119 C/P, F139Y
	8	I135 T/V
	9	L74I/M, E167D
	10	F126V
	39	G134 N/S
	40	V112A/I/L/T
	48	M203I
CTD (213–288)	1	T218S, K219 N, D232E, P233 N, A239S, L241I, K244E, V249I, Q252S, I257V, R263K, K266T, I267 N/P, I268H, R269S, D270S, K273T, K274R, M275L, G277D
	2	Y271 S/P
	3	N254 R/K, D256 E/T
	5	N222K, S230 N, S255 E/G/T, A265 T/V
	9	L234I/V
	27	Q216 N/H/K

### 3.4 ExPASy ProtParam analysis

Mutated INT proteins with 227, 281 or 282 amino acids length and the average molecular weight of 31.20 KD had the theoretical pI of around 8.68. The average instability and aliphatic index were 29.62 and 84.15, respectively. Next, in vivo half-lives was estimated to be 1.1 h in mammalian cell and >2 and > 3 min in *Escherichia coli* and yeast. Grand average hydropathy (GRAVY) signified that all INT proteins had been hydropathy with a negative score of −0.359 (Table 9).

**Table 9:** The “Protparam” results of INT mutated proteins and selected reference.

	Number of amino acids	Molecular weight	Theoretical pI	Half-life in mammalian reticulocytes	Half-life in yeast	Half-life in *Escherichia coli*	Instability index	Aliphatic index	GRAVY
Ref. (AB703607)	277	31083.71	8.68	1.1 h	>3 min	>2 min	Stable, 31.11	83.47	−0.355
Group 1	277	31008.63	8.68	1.1 h	>3 min	>2 min	Stable, 32.58	85.60	−0.325
Group 2	277	31136.76	8.68	1.1 h	>3 min	>2 min	Stable, 32.58	85.23	−0.355
Group 3	277	31071.64	8.66	1.1 h	>3 min	>2 min	Stable, 29.54	83.83	−0.377
Group 4	277	31148.77	8.67	1.1 h	>3 min	>2 min	Stable, 30.57	85.56	−0.349
Group 5	277	31200.83	8.48	1.1 h	>3 min	>2 min	Stable, 31.24	82.06	−0.382
Group 6	277	31115.77	8.68	1.1 h	>3 min	>2 min	Stable, 30.11	82.42	−0.360
Group 7	277	31049.70	8.68	1.1 h	>3 min	>2 min	Stable, 30.13	85.23	−0.342
Group 8	281	31502.11	8.42	1.1 h	>3 min	>2 min	Stable, 30.11	83.67	−0.346
Group 9	282	31613.31	8.63	1.1 h	>3 min	>2 min	Stable, 29.67	80.25	−0.371
Group 10	277	31179.04	8.48	1.1 h	>3 min	>2 min	Stable, 30.37	85.47	−0.367
Group 11	281	31698.35	8.63	1.1 h	>3 min	>2 min	Stable, 32.63	81.60	−0.399
BIC mutated model	277	31065.72	8.67	1.1 h	>3 min	>2 min	Stable, 27.11	85.60	−0.341
DTG mutated model	277	31092.75	8.67	1.1 h	>3 min	>2 min	Stable, 27.72	85.60	−0.351
EVG mutated model	277	31055.74	8.81	1.1 h	>3 min	>2 min	Stable, 28.04	83.47	−0.354
RAL mutated model	277	31227.91	8.83	1.1 h	>3 min	>2 min	Stable, 29.72	82.06	−0.386
CAB mutated model	277	31136.80	8.82	1.1 h	>3 min	>2 min	Stable, 27.42	84.87	−0.355
Mutant 1	277	31217.96	8.94	1.1 h	>3 min	>2 min	Stable, 26.73	85.96	−0.354
Mutant 2	277	31187.87	8.94	1.1 h	>3 min	>2 min	Stable, 27.04	85.96	−0.363
Average of all groups and models	277	31.20 KD	8.68	1.1 h	>3 min	>2 min	29.62	84.15	−0.359

### 3.5 Post-translational modification

In terms of phosphorylation sites, no significant difference could be observed between the reference gene and mutant HIV-1 INT proteins (Supplemental Table 3). The most frequent residues for phosphorylation were serine 39, 57, 134, 255 and threonine 67, 93, 112, 135, and tyrosine 100, 171, and 139. The outcomes of disulfide bond prediction (Supplemental Table 4) revealed that bonds 40–43 and 56–65 had a higher frequency than that of other cysteines.

In this study, the presence of N-glycosylation sites (117 and 120), C-glycosylation sites (19), and some O-glycosylation sites (Supplemental Table 5) was among the most frequent predicted sites. Moreover, SUMOylation sites were assessed, and 19 new amino acid targets (Supplemental Table 6) were suggested that might affect HIV replication. According to the ubiquitination data (Supplemental Table 7), K186, K240, and K258 were the most plausible target lysines for ubiquitination.

### 3.6 Secondary structure

**Fig. 3:**
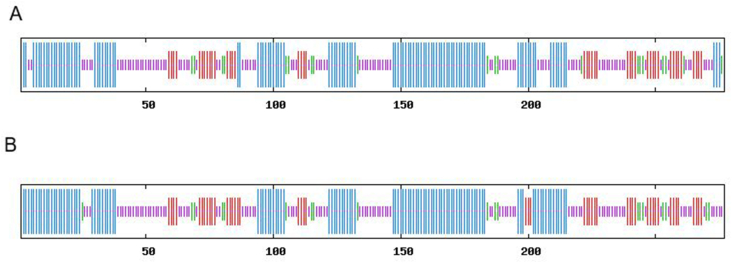
The results of secondary structure prediction of mutant model 1 (A) and reference gene, AB703606 (B).

The results of secondary structure prediction of mutant model 1 (A) and reference gene, AB703606 (B) was analyzed (Fig. 3), and the data displayed the pattern of four secondary structures, alpha helix, extended strand, beta-turn, and random coil, which were similar among different groups and models (Table 10). However, alpha-helix (40.07%) and random coil (36.10%) were the most prominent secondary structures in INT mutated proteins (see Table 10).

**Table 10:** Information of the secondary structures of mutated INT and reference proteins.

Groups and models	Alpha helix (Hh) (%)	Extended strand (Ee) (%)	Beta turn (Tt) (%)	Random coil (Cc) (%)
Reference protein (AB703607)	40.07%	16.97%	6.86%	36.10%
Group 1	38.57%	16.07%	5.36%	40%
Group 2	41.64%	16.37%	7.12%	34.88%
Group 3	38.43%	15.66%	6.41%	39.50%
Group 4	39.15%	15.30%	6.05%	39.50%
Group 5	42.35%	16.37%	4.98%	36.30%
Group 6	39.86%	16.01%	6.05%	38.08%
Group 7	45.13%	16.25%	5.42%	33.21%
Group 8	41.99%	14.95%	6.41%	36.65%
Group 9	43.62%	14.89%	5.67%	35.82%
Group 10	41.16%	16.61%	6.86%	35.38%
Group 11	40.21%	15.66%	5.69%	38.43%
BIC mutated model	43.32%	16.25%	6.86%	33.57%
DTG mutated model	42.60%	16.25%	6.50%	34.66%
EVG mutated model	41.16%	16.97%	7.22%	34.66%
RAL mutated model	42.24%	15.16%	6.14%	36.46%
CAB mutated model	41.52%	15.52%	6.86%	36.10%
Mutant 1	40.79%	16.97%	6.50%	35.74%
Mutant 2	40.79%	16.61%	7.22%	35.38%

### 3.7 Tertiary structure

The best-refined models were regarded for validation analysis; then, the qualified models were employed for docking analysis (Supplemental Table 8).

### 3.8 Molecular docking finding

**Fig. 4:**
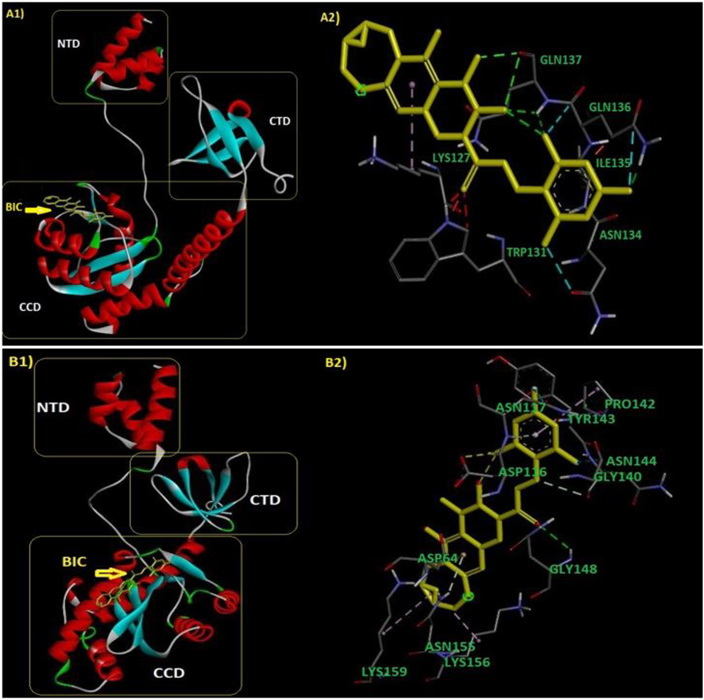
Docking complex of groups 1 and 2 with BIC.

Fig. 4 shows the docking complex of groups 1 and 2 with BIC, and Supplemental Figs. 1–5 illustrated the docking analysis between reference protein and CAB, BIC, DTG, EVG, and RAL that showed the potential amino acids positions in the interaction between reference gene and five INTIs.

**Fig. 5:**
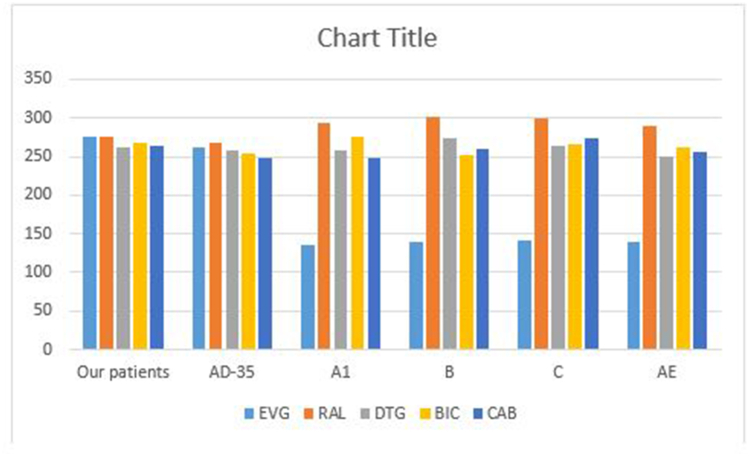
Docking scores of INT reference strains with RAL, EVG, BIC, CAB, and DTG.

To evaluate the effect of RAMs [R263K (major), L74I (minor), G163R, Q95K, S230 N, M50I, V72I, V249I, and L74 M (accessory) mutations] and that of high frequent NOPs (M203I, G134S, V112I, N39S, and Q216H)] on INTIs treatment outcome, we prepared the list of amino acids involved in protein-drug interaction and the related docking scores of INTIs and mutated proteins interaction, as shown in Supplementary Table 9 and Table 11, respectively. E Value in our patients was in the range of 276.60 kcal/mol for EVG and RAL, 268.5, 263.2, and 261.7 kcal/mol for BIC, CAB, and DTG, respectively. Comparing the average docking score of mutated INT proteins and reference genes of the most frequent subtypes including A1, B, C, AE and CRF35-AD showed that the most efficient INTIs in Iranian patients were EVG, RAL, BIC, CAB, and DTG, respectively. Docking scores of INT reference strains with RAL, EVG, BIC, CAB, and DTG are shown in Fig. 5.

**Table 11:** Docking scores of INTIs and mutated proteins interaction.

Groups & Models	BIC	DTG	EVG	CAB	RAL
	Energy	Energy	Energy	Energy	Energy
Group 1	−279.75	−263.28	−269.89	−260.70	−280.42
Group 2	−281.09	−275.45	−298.86	−277.68	−282.65
Group 3	−269.67	−269.82	−288.73	−259.75	−269.67
Group 4	−271.46	−269.31	−279.82	−267.56	−285.49
Group 5 (Major)	−266.42	−246.75	−266.42	−253.26	−264.70
Group 6	−254.53	−253.29	−270.93	−244.46	−269.32
Group 7	−256.82	−250.26	−285.10	−251.20	−261.54
Group 8 (Minor)	−266.75	−258.93	274.68	−265.76	−281.20
Group 9	−258.89	−260.90	−263.84	−263.27	−293.88
Group 10	−257.90	−232.25	−264.36	−251.25	−254.35
Group 11	−281.12	−263.91	−280.13	−269.29	−302.59
BIC mutated model	−276.74	NA	NA	NA	NA
DTG mutated model	NA	−274.03	NA	NA	NA
CAB mutated model	NA	NA	NA	−276.67	NA
EVG mutated model	NA	NA	−284.55	NA	NA
RAL mutated model	NA	NA	NA	NA	−277.45
Reference protein (AB703607)	−253.95	−257.83	−262.09	−248.70	−267.39
Mutant 1	−246.74	−263.98	−281.58	−266.58	−279.26
Mutant 2	−291.42	−275.75	−263.52	−277.57	−271.25
Subtype A1	−276.62	−258.41	−136.10	−248	−294.17
Subtype B	−252.90	−273.30	−138.59	−259.30	−301.06
Subtype C	−266.29	−264.20	−142.16	−274.79	−299.59
Subtype AE	−262.53	−250.33	−140.16	−257	−288.49

NA: Not applicable.

S230 N and Q95K mutations caused a substantial loss in docking energy of groups 7 and 10 for all five INTIs. In addition, reduction in the docking value related to some of the INTIs is displayed in generated models 1, 2, groups 1 and 2. In brief, major, minor, and some of the accessory (Q95K and S230 N) mutations were attributed to E value declined; nonetheless, high frequent NOPs did not influence the binding affinity. Thus, specific mutations in the mentioned groups and models might have a pivotal role in drug resistance.

Although RAMs and NOPs were distributed in different INT domains, all mutated INT proteins were docked through CCD with all INTIs. More importantly, none of the RAMs and NOPs was involved in interaction with INTIs.

Of note, some motifs of amino acids (54–64, 79–81, 111–119, 136–159, and 191–211 aa) and the residues at the positions of 74, 116, 117, 118, 127, 137, 139, 141, and 199 very actively participated in many docking interactions.

### 3.9 B cell epitopes

Among suggested B cell epitopes, only those that were not placed in α-helix and β-sheet structure of protein were analyzed for antigen properties. Finally, the probable antigens were chosen as favorable epitopes (Table 12).

**Table 12:** Predicted B cells epitope in INT protein.

Location	B-cell epitope sequence	Vaxijen (Threshold for this model: 0.4)
40–59	CDKCQLKGEAMHGQVDCSPG	0.6252 (Probable ANTIGEN)
40–70	CDKCQLKGEAIHGQVDCSPGMWQLDCTHLEG	0.6945 (Probable ANTIGEN
75–94	VAVHVASGYIEAEVIPAETG	0.7622 (Probable ANTIGEN)
104–123	LAGRWPVKVVHTDNGSNFTS	0.6504 (Probable ANTIGEN)
185–196	FKRKGGIGGYSA	1.4681 (Probable ANTIGEN)
253–272	DNSDIKVVPRRKAKIIRDYG	0.4488 (Probable ANTIGEN)

### 3.10 Subtyping analysis

**Fig. 6:**
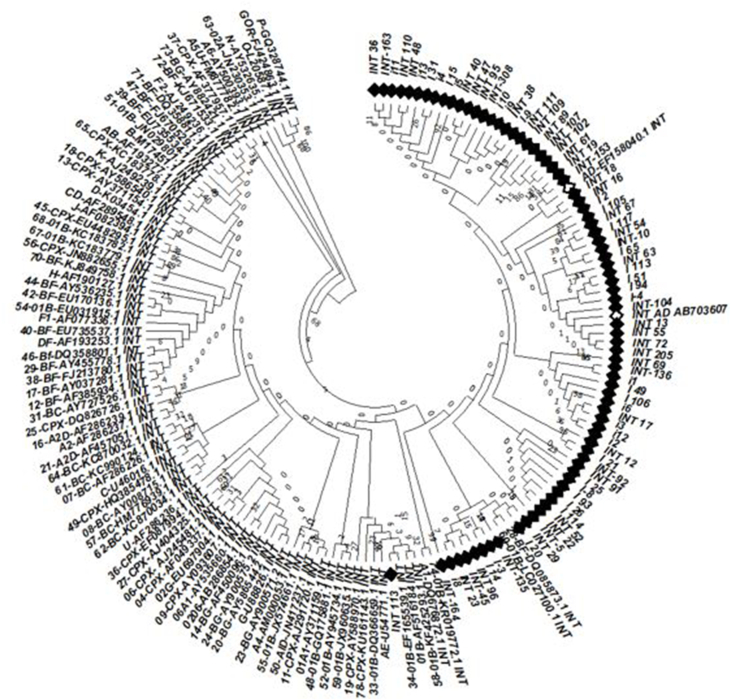
Phylogenic Neighbor-Joining tree of HIV-INT gene sequences of HIV infected patients in Iran was generated with the corresponding INT gene of 89 subtype reference strains.

**Fig. 7:**
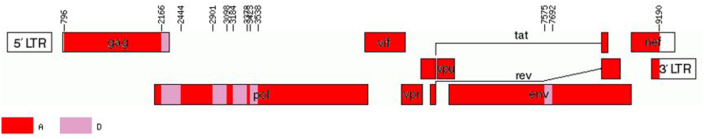
Recombination pattern of CRF-35-AD (accession number AF095). The red color shows subtype A1 and purple indicates subtype D. (For interpretation of the references to color in this figure legend, the reader is referred to the Web version of this article.)

Based on the phylogenic Neighbor-Joining tree of HIV-INT gene sequences of HIV infected patients in Iran which was generated with the corresponding INT gene of 89 subtype reference strains (Fig. 6) and Stanford HIV Subtyping program analysis, CRF35-AD subtype was the major subtype in our samples, but the other six tools introduced A1 as the predominant subtype. HIV Type 1 Subtypes based on the integrase Gene are listed in Table 13. The result of the phylogenetic tree and Stanford HIV Subtyping program was in line with the recombination pattern of CRF-35-AD (accession number AF095). The red color shows subtype A1 and purple indicates subtype D (Fig. 7). The subtyping result of all 78 patients is displayed in supplement Table 10.

**Table 13:** HIV Type 1 Subtypes Based on the integrase Gene.

Subtyping tool	Stanford HIV Subtyping program	REGA HIV-1 Subtyping	NCBI Genotyping	Geno2pheno Integrase	GRADE	Phylogenetic Tree	COMET	jpHMM
HIV subtype	35-AD (94.9%) A (5.1%)	A1 (84.6%) NA (15.4%)	A1 (96.2%) CRF01 (3.8%)	A1 (96.2%) F1 (2.6%) D (1.3%)	A1 (100%)	AD (94.9%) CRF-01B (2.6%) BF (2.6%)	A1 (98.7%) B (1.3%)	A1 (97.4%) A1 & B (1.3%) A1 & K (1.3%)

Conforming to the phylogenetic tree result, some new subtypes including CRF-01B and BF were introduced in Iranian patients for the first time. Of note, our data displayed RAMs found in our patients including V72I, I201V [53,54], M50I, R263K [3], and L74I/M, R263K, S230 N [54] were not specific for any subtype because they were revealed in different subtypes. Comparison of RAMS and NOPs between CRF 35-AD and other subtypes is shown in the Supplementary Table 11.

## 4 Discussion

INTIs regimens are highly efficient antiretroviral agents with long‐lasting potency and reduced toxicity, which are globally accepted in treating naive and experienced individuals. The presence of mutations in INT genes that can change the structural stability and flexibility of these proteins can impact the treatment outcome [2,5].

In this study, we completed several bioinformatics analyses on integrase genes and proteins of the most prevalent subtype in Iran, CRF-35-AD. INT genes failed to amplify in 73 out of 151 plasma samples via an effective RT-nested-PCR. It can be inferred that the emergence of new HIV strains may influence the efficiency of nested-PCR that highlights the importance of whole-genome sequencing of HIV-1 circulating in Iran periodically.

In this study, we found that good adherence even to previous ART regimes was a significant factor to decline the new mutations. Similar to our data, some studies indicated that reduced adherence was linked to some situations and signs such as digestive symptoms [55,56], development of more ART resistance mutations [57,58], more HIV replication, worse virological responses [[59], [60], [61]], age less than 35 or more than 45 [58], declined immunological response [57], shorter time on ART, being IDUs, and advanced HIV stage Thereupon [56].

To control HIV infection and achieve better medical outcome, we need to upgrade the surveillance at three levels: identification and treatment of naïve treatment patients, medication adherence improvement, and regular monitoring of IDUs.

From the present analysis, most of NOPs lied in the catalytic core domain that is target for INTIs [62]; yet, such mutations had limited or no impact on the binding energy. This might be due to the hypothesis that NOPs did not influence the functional structure of INTIs; thus, prescribing such inhibitors can be promising in Iranian patients.

Here, RAMs including major (R263K) [[63], [64], [65]], minor (L74I), and accessory (S230 N, L74 M, Q95K, G163R, M50I, V72I, and V249I) mutations emerged in INT sequences of patients. To the best of our knowledge, there is no report of presence of any major mutations among HIV-infected Iranian patients. Our result was similar to the findings of other studies [66], but was in contrast to some reports displayed R263K [[63], [64], [65]] as the common RAMs in INTI-naive participants [[63], [64], [65], [66]].

The higher docking score represented better binding affinity, indicating the strong attachment of integrase inhibitors to integrase proteins to suppress HIV functions that would contribute to the promising treatment outcome. Yang Luo et al. reported that R263K in combination with four NOPs (S24R, L101 M, G134 N, and K244E) might confer substantial reductions in susceptibility to a wide range of INTIs [67].

R263K appeared with G134 N [68,69] and K244 mutations, it seems the mutation in K244 position can be regarded as one of the HIV escape mechanisms [67].

The E value of interaction between INTIs with integrase genes in group 5 carrying R263Kdeclined for four INTIs: EVG, DTG, RAL, and BIC. This result is in accordance with some studies that observed R263Ksustained a moderate loss in potency against DTG, EVG, RAL, and BIC about 2-fold and had a detrimental effect on CAB susceptibility [70,71].

In addition, one minor RAM, L74I, was found in group 8 that reduced the energy value of BIC, CAB, and EVG. This result is somehow in line with a study that illustrated L74I would have a slight effect on INTIs susceptibility [72]. The previous reports showed L74I contributed to high-level DTG resistance that lowers the potential effect of the first-generation INTIs when combined with some of the major mutations [[73], [74], [75]]. Till now, such minor mutation was reported only in one Iranian patient that was resistant to INTIs [68].

In our samples, accessory mutations including G163R, Q95K, S230 N, M50I, V72I, V249I, and L74 M appeared, which can cause a substantial loss in susceptibility to INTIs alone or in combination. INTI drugs usually select G163R [76]; this mutation was reported in INTI-naive patients at a similar rate in our study [64,77]. On its own, our docking result revealed this mutation not only did not appear to be associated with reduced INTIs susceptibility, but also could even enhance the E value.

Q95K and S230 N mutations were another accessory mutation that had a significant reduction in susceptibility to all INTIs in the CRF35-AD subtype, while no major, minor, or other accessory mutations were identified in this group. Evidence demonstrated S230 N conferred drug resistance and reduced DTG susceptibility by 3-fold.

Some studies revealed that Q95K did not affect INTIs susceptibility or viral replication, but in the presence of some other mutations enhanced resistance to RAL and EVG [66,78].

Other investigations need to be done to clarify the functional role of G163R, and Q95K in response to INTIs.

In addition, the effect of L74 M was evaluated alone or in combination with other mutations. From our data, L74 M retained the docking energy and similar to M50I, may be responsible for the increase in free energy of binding values.

In this study, M50I and R263K were not present in one patient simultaneously. Hence, M50I and R263K were inserted in INT reference, resulting in generation of 5 models, including mutant 1, 2, BIC, DTG, and CAB mutated models followed by the evaluation of the effect of these mutations on the E value. The reduction in energy value was only found in the interaction of BIC drug with BIC-mutated models and mutant 1; it can be concluded that combination of M50I and R263K possibly hurts the sustainability to BIC. In contrast to our data, some studies declared M50I along with R263K was responsible for remarkable loss in DTG [79,80], BIC [81], and CAB [82] susceptibility, but in subtype B.

The high frequent NOPs (M203I, G134S, V112I, and N39P) were identified in our patients not previously reported in two earlier studies in Iran, except for G134S [68]. In our investigation, docking value even increased in the groups of 1–4 carrying M203I, G134S, V112I, and N39P. Therefore, it is suggested that such frequent NOPs will not confer resistance to any of the currently available INTIs. On the other hand, an in vitro study showed V112I was linked to more moderate decreases in viral replication capacity [83,84]; consequently, V112I may contribute to viral fitness to induce resistance in treated individuals [85]. Also, Ceccherini-Silberstein et al. reported that G134S in conjunction with some other mutations resulted in INT catalytic core destabilization and reduction in INTIs efficiency [69,77]. Clinical studies are needed to define whether such mutations at baseline facilitate INTIs resistance in CRF-35AD subtypes.

One of the NOPs in our patients was L101 M, which has not been described previously as RAMs, but L110 M in groups 5, 7, and 9 decreased the docking energy in some INTIs that may be correlated with drug resistance in vivo. Developing the mutant virus carrying L101 M can clarify the influence of this mutation on viral fitness, integration steps, etc.

According to our data, the presence of RAMs and NOPs may cause slight effect on binding energy and drug efficacy; thus, INTIs are likely to be capable of treating Iranian patients infected with the CRF35-AD subtype. This may be due to the location of the amino acid substitutions that were not in the conserved parts of the INT core domain (Asp64, Asp116, and Glu152) [86].

To understand the effect of the NOPs and RAMs on binding affinity, performing molecular dynamics can provide a better explanation; nevertheless, the docking data in this report can also be helpful to declare the efficiency of different INTIs on Iranian patients.

Based on the predicted amino acids involved in molecular docking, some motifs in the integrase proteins (54–64, 79–81, 111–119, 136–159, and 191–211 aa) and some amino acids (74, 116, 117, 118, 127, 137, 139, 141, and 199) were involved in INTIs interaction. Therefore, these conserved regions may provide an absolute opportunity for drug development to target the integrase protein to increase sensitivity to INTIs leading to a favorable clinical treatment.

Based on the affinity of the ligand-receptor complex, EVG and RAL were linked to higher free binding energy in our samples that can be considered for the optimal INTIs treatment in Iranian population.

One of the reasons for the high score in our patients with EVG and RAL could be attributed to the presence of H bonds that exhibited a strong type of interaction.

Viral infection use PTMs to enhance protein antigenicity and virulence properties; plus, increase protein solubilization, interferon response inhibition, and viral replication that have a significant role in viral pathogenesis.

Therefore, host machinery cells remove the PMTs from viral proteins to activate immune response pathways, control the virus replication, and inhibit the viral protein synthesis to eliminate the virus.

INT undergoes multiple PMTs that play versatile roles in the functions of INT and HIV-1 viral replication [87]. Phosphorylation prediction suggested some residues appropriate for phosphorylation that may be required for the interaction of INT with cellular factors that either tether or stimulate the integration into the genome. Our finding showed that the most suggested sites for phosphorylation modification were S255 and S57. A previous study indicated that preventing phosphorylation at the S255 position exhibited more viral infectivity correlated with an increased chance of viral DNA integration. Also, INT phosphorylation at position S57 led to INT stability that is ultimately required for efficient viral replication [88]. On that account, interfering with S255 and S57 phosphorylation may cause lower viral replication and HIV pathogenesis.

Given the different disulfide bonds that can be formed between cysteines, special linkages are shaped according to the energy and structural constraints [89]. Once formed, the disulfide bond covalently locks the structure in place and primarily increases the stability and half-life of the proteins [20].

Therefore, disulfide bonds degradation may degrade viral proteins and provide a new area for antiviral drugs development.

In this report, none of RAMs and NOPs in our sequences provided the new SUMOylation target site. Hence, other factors may have affected the position of SUMOylation. INT is susceptible to be SUMOylated at three SUMOylation sites (45LKGE, 135IKQE, and 243WKQE) on three Lys residues (K46, K136, and K244). INT SUMOylation impairment correlated with a significant drop in integration events and inhibited replication [87,90,91].

Unlike the second SUMOylation site, the first and third SUMOylation sites were conserved in all our samples that can be a desired target for designing drugs to destabilize HIV-INT.

Stabilization of INT is required for efficient genomic interaction which can be done via blockade of ubiquitination associated with proteasomal degradation [92].

Here, different lysines were suggested as ubiquitin targets using bioinformatics tools. Among them, K186 and K240 were essential residues since they play a significant role in the structure and functions of INT protein [93]. To usurp the host-ubiquitin machinery, these lysines should be marked for proteasomal degradation to suppress HIV integration.

Various agents have been categorized as carbohydrate-binding agents (CBAs) that impede virus infection. To suppress the vast majority of viruses, the development of antivirals components to target special deglycosylation sites in viral proteins may provide an absolute opportunity for clinical therapies.

This report defined N, O, and C glycosylation sites and, to the best of our knowledge, the influence of glycosylation on integrase proteins and HIV pathogenesis is not described clearly; thus, experimental studies are needed to elaborate on this matter [94].

The data of ProtParam determined the notable similarity in INT mutated proteins in all patients with reference.

However, the pI in different integrase proteins was slightly different, which can be described by the diversity in the number of basic amino acids, as mutated INT proteins are basic proteins. Accurate prediction of the pI of viruses is beneficial for physical/chemical treatment processes and modeling virus behavior in environment [95]. Moreover, the instability index, an estimation of the stability of a protein in a test tube, confirms they are unstable proteins and a relatively high aliphatic index of INT proteins revealed they are thermostable proteins. Next, the average GRAVY, which calculates a grand average hydropathy of the sequence, illustrates all INT proteins inquired moderately hydrophilic property. The GRAVY value less than zero is an indicator of hydrophilicity, suggesting the hydrophilic nature of INT proteins and the possibility of better interaction with water [96].

Attributing to the short half-lives of proteins assessed by Protparam, all INT proteins in this study were a kind of fast degradation proteins in humans, yeast, and E-coli. The rate of protein degradation is dependent on a few factors such as molecular weight, size, and surface charge [97].

In recent years, HIV-INT recombinant protein were used for different approaches including serological diagnostic methods, therapeutic applications, and vaccine development. There is no general expression system to use optimally for all mentioned purposes;thus, various expression host systems should be applied for each purpose.

The effect of the RAMs and NOPs on the secondary structure was analyzed and our finding revealed that these mutations did not change various properties of the secondary structure. Accordingly, significant changes in the binding pocket of the mutated INT proteins did not happen and drug potency was retained. This finding is in the same line with docking results in this study.

In our study, only epitopes located in β-turn and random coil structures were considered for further analysis because such secondary structures are mostly placed in the surfaces of the protein and are more likely to be favorable for binding to antibodies [98]. However, most of the α-helix and β-sheet structures are located inside proteins, which are difficult to be recognized and bound by antibodies [98]. In comparison to the INT reference, different RAMs and NOPs did not affect the location of secondary structures and B cell epitopes.

Here, the subtyping result of our samples is different according to the kind of applied methods. . From our data, INT gene can be considered as an appropriate region for HIV subtyping in Iranian patients if only the phylogenetic tree or Stanford HIV subtyping program was applied for subtyping.

Subtyping results of our patients showed that CRF-35AD subtype was the most frequent subtype in Iran, which is in agreement with other studies in Iran [31,99,100]. The first molecular study of HIV-1 genotypes in 2006 [101] revealed that Iranian HIV-1 subtype was suggested to be A; but, Sanders-Buell et al. analyzed the mentioned sequences again and found they were indeed AD recombinant subtypes. The later research group declared the Iranian sequences contained a small region of subtype D in the envelope regions [102].

In this report, the phylogenic analysis revealed some new subtypes; CRF-01B and BF; therefore, whole-genome sequencing is essential for such samples and those that were not amplified via RT-nested-PCR to confirm the emergence of the new strains.

Till now, the list and role of RAMs are not identified for the CRF35-AD subtype. By comparing the mutations that developed in our CFR-35 AD subtypes with other subtypes (Supplemental Table 11), it can be inferred RAMs are similar in different subtypes.

No compelling evidence was found that HIV-1 subtype, but effectiveness, toxicities, and tolerability of ART regimens need to be considered in choosing first-line or second-line therapy, in low-income and middle-income countries [103]. However, evaluating the role of different mutations in various HIV-1 subtypes which may cause significant loss in INTIs potency may be beneficial to revise algorithms for resistance tests and optimize the prescription protocol of INTIs [104].

Advanced In-silico researches in drug discovery and vaccine designing have a considerable role in HIV studies that have accelerated the rapid advancement in the production and manufacture of medicine to balance the therapeutic options by clinicians.

In other words, choosing a treatment strategy is attributed to the various factors including fewer side effects, availability, acceptable tolerability, short treatment duration, efficacy, and pan-genotype activity. This study could act as a stepping stone to designing novel experiments:

Applying mentioned bioinformatic approaches may be very useful to examine the efficacy of antiretroviral drugs periodically in different epidemiological settings to anticipate the most efficient HIV inhibitors. Plus, such results can be even more beneficial if all predictions will be evaluated in-vitro. Furthermore, such tools can optimize new drugs to suppress HIV infection that may lead to the restoration of strong immune responses that aim to eliminate the virus.

Moreover, the molecular docking methods enable the researchers to reveal the interaction between host factors and the HIV proteins to suggest the evolutionary relationship between them that is helpful to recognize the virus behavior.

INT post-modifications are essential for HIV pathogenesis; therefore, applying bioinformatics tools to unveil PTM sites, pathways, and the underlying mechanisms can propose pharmacological inhibitors. Accordingly, to disclose PMT issue bioinformatic studies are suggested for new therapeutic approaches.

## 5 Conclusion

Higher E value of EVG and RAL of mutated INT proteins showed these drugs may help achieve optimal treatment response in Iranian patients. Our bioinformatics analysis showed that RAMs and NOPs led to zero to modest loss in INTIs potency, suggesting that INTIs can be considered in the first-line and salvage therapy in treatment of patients infected with CRF35-AD subtype. Among different NOPs, Q95K, S230 N, and L110 M lowered the strength of INTIs docking energy in interaction with INTs that may consider such mutations as the major or minor ones in the CRF35-AD subtype. Various post-translation modifications and B-cell epitope prediction suggested particular target sites and epitope regions for future antiretroviral drugs and vaccines design, respectively. PTM sites and pathways are possible pharmacological targets for new therapeutic approaches. RT-nested-PCR test failed to amplify INT genes in 50% of the samples that might be due to the emergence of new HIV subtypes in Iran; whole-genome sequencing is strongly recommended to clarify this point. More focus on improving the quality of HIV care, good medication adherence to any types of anti-HIV drugs, and treatment of naïve patients along with better management of HIV-positive IDUs are essential factors to achieve continuous HIV care and better medical outcomes in Iranian patients.

## Ethics approval and consent to participate

The ethical permission for this research study was granted by the ethics committee of the 10.13039/501100004320Shiraz University of Medical Sciences with the Certificate Reference Number of REC 270710028RA. All participants signed written informed consent before participating in the study, in accordance with the Declaration of Helsinki.

## Consent for publication

Not applicable.

## Funding

This work was supported by the 10.13039/501100004320Shiraz University of Medical Sciences with the Certificate Reference Number of REC 270710028RA.

## Authors’ contributions

AH: design of the study, conceptualization & supervision,FGH: performing the experiments, FGH, AH, and ZH: gave scientific suggestions and controlled experiments, MJSH, SB and ZH: data collection, NKH, AH, FGH, and ZH: data analyzing AH: resources & funding acquisition; PK, AH and NKH: statistical analysis of data, AH, FGH: writing the original draft; AH: review & editing.All authors have read and approved the final manuscript.

## Declaration of competing interest

The authors declare that they have no known competing financial interests or personal relationships that could have appeared to influence the work reported in this paper.
