# Supplementary material for: First report of computational protein–ligand docking to evaluate susceptibility to HIV integrase inhibitors in HIV-infected Iranian patients
Source: Biochem Biophys Rep. 2022 Mar 29;30:101254. doi: 10.1016/j.bbrep.2022.101254 (PMC8968007; doi:10.1016/j.bbrep.2022.101254)
Supplement: Multimedia component 1 [file mmc1.docx]

**Supplemental Table 1:** Nucleotide and amino acid sequences of mutated INTs genes/proteins

| **Groups/ Models** | **Amino acid Sequence** | **Nucleotide Sequence** |
| --- | --- | --- |
| **Reference: AB703607** | FLDGIDKAQEEHERYHSNWRAMASDFNLPPIVAKEIVANCDKCQLKGEAMHGQVDCSPGMWQLDCTHLEGKVILVAVHVASGYIEAEVIPAETGQETAYFLLKLAGRWPVKVVHTDNGSNFTSAAFKAACWWAGIQQEFGIPYNPQSQGVVESMNKELKKIIGQVREQAEHLKTAVQMAVFIHNFKRKGGIGGYSAGERIIDIIATDIQTKELQKQITKIQNFRVYYRDSRDPLWKGPAKLLWKGEGAVVIQDNSDIKVVPRRKAKIIRDYGKQMAG | TTTTTAGATGGAATAGATAAGGCTCAAGAAGAACATGAAAGATATCACAGTAATTGGAGAGCAATGGCTAGTGATTTTAATCTGCCACCTATAGTAGCAAAGGAAATAGTAGCCAACTGTGATAAATGTCAACTAAAAGGGGAAGCCATGCATGGACAAGTAGACTGTAGTCCAGGGATGTGGCAACTAGATTGCACACATCTAGAAGGAAAAGTAATTCTGGTAGCAGTCCATGTAGCCAGTGGTTATATAGAAGCAGAAGTGATCCCAGCAGAAACAGGGCAGGAGACAGCATACTTTCTGCTAAAATTAGCAGGAAGATGGCCAGTAAAAGTAGTACACACAGACAATGGCAGCAATTTCACCAGCGCTGCATTTAAAGCAGCCTGTTGGTGGGCAGGTATTCAACAGGAATTTGGAATTCCCTACAATCCCCAAAGTCAAGGGGTAGTGGAATCTATGAATAAGGAATTAAAGAAAATCATAGGGCAGGTAAGAGAGCAAGCTGAACACCTTAAGACAGCAGTACAAATGGCAGTATTCATTCACAATTTTAAAAGAAAAGGGGGGATTGGGGGGTACAGTGCAGGGGAAAGAATAATAGATATGATAGCAACAGACATACAAACTAAAGAACTACAAAAACAAATTACAAAAATTCAAAATTTTCGGGTTTATTACAGGGACAGCAGAGATCCACTTTGGAAAGGACCAGCAAAACTACTTTGGAAAGGTGAAGGGGCAGTAGTAATACAGGACAACAGTGACATAAAGGTAGTGCCTAGAAGAAAAGCAAAGATCATTAGGGATTATGGAAAACAGATGGCAGGTGA |
| **Group 1** | FLDGIDKAQEEHERYHSNWRAMASDFNLPPIVAKEIVASCDKCQLKGEAMHGQVDCSPGMWQLDCTHLEGKVILVAVHVASGYIEAEVIPAETGQETAYFLLKLAGRWPVKIVHTDNGSNFTSAAFKAACWWASIQQEFGIPYNPQSQGVVESMNKELKKIIGQVREQAEHLKTAVQMAVFIHNFKRKGGIGGYSAGERIIDIIATDIQAKELQKNITKIQNFRVYYRDSRDPLWKGPAKLLWKGEGAVVIQDNGDIKVVPRRKAKIIRDYGKQMAGDDW | TGCCACCTATAGTAGCAAAGGAAATAGTAGCCAGCTGTGATAAATGTCAACTAAAAGGGGAAGCCATGCATGGACAAGTAGACTGTAGTCCAGGGATGTGGCAATTAGATTGCACACATCTAGAAGGAAAAGTAATTCTGGTAGCAGTCCATGTAGCCAGTGGCTATATAGAAGCAGAAGTGATCCCAGCAGAAACAGGGCAGGAGACAGCATACTTTCTGCTAAAATTAGCAGGAAGATGGCCAGTAAAAATAGTACACACAGACAATGGCAGCAATTTCACCAGCGCTGCATTTAAAGCAGCCTGTTGGTGGGCAAGTATCCAACAGGAATTTGGAATTCCCTACAATCCCCAAAGTCAAGGTGTAGTGGAATCTATGAATAAGGAATTAAAGAAAATCATAGGGCAGGTAAGAGAGCAAGCTGAACACCTTAAGACAGCAGTACAAATGGCAGTATTCATTCACAATTTTAAAAGAAAAGGGGGGATTGGGGGGTACAGTGCAGGGGAAAGGATAATAGATATAATAGCAACAGACATACAAGCTAAAGAACTACAAAAGAACATTACAAAAATTCAAAATTTTCGGGTTTATTACAGGGACAGCAGAGATCCACTTTGGAAAGGACCAGCAAAACTACTCTGGAAAGGTGAAGGGGCAGTAGTAATACAGGACAACGGTGATATAAAGGTAGTGCCTAGAAGAAAAGCAAAGATTATTAGGGATTATGGAAAACAGATGGCAGGTGATGATTGGG |
| **Group 2** | FLDGIDKAQEEHERYHSNWRAMASDFNLPPIVAKEIVASCDKCQLKGEAMHGQVDCSPGMWQLDCTHLEGKVILVAVHVASGYIEAEVIPAETGQETAYFLLKLAGRWPVKIVHTDNGSNFTSAAFKAACWWANIQQEFGIPYNPQSQGVVESMNKELKKIIGQVREQAEHLKTAVQMAVFIHNFKRKGGIGGYSAGERIIDIIATDIQTKELQKQITKIQNFRVYYRDNRDPLWKGPAKLLWKGEGAVVIQDNSDIKVVPRRKAKIIRDYGKQMAGDDCV | ATCTGCCACCTATAGTAGCAAAGGAAATAGTAGCCAGCTGTGATAAATGTCAACTAAAAGGGGAAGCCATGCATGGACAAGTAGACTGTAGTCCAGGGATGTGGCAATTAGATTGCACACATCTAGAAGGAAAAGTAATTCTGGTAGCAGTCCATGTAGCCAGTGGCTATATAGAAGCAGAAGTGATCCCAGCAGAAACAGGGCAGGAGACAGCATACTTTCTGCTAAAATTAGCAGGAAGATGGCCAGTAAAAATAGTACACACAGACAATGGCAGCAATTTCACCAGCGCTGCATTTAAAGCAGCCTGTTGGTGGGCAAATATCCAACAGGAATTTGGAATTCCCTACAATCCCCAAAGTCAAGGAGTGGTGGAATCTATGAATAAGGAATTAAAGAAAATCATAGGGCAGGTAAGAGAGCAAGCTGAACACCTTAAGACAGCAGTACAAATGGCAGTATTCATTCACAATTTTAAAAGAAAAGGGGGGATTGGGGGGTACAGTGCAGGGGAAAGAATAATAGATATAATAGCAACAGACATACAAACTAAAGAACTACAAAAACAAATTACAAAAATTCAAAATTTTCGGGTTTATTACAGGGACAACAGAGATCCACTTTGGAAAGGACCAGCAAAACTACTTTGGAAAGGTGAAGGGGCAGTAGTAATACAGGACAATAGTGATATAAAGGTAGTGCCTAGAAGAAAAGCAAAGATCATTAGGGATTATGGAAAACAGATGGCAGGTGATGATTGTGTGG |
| **Group 3** | FLDGIDKAQEEHERYHSNWRAMASDFNLPPIVAKEIVANCDKCQLKGEAMHGQVDCSPGMWQLDCTHLEGKVILVAVHVASGYIEAEVIPAETGQETAYFLLKLAGRWPVKTVHTDNGSNFTSAAFKAACWWASIQQEYGIPYNPQSQGVVESMNKELKKIIGQVRDQAEHLKTAVQMAVFIHNFKKKGGIGGYSAGERIIDIIATDIQTKELQKQITKIQNFRVYYRDSRDPLWKGPAKLLWKGEGAVVIQDNSDIKVVPRRKAKIIRDYGKQMAGDDCV | CCACCTATAGTAGCAAAGGAAATAGTAGCCAACTGTGATAAATGTCAACTAAAAGGGGAAGCCATGCATGGACAAGTAGACTGTAGTCCAGGAATGTGGCAATTAGATTGCACACATCTAGAAGGAAAAGTAATTCTGGTAGCAGTCCATGTGGCCAGTGGCTATATAGAAGCAGAAGTGATCCCAGCAGAAACAGGGCAGGAGACAGCATATTTTCTGCTAAAATTAGCAGGAAGGTGGCCAGTAAAAACAGTACACACAGACAATGGCAGCAATTTCACCAGCGCTGCATTCAAAGCAGCCTGTTGGTGGGCAAGTATCCAACAGGAATATGGAATTCCCTACAATCCCCAAAGTCAAGGAGTAGTGGAATCTATGAACAAGGAATTAAAGAAAATCATAGGGCAGGTAAGAGATCAAGCTGAACATCTTAAGACAGCAGTACAAATGGCAGTATTCATTCACAATTTTAAAAAGAAAGGGGGGATTGGGGGGTACAGTGCAGGGGAAAGAATAATAGATATAATAGCAACAGACATACAAACTAAAGAACTACAAAAACAAATTACAAAAATTCAAAATTTTCGGGTTTATTACAGGGACAGCAGAGATCCACTTTGGAAAGGACCAGCAAAACTACTCTGGAAAGGTGAAGGGGCAGTAGTAATACAGGACAATAGTGACATAAAGGTAGTGCCTAGAAGAAAAGCAAAGATCATTAGGGATTATGGAAAACAGATGGCAGGTGATGATTGTGTGGC |
| **Group 4** | FLDGIDKAQEEHERYHSNWRAMASDFNLPPIVAKEIVANCDKCQLKGEAMHGQVDCSPGMWQLDCTHLEGKVILVAVHVASGYIEAEVIPAETGQETAYFLLKLAGRWPVKVVHTDNGSNFTSAAFKAACWWASIQQEYGIPYNPQSQGVVESMNKELKKIIGQVREQAEHLKTAVQMAVFIHNFKRKGGIGGYSAGERIIDIIATDIQTKELQKHITKIQNFRVYYRDSRDPIWKGPAKLLWKGEGAVVIQDNSDIKVVPRRKVKIIRDYGKQMAGDDCV | TAGTGATTTTAATCTGCCACCTATAGTAGCAAAGGAAATAGTAGCCAACTGTGATAAATGTCAACTAAAAGGGGAAGCCATGCATGGACAAGTAGACTGTAGTCCAGGAATGTGGCAATTAGATTGCACACATCTAGAAGGAAAAGTAATTCTGGTAGCAGTCCATGTAGCCAGTGGCTATATAGAAGCAGAAGTGATCCCAGCAGAAACAGGGCAGGAGACAGCATACTTTCTGCTAAAATTAGCAGGAAGATGGCCAGTAAAAGTAGTACACACAGACAATGGCAGCAATTTCACCAGCGCTGCATTTAAAGCAGCCTGTTGGTGGGCAAGTATCCAACAGGAATATGGAATTCCCTACAATCCCCAAAGTCAAGGAGTAGTGGAATCTATGAATAAGGAATTAAAGAAAATCATAGGGCAGGTAAGAGAGCAAGCTGAGCACCTTAAGACAGCAGTGCAAATGGCAGTATTCATTCACAATTTTAAAAGAAAAGGGGGGATTGGGGGGTACAGTGCAGGGGAAAGAATAATAGATATAATAGCAACAGACATACAAACTAAAGAATTACAAAAACACATTACAAAAATTCAAAATTTTCGGGTTTATTACAGGGACAGCAGAGATCCAATTTGGAAAGGACCAGCAAAACTACTCTGGAAAGGTGAAGGGGCAGTAGTAATACAGGACAATAGTGATATAAAGGTAGTGCCTAGAAGAAAAGTAAAGATTATTAGGGATTATGGAAAACAGATGGCAGGTGATGATTGTGTGGC |
| **Group 5** | FLDGIDKAQEEHERYHSNWRAMARDFNLPPIVAKEIVANCDKCQLKGEAMHGQVDCSPGMWQLDCTHLEGKVILVAVHVASGYIEAEVIPAETGQETAYFMLKLAGRWPVKVVHTDNGSNFTSAAFKAACWWANIQQEFGIPYNPQSQGVVESMNKELKKIIGQVREQAEHLKTAVQMAVFIHNFKRKGGIGGYSAGERIIDMIATDIQTKELQKQITKIQNFRVYYRDSRDPLWKGPAKLLWEGEGAVVIQDNSDIKVVPRKKAKIIRDYGKQMAGDDCV | GTAGGGATTTTAATCTGCCACCTATAGTAGCAAAGGAAATAGTAGCCAACTGTGATAAATGTCAACTAAAAGGGGAAGCCATGCATGGACAAGTAGACTGTAGTCCAGGGATGTGGCAACTAGATTGCACACATCTAGAAGGAAAAGTAATTTTGGTGGCAGTCCATGTAGCCAGTGGTTATATAGAAGCAGAAGTGATCCCAGCAGAAACAGGGCAGGAGACAGCATACTTCATGCTAAAATTAGCAGGAAGATGGCCAGTAAAAGTAGTACACACAGACAATGGCAGCAATTTCACCAGCGCTGCCTTTAAAGCAGCCTGTTGGTGGGCAAATATCCAACAGGAATTTGGAATTCCCTACAATCCCCAAAGTCAAGGAGTAGTGGAATCTATGAATAAGGAATTAAAGAAAATCATAGGGCAGGTAAGAGAGCAAGCTGAACACCTTAAGACAGCAGTACAAATGGCAGTATTCATTCACAATTTTAAAAGAAAAGGGGGGATTGGGGGGTACAGTGCAGGGGAAAGAATAATAGATATGATAGCAACAGACATACAAACTAAAGAACTACAAAAACAAATTACAAAAATTCAAAATTTTCGGGTTTATTACAGGGACAGCAGAGATCCACTTTGGAAAGGACCAGCAAAACTACTCTGGGAAGGTGAAGGGGCAGTAGTAATACAGGACAATAGTGATATAAAGGTAGTGCCTAGAAAAAAAGCAAAGATCATTAGGGATTATGGAAAACAGATGGCAGGTGATGATTGTGTGGC |
| **Group 6** | FLDGIDKAQEEHERYHSNWRAMASDFNLPPIVAKEIVANCDKCQLKGEAMHGQVDCSPGMWQLDCTHLEGKVIMVAVHVASGYIEAEVIPAETGQETAYFLLKLAGRWPVKIVHTDNGSNFTSAAFKAACWWAGIQQEFGIPYNPQSQGVVESMNKELKKIIGQVREQAEHLKTAVQMAVFIHNFKRKGGIGGYSAGERIIDMIATDIQTKELQKQITKIQNFRVYYRDSRDPLWKGPAKLLWKGEGAVVIQDNSDIKVVPRRKAKIIRDYGKQMAGDDCV | TGCCACCTATAGTAGCAAAGGAAATAGTAGCCAACTGTGATAAATGTCAACTAAAAGGGGAAGCCATGCATGGACAAGTAGACTGTAGTCCAGGGATGTGGCAATTAGATTGCACACATCTAGAAGGAAAAGTAATTATGGTAGCAGTCCATGTAGCCAGTGGCTATATAGAAGCAGAAGTGATCCCAGCAGAAACAGGGCAGGAGACAGCATACTTTCTGTTAAAATTAGCAGGAAGATGGCCAGTAAAAATAGTACACACAGACAATGGCAGCAATTTCACCAGCGCTGCATTTAAAGCAGCCTGTTGGTGGGCAGGTATCCAACAGGAATTTGGAATTCCCTACAATCCCCAAAGTCAAGGAGTAGTGGAATCTATGAATAAGGAATTAAAGAAAATCATAGGGCAGGTAAGAGAGCAAGCTGAACACCTTAAGACAGCAGTACAAATGGCAGTATTCATTCACAATTTTAAAAGAAAAGGGGGGATTGGGGGGTACAGTGCAGGGGAAAGAATAATAGATATGATAGCAACAGACATACAAACTAAAGAACTACAAAAACAAATTACAAAAATTCAAAATTTTCGGGTTTATTACAGGGACAGCAGAGATCCACTTTGGAAAGGACCAGCAAAACTACTCTGGAAAGGTGAAGGGGCAGTAGTAATACAGGACAATAGTGATATAAAGGTAGTGCCTAGAAGAAAAGCAAAGATCATTAGGGATTATGGAAAACAGATGGCAGGTGATGATTGTGTGGC |
| **Group 7** | FLDGIDKAQEEHERYHSNWRAMASDFNLPPIVAKEIVASCDKCQLKGEAMHGQVDCSPGIWQLDCTHLEGKIIMVAVHVASGYIEAEVIPAETGQETAYFILKLAGRWPVKIVHTDNGSNFTSAAVKAACWWAGIQQEFGIPYNPQSQGVVESMNKELKKIIGQVRDQAEHLKTAVQMAVFIHNFKRKGGIGGYSAGERIIDMIATDIQTKELQKQITKIQNFRVYYRDNRDPLWKGPAKLLWKGEGAVVIQDNSDIKVVPRRKAKIIRDYGKQMAG | TGCCACCTATAGTAGCAAAGGAAATAGTAGCCAGCTGTGATAAATGTCAACTAAAAGGGGAAGCCATGCATGGACAAGTAGACTGTAGTCCAGGGATATGGCAATTAGATTGCACACATCTAGAAGGAAAAATAATTATGGTAGCAGTCCATGTAGCCAGTGGCTATATAGAAGCAGAAGTGATCCCAGCAGAAACAGGGCAGGAGACAGCATATTTTATACTAAAATTAGCAGGAAGATGGCCAGTAAAAATAGTACACACAGACAATGGCAGCAATTTCACCAGCGCTGCAGTTAAAGCAGCCTGTTGGTGGGCAGGTATCCAACAGGAATTTGGAATTCCCTACAATCCCCAAAGTCAAGGAGTAGTGGAATCTATGAATAAGGAATTAAAGAAAATCATAGGGCAGGTAAGAGACCAAGCTGAACACCTTAAGACAGCAGTACAAATGGCAGTATTCATTCACAATTTTAAAAGAAAAGGGGGGATTGGGGGGTACAGTGCAGGGGAAAGAATAATAGATATGATAGCAACAGACATACAAACTAAAGAACTACAAAAACAAATTACAAAAATTCAAAATTTTCGGGTTTATTACAGGGACAACAGAGATCCACTTTGGAAAGGACCAGCAAAACTACTTTGGAAAGGTGAAGGGGCAGTGGTAATACAGGACAATAGTGATATAAAAGTAGTGCCTAGAAGAAAAGCAAAGATCATCAGGGATTATGGAAAACAGATGGCAGGTG |
| **Group 8** | FLDGIDKAQEEHERYHSNWRAMASDFNLPPIVAKEIVANCDKCQLKGEAMHGQVDCSPGMWQIDCTHLEGKVIIVAVHVASGYIEAEVISAETGQETAYFLLKLAGRWPVKTVHTDNGSNFTSAAFKAACWWAGIQQEFGIPYNPQSQGVVESMNKELKKIIGQVRDQAEYLKTAVQMAVFIHNFKRKGGIGGYSAGERIIDIIATDIQTKELQKNITKIQKFRVYYRDSRDPLWKGPAKLLWKGEGAVVIQDNSDIKVVPRRKAKIIRDYGKQMAGDDCV | GGACTGTAGTCCAGGGATGTGGCAAATAGATTGCACACATCTAGAAGGAAAAGTAATTATAGTAGCAGTACATGTAGCCAGTGGCTATATAGAAGCAGAAGTGATCTCAGCAGAAACAGGGCAGGAGACAGCATACTTTCTGCTAAAATTAGCAGGAAGATGGCCAGTAAAAACAGTACACACAGACAATGGCAGCAATTTCACCAGCGCTGCATTTAAAGCAGCCTGTTGGTGGGCAGGTATCCAACAGGAATTTGGAATTCCCTACAATCCCCAAAGTCAAGGAGTAGTGGAATCTATGAATAAGGAATTAAAGAAAATCATAGGGCAGGTAAGAGATCAAGCTGAATACCTTAAGACAGCAGTACAAATGGCAGTATTCATTCACAATTTTAAAAGAAAAGGGGGGATTGGGGGGTACAGTGCAGGGGAAAGAATAATAGATATAATAGCAACAGACATACAAACTAAAGAACTACAAAAAAACATTACAAAAATTCAAAAATTTCGGGTTTATTACAGGGACAGCAGAGATCCACTTTGGAAAGGACCAGCAAAACTACTCTGGAAAGGTGAAGGGGCAGTAGTAATACAGGATAATAGTGATATAAAGGTAGTGCCTAGAAGAAAAGCAAAGATCATTAGGGATTATGGAAAACAGATGGCAGGTGATGATTGTGTGGC |
| **Group 9** | FLDGIDKAQEEHERYHSNWRAMASDFNLPPIVAKEIVANCDKCQLKGEAMHGQVDCSPGMWQLDCTRLEGKVIMVAVHVASGYIEAEVIPAETGQETAYFMLKLAGRWPVKVVHTDNGSNFTSAAFKAACWWANVQQEFGIPYNPQSQGVVESMNKELKKIIGQVRDQAEHLKTAVQMAVFIHNFKRKGGIGGYSAGERIIDMIATDIQTKELQKQITKIQNFRVYYRDSRDPLWKGPAKLLWKGEGAVVIQDNSDIKVVPRRKAKIIRDYGKQMAGDGLCG | CCAGGGATGTGGCAATTAGATTGCACACGTCTAGAAGGAAAAGTAATTATGGTAGCAGTCCATGTAGCCAGTGGCTATATAGAAGCAGAAGTGATCCCAGCAGAAACAGGGCAGGAGACAGCATACTTTATGCTAAAATTAGCAGGAAGGTGGCCAGTAAAAGTAGTACACACAGACAATGGCAGCAATTTTACCAGCGCTGCATTTAAAGCAGCCTGTTGGTGGGCAAATGTTCAACAGGAATTTGGAATTCCCTACAATCCCCAAAGTCAAGGAGTAGTGGAATCTATGAATAAGGAATTAAAGAAAATCATAGGGCAGGTAAGAGATCAAGCTGAACACCTTAAGACAGCAGTACAGATGGCAGTATTCATTCACAATTTTAAAAGAAAAGGGGGGATTGGGGGGTACAGTGCAGGGGAAAGAATAATAGATATGATAGCAACAGACATACAAACTAAAGAACTACAAAAACAAATTACAAAAATTCAAAATTTTCGGGTTTATTACAGGGACAGCAGAGATCCACTTTGGAAAGGACCAGCAAAACTACTTTGGAAAGGTGAAGGAGCAGTAGTAATACAGGACAACAGTGATATAAAGGTAGTGCCTAGAAGAAAAGCAAAGATCATTAGGGATTATGGAAAACAGATGGCAGGTGATGGATTGTGTGGCGTACAA |
| **Group 10** | FLDGIDKAQEEHERYHSNWRAMASDFNLPPIVAKEIVANCDKCQLKGEAMHGQVDCSPGMWQLDCTHLEGQVILVAVHVASGYIEAEVIPAETGKETAYFLLKLAGRWPVKLVHTDNGPNFTSDAFKAACWWASIQQEFGIPYNPQSQGVVESMNXELKKIIGQVREQAEHLKTAVQMAVFIHNFKRKGGIGGYSAGERIIDMIATDIQTKELQKQITKIQKFRVYYRDSRDPLWKGPAKLLWKGEGAVVIQDNSDIKVVPRRKAKIIRDYGKQMAG | AGGAAATAGTAGCCAACTGTGATAAATGTCAACTAAAAGGGGAAGCCATGCATGGACAAGTAGACTGTAGTCCAGGGATGTGGCAATTAGATTGCACACATCTAGAAGGACAAGTAATTCTGGTAGCAGTCCATGTAGCCAGTGGTTATATAGAAGCAGAAGTGATCCCAGCAGAAACAGGGAAGGAAACAGCATACTTTCTGCTAAAATTAGCAGGAAGATGGCCAGTAAAATTAGTACACACAGACAATGGCCCCAATTTCACCAGCGATGCATTTAAAGCAGCCTGTTGGTGGGCAAGTATCCAACAGGAATTTGGAATTCCCTACAATCCCCAAAGTCAAGGGGTAGTGGAATCTATGAATAANGAATTAAAGAAAATCATAGGGCAGGTAAGAGAGCAAGCTGAACACCTTAAGACAGCAGTACAAATGGCAGTATTCATTCACAATTTTAAAAGAAAAGGGGGGATTGGGGGGTACAGTGCAGGGGAAAGAATAATAGACATGATAGCAACAGATATACAAACTAAAGAACTACAAAAACAAATTACAAAAATTCAAAAATTTCGGGTTTATTACAGGGACAGCAGAGATCCACTTTGGAAAGGACCAGCAAAACTACTCTGGAAAGGTGAAGGGGCAGTAGTAATACAGGATAATAGTGATATAAAGGTAGTGCCTAGAAGAAAAGCAAAGATCATTAGGGG |
| **Group 11** | FLDGIDKAQEEHERYHSNWRAMASDFNLPPIVAKEIVANCDKCRLKGEAMHGQIDCSPGMWQLDCTHLEGKVILVAVHVASGYIEAEVIPAETGQETAYFLLKLAGRWPVKAVHTDNGSNFTSAAFKAACWWAGTQHEFGIPYNPQSQRVVESMNKELKTILRQVREQAEHLKTAVQMAVFIHNFKRKGGIGGYSAGERIIDMIATDIQTKELQKQITKIQNFRVYYRDSRDPLWKGPAKLLWKGEGAVVIQDNSDIKVVPRRKAKIIRDYGKQMAGDDCV | CTAGTGATTTTAATCTGCCACCTATAGTAGCAAAGGAAATAGTAGCCAACTGTGATAAATGTCGACTAAAAGGGGAAGCCATGCATGGACAAATAGACTGTAGTCCAGGGATGTGGCAATTAGATTGCACACATCTAGAAGGAAAAGTAATTCTGGTAGCAGTCCATGTAGCCAGTGGCTATATAGAAGCAGAAGTGATCCCAGCAGAAACAGGGCAGGAGACAGCATACTTTCTGCTAAAATTAGCAGGAAGATGGCCAGTAAAAGCAGTACACACAGACAATGGCAGCAATTTCACCAGCGCTGCATTTAAAGCAGCCTGTTGGTGGGCAGGTACCCAACACGAATTTGGAATTCCCTACAATCCCCAAAGTCAACGAGTAGTGGAATCTATGAATAAGGAATTAAAGACAATCCTACGGCAGGTAAGAGAGCAAGCTGAACACCTTAAGACAGCAGTACAAATGGCAGTATTCATTCACAATTTTAAAAGAAAAGGGGGGATTGGGGGGTACAGTGCAGGGGAAAGAATAATAGATATGATAGCAACAGACATACAAACTAAAGAACTACAAAAACAAATTACAAAAATTCAAAATTTTCGGGTTTATTACAGGGACAGCAGAGATCCACTTTGGAAAGGACCAGCAAAACTACTTTGGAAAGGTGAAGGGGCAGTAGTAATACAGGACAACAGTGACATAAAGGTAGTGCCTAGAAGAAAAGCAAAGATCATTAGGGATTATGGAAAACAGATGGCAGGTGATGAT |
| **BIC mutated model** | FLDGIDKAQEEHERYHSNWRAMASDFNLPPIVAKEIVANCDKCQLKGEAIHGQVDCSPGMWQLDCTHLEGKIILVAVHVASGYIEAEVIPAETGQETAYFLLKLAGRWPVKVVHTDNGSNFTSAAFKAACWWAGIQQEFGIPYNPQSQGVVESMNKELKKIIGQVREQAEHLKTAVQMAVFIHNFKRKGGIGGYSAGERIIDMIATDIQTKELQKQITKIQNFRVYYRDSRDPLWKGPAKLLWKGEGAIVIQDNSDIKVVPRKKAKIIRDYGKQMAG | TTTCTGGATGGCATTGATAAAGCGCAGGAAGAACATGAACGCTATCATAGCAACTGGCGCGCGATGGCGAGCGATTTTAACCTGCCGCCGATTGTGGCGAAAGAAATTGTGGCGAACTGCGATAAATGCCAGCTGAAAGGCGAAGCGATTCATGGCCAGGTGGATTGCAGCCCGGGCATGTGGCAGCTGGATTGCACCCATCTGGAAGGCAAAATTATTCTGGTGGCGGTGCATGTGGCGAGCGGCTATATTGAAGCGGAAGTGATTCCGGCGGAAACCGGCCAGGAAACCGCGTATTTTCTGCTGAAACTGGCGGGCCGCTGGCCGGTGAAAGTGGTGCATACCGATAACGGCAGCAACTTTACCAGCGCGGCGTTTAAAGCGGCGTGCTGGTGGGCGGGCATTCAGCAGGAATTTGGC  ATTCCGTATAACCCGCAGAGCCAGGGCGTGGTGGAAAGCATGAACAAAGAACTGAAAAAAATTATTGGCCAGGTGCGCGAACAGGCGGAACATCTGAAAACCGCGGTGCAGATGGCGGTGTTTATTCATAACTTTAAACGCAAAGGCGGCATTGGCGGCTATAGCGCGGGCGAACGCATTATTGATATGATTGCGACCGATATTCAGACCAAAGAACTGCAGAAACAGATTACCAAAATTCAGAACTTTCGCGTGTATTATCGCGATAGCCGCGATCCGCTGTGGAAAGGCCCGGCGAAACTGCTGTGGAAAGGCGAAGGCGCGATTGTGATTCAGGATAACAGCGATATTAAAGTGGTGCCGCGCAAAAAAGCGAAAATTATTCGCGATTATGGCAAACAGATGGCGGGC |
| **DTG mutated models** | FLDGIDKAQEEHERYHSNWRAMASDFNLPPIVAKEIVANCDKCQLKGEAIHGQVDCSPGMWQLDCTHLEGKIILVAVHVASGYIEAEVIPAETGQETAYFLLKLAGRWPVKVVHTDNGSNFTSAAFKAACWWAGIQQEFGIPYNPQSQGVVESMNKELKKIIGQVREQAEHLKTAVQMAVFIHNFKRKGGIGGYSAGERIIDMIATDIQTKELQKQITKIQNFRVYYRDNRDPLWKGPAKLLWKGEGAIVIQDNSDIKVVPRKKAKIIRDYGKQMAG | TTTCTGGATGGCATTGATAAAGCGCAGGAAGAACATGAACGCTATCATAGCAACTGGCGCGCGATGGCGAGCGATTTTAACCTGCCGCCGATTGTGGCGAAAGAAATTGTGGCGAACTGCGATAAATGCCAGCTGAAAGGCGAAGCGATTCATGGCCAGGTGGATTGCAGCCCGGGCATGTGGCAGCTGGATTGCACCCATCTGGAAGGCAAAATTATTCTGGTGGCGGTGCATGTGGCGAGCGGCTATATTGAAGCGGAAGTGATTCCGGCGGAAACCGGCCAGGAAACCGCGTATTTTCTGCTGAAACTGGCGGGCCGCTGGCCGGTGAAAGTGGTGCATACCGATAACGGCAGCAACTTTACCAGCGCGGCGTTTAAAGCGGCGTGCTGGTGGGCGGGCATTCAGCAGGAATTTGGC  ATTCCGTATAACCCGCAGAGCCAGGGCGTGGTGGAAAGCATGAACAAAGAACTGAAAAAAATTATTGGCCAGGTGCGCGAACAGGCGGAACATCTGAAAACCGCGGTGCAGATGGCGGTGTTTATTCATAACTTTAAACGCAAAGGCGGCATTGGCGGCTATAGCGCGGGCGAACGCATTATTGATATGATTGCGACCGATATTCAGACCAAAGAACTGCAGAAACAGATTACCAAAATTCAGAACTTTCGCGTGTATTATCGCGATAACCGCGATCCGCTGTGGAAAGGCCCGGCGAAACTGCTGTGGAAAGGCGAAGGCGCGATTGTGATTCAGGATAACAGCGATATTAAAGTGGTGCCGCGCAAAAAAGCGAAAATTATTCGCGATTATGGCAAACAGATGGCGGGC |
| **CAB mutated models** | FLDGIDKAQEEHERYHSNWRAMASDFNLPPIVAKEIVANCDKCQLKGEAIHGQVDCSPGMWQLDCTHLEGKVIMVAVHVASGYIEAEVIPAETGQETAYFLLKLAGRWPVKVVHTDNGSNFTSAAFKAACWWAGIQQEFGIPYNPQSQGVVESMNKELKKIIRQVREQAEHLKTAVQMAVFIHNFKRKGGIGGYSAGERIIDIIATDIQTKELQKQITKIQNFRVYYRDSRDPLWKGPAKLLWKGEGAVVIQDNSDIKVVPRKKAKIIRDYGKQMAG | TTTCTGGATGGCATTGATAAAGCGCAGGAAGAACATGAACGCTATCATAGCAACTGGCGCGCGATGGCGAGCGATTTTAACCTGCCGCCGATTGTGGCGAAAGAAATTGTGGCGAACTGCGATAAATGCCAGCTGAAAGGCGAAGCGATTCATGGCCAGGTGGATTGCAGCCCGGGCATGTGGCAGCTGGATTGCACCCATCTGGAAGGCAAAGTGATTATGGTGGCGGTGCATGTGGCGAGCGGCTATATTGAAGCGGAAGTGATTCCGGCGGAAACCGGCCAGGAAACCGCGTATTTTCTGCTGAAACTGGCGGGCCGCTGGCCGGTGAAAGTGGTGCATACCGATAACGGCAGCAACTTTACCAGCGCGGCGTTTAAAGCGGCGTGCTGGTGGGCGGGCATTCAGCAGGAATTTGGC  ATTCCGTATAACCCGCAGAGCCAGGGCGTGGTGGAAAGCATGAACAAAGAACTGAAAAAAATTATTCGCCAGGTGCGCGAACAGGCGGAACATCTGAAAACCGCGGTGCAGATGGCGGTGTTTATTCATAACTTTAAACGCAAAGGCGGCATTGGCGGCTATAGCGCGGGCGAACGCATTATTGATATTATTGCGACCGATATTCAGACCAAAGAACTGCAGAAACAGATTACCAAAATTCAGAACTTTCGCGTGTATTATCGCGATAGCCGCGATCCGCTGTGGAAAGGCCCGGCGAAACTGCTGTGGAAAGGCGAAGGCGCGGTGGTGATTCAGGATAACAGCGATATTAAAGTGGTGCCGCGCAAAAAAGCGAAAATTATTCGCGATTATGGCAAACAGATGGCGGGC |
| **EVG mutated models** | FLDGIDKAQEEHERYHSNWRAMASDFNLPPIVAKEIVANCDKCQLKGEAMHGQVDCSPGMWQLDCTHLEGKVILVAVHVASGYIEAEVIPAETGKETAYFLLKLAGRWPVKVVHTDNGSNFTSAAFKAACWWAGIQQEFGIPYNPQSQGVVESMNKELKKIIGQVREQAEHLKTAVQMAVFIHNFKRKGGIGGYSAGERIIDMIATDIQTKELQKQITKIQNFRVYYRDSRDPLWKGPAKLLWKGEGAVVIQDNSDIKVVPRKKAKIIRDYGKQMAG | TTTCTGGATGGCATTGATAAAGCGCAGGAAGAACATGAACGCTATCATAGCAACTGGCGCGCGATGGCGAGCGATTTTAACCTGCCGCCGATTGTGGCGAAAGAAATTGTGGCGAACTGCGATAAATGCCAGCTGAAAGGCGAAGCGATGCATGGCCAGGTGGATTGCAGCCCGGGCATGTGGCAGCTGGATTGCACCCATCTGGAAGGCAAAGTGATTCTGGTGGCGGTGCATGTGGCGAGCGGCTATATTGAAGCGGAAGTGATTCCGGCGGAAACCGGCAAAGAAACCGCGTATTTTCTGCTGAAACTGGCGGGCCGCTGGCCGGTGAAAGTGGTGCATACCGATAACGGCAGCAACTTTACCAGCGCGGCGTTTAAAGCGGCGTGCTGGTGGGCGGGCATTCAGCAGGAATTTGGC  ATTCCGTATAACCCGCAGAGCCAGGGCGTGGTGGAAAGCATGAACAAAGAACTGAAAAAAATTATTGGCCAGGTGCGCGAACAGGCGGAACATCTGAAAACCGCGGTGCAGATGGCGGTGTTTATTCATAACTTTAAACGCAAAGGCGGCATTGGCGGCTATAGCGCGGGCGAACGCATTATTGATATGATTGCGACCGATATTCAGACCAAAGAACTGCAGAAACAGATTACCAAAATTCAGAACTTTCGCGTGTATTATCGCGATAGCCGCGATCCGCTGTGGAAAGGCCCGGCGAAACTGCTGTGGAAAGGCGAAGGCGCGGTGGTGATTCAGGATAACAGCGATATTAAAGTGGTGCCGCGCAAAAAAGCGAAAATTATTCGCGATTATGGCAAACAGATGGCGGGC |
| **RAL mutated models** | FLDGIDKAQEEHERYHSNWRAMASDFNLPPIVAKEIVANCDKCQLKGEAMHGQVDCSPGMWQLDCTHLEGKVIMVAVHVASGYIEAEVIPAETGQETAYFLLKLAGRWPVKVVHTDNGSNFTSAAFKAACWWAGIQQEFGIPYNPQSQGVVESMNKELKKIIRQVREQAEHLKTAVQMAVFIHNFKRKGGIGGYSAGERIIDMIATDIQTKELQKQITKIQNFRVYYRDNRDPLWKGPAKLLWKGEGAVVIQDNSDIKVVPRRKAKIIRDYGKQMAG | TTTCTGGATGGCATTGATAAAGCGCAGGAAGAACATGAACGCTATCATAGCAACTGGCGCGCGATGGCGAGCGATTTTAACCTGCCGCCGATTGTGGCGAAAGAAATTGTGGCGAACTGCGATAAATGCCAGCTGAAAGGCGAAGCGATGCATGGCCAGGTGGATTGCAGCCCGGGCATGTGGCAGCTGGATTGCACCCATCTGGAAGGCAAAGTGATTATGGTGGCGGTGCATGTGGCGAGCGGCTATATTGAAGCGGAAGTGATTCCGGCGGAAACCGGCCAGGAAACCGCGTATTTTCTGCTGAAACTGGCGGGCCGCTGGCCGGTGAAAGTGGTGCATACCGATAACGGCAGCAACTTTACCAGCGCGGCGTTTAAAGCGGCGTGCTGGTGGGCGGGCATTCAGCAGGAATTTGGCATTCCGTATAACCCGCAGAGCCAGGGCGTGGTGGAAAGCATGAACAAAGAACTGAAAAAAATTATTCGCCAGGTGCGCGAACAGGCGGAACATCTGAAAACCGCGGTGCAGATGGCGGTGTTTATTCATAACTTTAAACGCAAAGGCGGCATTGGCGGCTATAGCGCGGGCGAACGCATTATTGATATGATTGCGACCGATATTCAGACCAAAGAACTGCAGAAACAGATTACCAAAATTCAGAACTTTCGCGTGTATTATCGCGATAACCGCGATCCGCTGTGGAAAGGCCCGGCGAAACTGCTGTGGAAAGGCGAAGGCGCGGTGGTGATTCAGGATAACAGCGATATTAAAGTGGTGCCGCGCCGCAAAGCGAAAATTATTCGCGATTATGGCAAACAGATGGCGGGC |
| **Mutant 1** | FLDGIDKAQEEHERYHSNWRAMASDFNLPPIVAKEIVASCDKCQLKGEAIHGQVDCSPGMWQLDCTHLEGKIIMVAVHVASGYIEAEVIPAETGKETAYFLLKLAGRWPVKIVHTDNGSNFTSAAFKAACWWASIQQEFGIPYNPQSQGVVESMNKELKKIIRQVREQAEHLKTAVQMAVFIHNFKRKGGIGGYSAGERIIDIIATDIQTKELQKHITKIQNFRVYYRDNRDPLWKGPAKLLWKGEGAIVIQDNSDIKVVPRKKAKIIRDYGKQMAG | TTTCTGGATGGCATTGATAAAGCGCAGGAAGAACATGAACGCTATCATAGCAACTGGCGCGCGATGGCGAGCGATTTTAACCTGCCGCCGATTGTGGCGAAAGAAATTGTGGCGAGCTGCGATAAATGCCAGCTGAAAGGCGAAGCGATTCATGGCCAGGTGGATTGCAGCCCGGGCATGTGGCAGCTGGATTGCACCCATCTGGAAGGCAAAATTATTATGGTGGCGGTGCATGTGGCGAGCGGCTATATTGAAGCGGAAGTGATTCCGGCGGAAACCGGCAAAGAAACCGCGTATTTTCTGCTGAAACTGGCGGGCCGCTGGCCGGTGAAAATTGTGCATACCGATAACGGCAGCAACTTTACCAGCGCGGCGTTTAAAGCGGCGTGCTGGTGGGCGAGCATTCAGCAGGAATTTGGC  ATTCCGTATAACCCGCAGAGCCAGGGCGTGGTGGAAAGCATGAACAAAGAACTGAAAAAAATTATTCGCCAGGTGCGCGAACAGGCGGAACATCTGAAAACCGCGGTGCAGATGGCGGTGTTTATTCATAACTTTAAACGCAAAGGCGGCATTGGCGGCTATAGCGCGGGCGAACGCATTATTGATATTATTGCGACCGATATTCAGACCAAAGAACTGCAGAAACATATTACCAAAATTCAGAACTTTCGCGTGTATTATCGCGATAACCGCGATCCGCTGTGGAAAGGCCCGGCGAAACTGCTGTGGAAAGGCGAAGGCGCGATTGTGATTCAGGATAACAGCGATATTAAAGTGGTGCCGCGCAAAAAAGCGAAAATTATTCGCGATTATGGCAAACAGATGGCGGGC |
| **Mutant 2** | FLDGIDKAQEEHERYHSNWRAMASDFNLPPIVAKEIVASCDKCQLKGEAIHGQVDCSPGMWQLDCTHLEGKIIIVAVHVASGYIEAEVIPAETGKETAYFLLKLAGRWPVKTVHTDNGSNFTSAAFKAACWWASIQQEFGIPYNPQSQGVVESMNKELKKIIRQVREQAEHLKTAVQMAVFIHNFKRKGGIGGYSAGERIIDIIATDIQTKELQKHITKIQNFRVYYRDNRDPLWKGPAKLLWKGEGAIVIQDNSDIKVVPRKKAKIIRDYGKQMAG | TTTCTGGATGGCATTGATAAAGCGCAGGAAGAACATGAACGCTATCATAGCAACTGGCGCGCGATGGCGAGCGATTTTAACCTGCCGCCGATTGTGGCGAAAGAAATTGTGGCGAGCTGCGATAAATGCCAGCTGAAAGGCGAAGCGATTCATGGCCAGGTGGATTGCAGCCCGGGCATGTGGCAGCTGGATTGCACCCATCTGGAAGGCAAAATTATTATTGTGGCGGTGCATGTGGCGAGCGGCTATATTGAAGCGGAAGTGATTCCGGCGGAAACCGGCAAAGAAACCGCGTATTTTCTGCTGAAACTGGCGGGCCGCTGGCCGGTGAAAACCGTGCATACCGATAACGGCAGCAACTTTACCAGCGCGGCGTTTAAAGCGGCGTGCTGGTGGGCGAGCATTCAGCAGGAATTTGGC  ATTCCGTATAACCCGCAGAGCCAGGGCGTGGTGGAAAGCATGAACAAAGAACTGAAAAAAATTATTCGCCAGGTGCGCGAACAGGCGGAACATCTGAAAACCGCGGTGCAGATGGCGGTGTTTATTCATAACTTTAAACGCAAAGGCGGCATTGGCGGCTATAGCGCGGGCGAACGCATTATTGATATTATTGCGACCGATATTCAGACCAAAGAACTGCAGAAACATATTACCAAAATTCAGAACTTTCGCGTGTATTATCGCGATAACCGCGATCCGCTGTGGAAAGGCCCGGCGAAACTGCTGTGGAAAGGCGAAGGCGCGATTGTGATTCAGGATAACAGCGATATTAAAGTGGTGCCGCGCAAAAAAGCGAAAATTATTCGCGATTATGGCAAACAGATGGCGGGC |

**Supplemental Table 2:** Accession number of 89 reference genes applied in phylogenic tree analysis

| **Accession Number** | **Subtype** | **Accession Number** | **Subtype** | **Accession Number** | **Subtype** |
| --- | --- | --- | --- | --- | --- |
| AJ249236.1 | F2 | AJ245481.2 | 06-CPX | AY588970.1 | 19-CPX |
| AJ249239.1 | K | AF286226.1 | 07-BC | AY586544.2 | 20-BG |
| KF425293.1 | 58-01B | AY008715.1 | 08-BC | AF457051.1 | 21-A2D |
| JX960635.1 | 59-01B | AY093607.1 | 09-CPX | AY900571.2 | 23-BG |
| KC990124.1 | 61-BC | AJ291720.1 | 11-CPX | AY900575.2 | 24-BG |
| KC870034.1 | 62-BC | AF385934.1 | 12-BF | DQ826726.1 | 25-CPX |
| AY371159.1 | 22-01A1 | AY371154.1 | 13-CPX | AJ404325.1 | 27-CPX |
| AF516184 | 15-01B | AF450096.1 | 14-BG | DQ085873.1 | 28-BF |
| EU697904.1 | 43-02G | AF286239.1 | 16-A2D | AY455778.1 | 29-BF |
| AF049337.1 | 04-CPX | AY037281.1 | 17-BF | AY727526.1 | 31-BC |
| AY535660.1 | 32-06A1 | AY586540.1 | 18-CPX | DQ366659.1 | 33-01B |
| EF165539.1 | 34-01B | EF087994.1 | 36-CPX | AF377957.1 | 37-CPX |
| FJ213780.1 | 38-BF | EU735534.1 | 39-BF | EU735537.1 | 40-BF |
| EU170136.1 | 42-BF | AY536235.1 | 44-BF | EU448295.1 | 45-CPX |
| DQ358801.1 | 46-BF | FJ670529.1 | 47-BF | GQ175881.1 | 48-01B |
| HQ385478.1 | 49-CPX | JN417237.1 | 50-A1D | JN029801.1 | 51-01B |
| AY945734.1 | 52-01B | EU031915.1 | 54-01B | JX574661.1 | 55-01B |
| JN882655.1 | 56-CPX | HM776939.1 | 57-BC | JN230353.1 | 63-02A |
| KC870032.1 | 64-BC | KC183778.1 | 65-CPX | KC183779.1 | 67-01B |
| KC183782.1 | 68-01B | LC027100.1 | 69-01B | KJ849758.1 | 70-BF |
| DQ358811.1 | 71-BF | KJ671533.1 | 72-BF | AY882421.1 | 73-BG |
| KR019772.1 | 74-01B | KU161143.1 | 78-CPX | AB286854.1 | 30-0206 |
| DQ676872.1 | A1 | AF286237.1 | A2 | AM000053.1 | A4 |
| FM877782.1 | 26-A5U | AY500393.1 | A6 | AF193277.1 | 03-AB |
| EF158040.1 | 35-AD | U54771.1 | 01-AE | M17451.1 | B |
| AF289548.1 | 10-CD | U46016.1 | C | AF193253.1 | 05-DF |
| K03454.1 | D | AF077336.1 | F1 | FJ424863.1 | GOR |
| U88826.1 | G | AF190127.1 | H | AF082394.1 | J |
| AY532635.1 | N | L20587.1 | O | GQ328744.1 | P |
| AF286236.1 | U | AB703607 | 35-AD |  |  |

**Supplemental Table 3:** The predicted phosphorylation sites for mutated INT proteins

| **Groups and Models** | **GPS 5.0 kinase** | **PhosphoSVM** | **Phos3D** | **NetPhos 3.1 Server** |
| --- | --- | --- | --- | --- |
| **Reference: AB703607** | *S (39%):* 17, 24, 57, 81, 119, 123, 147, 153, 195, 230, 255 | *S:* 57, 255, 230, 17, 153, 24, 147, 119, 195, 123, 81 | *S (11):* 17, 24, 57, 81, 119, 123, 147, 153, 195, 230, 255 | *S:* 17, 24, 57, 81, 147, 195, 230 |
|  | *T (32%):* 66, 93, 97, 115, 122, 174, 206, 210, 218 | *T:* 206, 115, 210, 174, 97, 218, 93, 122, 66 | *T (9):* 66, 93, 97, 115, 122, 174, 206, 210, 218 | *T:* 66, 93, 122, 206, 210, 218 |
|  | *Y (29%):* 15, 83, 99, 143, 194, 226, 227, 271 | *Y:* 194, 99, 83, 15, 271, 226, 227, 143 | *Y (8):* 15, 83, 99, 143, 194, 226, 227, 271 | *Y:* 15, 83, 99 |
| **Group 1** | *S (43%):* 17, 24, 39, 57, 81, 119, 123, 134, 147, 153, 195, 230 | *S:* 57, 230, 39, 17, 153, 24, 147, 119, 195, 134, 123, 81 | *S (12):* 17, 24, 39, 57, 81, 119, 123, 134, 147, 153, 195, 230 | *S:* 17, 24, 39, 57, 81, 147, 195, 230 |
|  | *T (29%):* 66, 93, 97, 115, 122, 174, 206, 218 | *T:* 206, 115, 174, 97, 218, 93, 122, 66 | *T (8):* 66, 93, 97, 115, 122, 174, 206, 218 | *T:* 66, 93, 122, 206, 218 |
|  | *Y (29%):* 15, 83, 99, 143, 194, 226, 227, 271 | *Y:* 194, 99, 271, 83, 15, 227, 226, 143 | *Y (8):* 15, 83, 99, 143, 194, 226, 227, 271 | *Y:* 15, 83, 99 |
| **Group 2** | *S (39%):* 17, 24, 39, 57, 81, 119, 123, 147, 153, 195, 255 | *S:* 57, 255, 39, 17, 153, 24, 147, 119, 195, 123, 81 | *S (11):* 17, 24, 39, 57, 81, 119, 123, 147, 153, 195, 255 | *S:* 17, 24, 39, 57, 81, 147, 195 |
|  | *T (32%):* 66, 93, 97, 115, 122, 174, 206, 210, 218 | *T:* 206, 115, 174, 210, 97, 218, 93, 122, 66 | *T (9):* 66, 93, 97, 115, 122, 174, 206, 210, 218 | *T:* 66, 93, 122, 206, 210, 218 |
|  | *Y (29%):* 15, 83, 99, 143, 194, 226, 227, 271 | *Y:* 194, 99, 271, 83, 15, 226, 143, 227 | *Y (8):* 15, 83, 99, 143, 194, 226, 227, 271 | *Y:* 15, 83, 99 |
| **Group 3** | *S (39%):* 17, 24, 57, 81, 119, 123, 134, 147, 153, 195, 230, 255 | *S:* 57, 255, 230, 17, 153, 24, 119, 147, 195, 134, 123, 81 | *S (12):* 17, 24, 57, 81, 119, 123, 134, 147, 153, 195, 230, 255 | *S:* 17, 24, 57, 81, 147, 195, 230 |
|  | *T (32%):* 66, 93, 97, 112, 115, 122, 174, 206, 210, 218 | *T:* 112, 206, 115, 174, 210, 97, 218, 93, 122, 66 | *T (10):* 66, 93, 97, 112, 115, 122, 174, 206, 210, 218 | *T:* 66, 93, 122, 206, 210, 218 |
|  | *Y (29%):* 15, 83, 99, 139, 143, 194, 226, 227, 271 | *Y:* 194, 99, 139, 271, 83, 15, 226, 227, 143 | *Y (9):* 15, 83, 99, 139, 143, 194, 226, 227, 271 | *Y:* 15, 83, 99, 139 |
| **Group 4** | *S (40%):* 17, 24, 57, 81, 119, 123, 134, 147, 153, 195, 230, 255 | *S:* 57, 230, 255, 24, 153, 17, 119, 147, 195, 134, 123,81 | *S (12):* 17, 24, 57, 81, 119, 123, 134, 147, 153, 195, 230, 255 | *S:* 17, 24, 57, 81, 147, 195 |
|  | *T (30%):* 66, 93, 97, 115, 122, 174, 206, 210, 218 | *T:* 206, 115, 210, 174, 97, 218, 93, 122, 66 | *T (9):* 66, 93, 97, 115, 122, 174, 206, 210, 218 | *T:* 66, 93, 122, 206, 210, 218 |
|  | *Y (30%):* 15, 83, 99, 139, 143, 194, 226, 227, 271 | *Y:* 194, 99, 271, 139, 83, 15, 226, 227, 143 | *Y (9):* 15, 83, 99, 139, 143, 194, 226, 227, 271 | *Y:* 15, 83, 99, 139 |
| **Major**  **Group 5** | *S (37%):* 17, 57, 81, 119, 123, 147, 153, 195, 230, 255 | *S:*57,255,230,119,17,153,147,195,123,81 | *S (10):* 17, 57, 81, 119, 123, 147, 153, 195, 230, 255 | *S:* 17, 57, 81, 147, 195, 230 |
|  | *T (33%):* 66, 93, 97, 115, 122, 174, 206, 210, 218 | *T:* 206, 115, 210, 174, 218, 97, 93, 122, 66 | *T (9):* 66, 93, 97, 115, 122, 174, 206, 210, 218 | *T:* 66, 93, 122, 206, 210 , 218 |
|  | *Y (30%):* 15, 83, 99, 143, 194, 226, 227, 271 | *Y:* 194, 99, 83, 271, 15, 143, 226, 227 | *Y (8):* 15, 83, 99, 143, 194, 226, 227, 271 | *Y:* 15, 83, 99 |
| **Minor**  **Group 6** | *S (39%):* 17, 24, 57, 81, 119, 123, 147, 153, 195, 230, 255 | *S:* 57, 255, 230, 24, 17, 153, 147, 119, 195, 123, 81 | *S (11):* 17, 24, 57, 81, 119, 123, 147, 153, 195, 230, 255 | *S:* 17, 24, 57, 81, 147, 195, 230 |
|  | *T (32%):* 66, 93, 97, 115, 122, 174, 206, 210, 218 | *T:* 206, 115, 210, 174, 97, 218, 93, 122, 66 | *T (9):* 66, 93, 97, 115, 122, 174, 206, 210, 218 | *T:* 93, 122, 206, 210, 218 |
|  | *Y (29%):* 15, 83, 99, 143, 194, 226, 227, 271 | *Y:* 99, 194, 83, 271, 15, 143, 226, 227 | *Y (8):* 15, 83, 99, 143, 194, 226, 227, 271 | *Y:* 15, 83, 99 |
| **Group 7** | *S (39%):* 17, 24, 39, 57, 81, 119, 123, 147, 153, 195, 255 | *S:* 57, 255, 39, 153, 147, 24, 17, 119, 195, 123, 81 | *S:* _ | *S:* 17, 24, 39, 57, 81, 147, 195 |
|  | *T (32%):* 66, 93, 97, 115, 122, 174, 206, 210, 218 | *T:* 206, 115, 210, 174, 97, 218, 93, 122, 66 | *T:* _ | *T:* 66, 93, 122, 206, 210, 218 |
|  | *Y (29%):* 15, 83, 99, 143, 194, 226, 227, 271 | *Y:* 99, 194, 83, 15, 271, 226, 143, 227 | *Y:* _ | *Y:* 15, 83, 99 |
| **Group 8** | *S (39%):* 17, 24, 57, 81, 90, 119, 123, 147, 153, 195, 230, 255 | *S:* 57, 230, 255, 153, 147, 24, 17, 119, 195, 123, 81, 90 | *S:* 230 | *S:* 17, 24, 57, 81, 147, 195, 230 |
|  | *T (32%):* 66, 93, 97, 112, 115, 122, 174, 206, 210, 218 | *T:* 112, 206, 115, 210, 174, 97, 218, 93, 122, 66 | *T:* _ | *T:* 93, 122, 206, 210, 218 |
|  | *Y (29%):* 15, 83, 99, 143, 171, 194, 226, 227, 271 | *Y:* 99, 194, 171, 83, 15, 271, 226, 143, 227 | *Y:*_ | *Y:* 15, 83, 99, 171 |
| **Group 9** | *S (39%):* 17, 24, 57, 81, 90, 119, 123, 147, 153, 195, 230, 255 | *S:* 57, 255, 230, 153, 147, 24, 17, 119, 195, 123, 81 | *S:* 230 | *S:* 17, 24, 57, 81, 147, 195, 230 |
|  | *T (32%):* 66, 93, 97, 112, 115, 122, 174, 206, 210, 218 | *T:* 206, 115, 210, 174, 97, 218, 93, 122, 66 | *T:*_ | *T:* 93, 122, 206, 210, 218 |
|  | *Y (29%):* 15, 83, 99, 143, 171, 194, 226, 227, 271 | *Y:* 99, 194, 83, 15, 271, 226, 143, 227 | *Y:*_ | *Y:* 15, 83, 99, 271 |
| **Group 10** | *S (39%):* 17, 24, 57, 81, 123, 134, 147, 153, 195, 230, 255 | *S:* 57, 259, 254, 153, 147, 24, 17, 134, 194, 123, 81 | *S:*230 | *S:* 17, 24, 57, 81, 147, 195, 230 |
|  | *T (32%):* 66, 93, 97, 115, 122, 174, 206, 210, 218 | *T:* 205, 115, 209, 173, 97, 217, 93, 122, 66 | *T:*_ | *T:* 66, 93, 122, 206, 210, 218 |
|  | *Y (29%):* 15, 83, 99, 143, 194, 226, 227, 271 | *Y:* 99, 193, 83, 15, 270, 226, 143, 225 | *Y:*_ | *Y:* 15, 83, 99 |
| **Group 11** | *S (37%):* 17, 24, 57, 81, 119, 123, 147, 153, 195, 230, 255 | *S:* 57, 255, 153, 147, 24, 17, 230, 119, 195, 123, 81 | *S:*230 | *S:* 17, 24, 57, 81, 147, 195, 230 |
|  | *T (37%):* 66, 93, 97, 115, 122, 135, 160, 174, 206, 210, 218 | *T:* 206, 115, 160, 210, 174, 97, 218, 93, 122, 66, 135 | *T:*_ | *T:* 66, 93, 122, 135, 206, 210, 218 |
|  | *Y (27%):* 15, 83, 99, 143, 194, 226, 227, 271 | *Y:* 99, 194, 83, 15, 271, 226, 143, 227 | *Y:*_ | *Y:* 15, 83, 99 |
| **Mutant 1** | *S (41%):* 17, 24, 39, 57, 81, 119, 123, 134, 147, 153, 195, 255 | *S:* 57, 255, 39, 153, 147, 24, 17, 119, 195, 123, 81, 134 | *S:*_ | *S:* 18, 25, 40, 58, 82, 148, 196 |
|  | *T (31%):* 66, 93, 97, 115, 122, 174, 206, 210, 218 | *T:* 206, 115, 210, 174, 97, 218, 93, 122, 66 | *T:*_ | *T:* 67, 94, 123, 135, 207, 211, 219 |
|  | *Y (28%):* 15, 83, 99, 143, 194, 226, 227, 271 | *Y:* 99, 194, 83, 15, 271, 226, 143, 227 | *Y:*_ | *Y:* 16, 84, 100 |
| **Mutant 2** | *S (40%):* 17, 24, 39, 57, 81, 119, 123, 134, 147, 153, 195, 255 | *S:* 57, 255, 39, 153, 147, 24, 17, 119, 195, 123, 81, 134 | *S:*_ | *S:* 17, 24, 39, 57, 81, 147, 195, 230 |
|  | *T (33%):* 66, 93, 97, 112, 115, 122, 174, 206, 210, 218 | *T:* 206, 112, 115, 210, 174, 97, 218, 93, 122, 66 | *T:*_ | *T:* 66, 93, 122, 206, 210, 218 |
|  | *Y (27%):* 15, 83, 99, 143, 194, 226, 227, 271 | *Y:* 99, 194, 83, 15, 271, 226, 143, 227 | *Y:*_ | *Y:* 15, 83, 99 |
| **BIC mutated model** | *S (39%):* 17, 24, 57, 81, 119, 123, 147, 153, 195, 230, 255 | *S:* 57, 255, 153, 147, 24, 17, 230, 119, 195, 123, 81 | *S:* 230 | *S:* 17, 24, 57, 81, 147, 195, 230 |
|  | *T (32%):* 66, 93, 97, 115, 122, 174, 206, 210, 218 | *T:* 206, 115, 210, 174, 97, 218, 93, 122, 66 | *T:*_ | *T:* 66, 93, 122, 135, 206, 210, 218 |
|  | *Y (29%):* 15, 83, 99, 143, 194, 226, 227, 271 | *Y:* 99, 194, 83, 15, 271, 226, 143, 227 | *Y:*_ | *Y:* 15, 83, 99 |
| **DTG mutated model** | *S (37%):* 17, 24, 57, 81, 119, 123, 147, 153, 195, 255 | *S:* 57, 255, 153, 147, 24, 17, 119, 195, 123, 81 | *S:*_ | *S:* 17, 24, 57, 81, 147, 195, 230 |
|  | *T (33%):* 66, 93, 97, 115, 122, 174, 206, 210, 218 | *T:* 206, 115, 210, 174, 97, 218, 93, 122, 66 | *T:*_ | *T:* 66, 93, 122, 206, 210, 218 |
|  | *Y (30%):* 15, 83, 99, 143, 194, 226, 227, 271 | *Y:* 99, 194, 83, 15, 271, 226, 143, 227 | *Y:*_ | *Y:* 15, 83, 99 |
| **EVG mutated model** | *S (39%):* 17, 24, 57, 81, 119, 123, 147, 153, 195, 230, 255 | *S:* 57, 255, 230, 153, 147, 24, 17, 119, 195, 123, 81 | *S:* 230 | *S:* 17, 24, 57, 81, 147, 195, 230 |
|  | *T (32%):* 66, 93, 97, 115, 122, 174, 206, 210, 218 | *T:* 206, 115, 210, 174, 97, 218, 93, 122, 66 | *T:*_ | *T:* 66, 93, 122, 206, 210, 218 |
|  | *Y (29%):* 15, 83, 99, 143, 194, 226, 227, 271 | *Y:* 99, 194, 83, 15, 271, 226, 143, 227 | *Y:*_ | *Y:* 15, 83, 99 |
| **RAL mutated model** | *S (37%):* 17, 24, 57, 81, 119, 123, 147, 153, 195, 255 | *S:* 57, 255, 153, 147, 24, 17, 119, 195, 123, 81 | *S:*_ | *S:* 17, 24, 57, 81, 147, 195, 230 |
|  | *T (33%):* 66, 93, 97, 115, 122, 174, 206, 210, 218 | *T:* 206, 115, 210, 174, 97, 218, 93, 122, 66 | *T: _*  _ | *T:* 66, 93, 122, 206, 210, 218 |
|  | *Y (30%):* 15, 83, 99, 143, 194, 226, 227, 271 | *Y:* 99, 194, 83, 15, 271, 226, 143, 227 | *Y:*_ | *Y:* 15, 83, 99 |
| **CAB mutated model** | *S (39%):* 17, 24, 57, 81, 119, 123, 147, 153, 195, 230, 255 | *S:*57, 255, 230, 153, 147, 24, 17, 119, 195, 123, 81 | *S:*_ | *S:*17, 24, 57, 81, 147, 195, 230 |
|  | *T (32%):* 66, 93, 97, 115, 122, 174, 206, 210, 218 | *T:* 206, 115, 210, 174, 97, 218, 93, 122, 66 | *T:*_ | *T:* 66, 93, 122, 206, 210, 218 |
|  | *Y (29%):* 15, 83, 99, 143, 194, 226, 227, 271 | *Y:* 99, 194, 83, 15, 271, 226, 143, 227 | *Y:*_ | *Y:*15, 83, 99 |

**Supplemental Table 4:** The suggested disulfide bonds in mutated IN proteins

|  | **SCRATCH** | **DIANNA** | **VADAR** | **DbD2** | **PIC server** |
| --- | --- | --- | --- | --- | --- |
| **Reference: AB703607** | No disulfide bond, Cysteines:5  (40,56) & (43, 65) | 40-43, 56-65 | No disulfide bond | No disulfide bond | No disulfide bond |
| **Group1** | No disulfide bond, Cysteines: 5  (40,56) & (43, 65) | 40-65, 43- 56 | No disulfide bond | No disulfide bond | No disulfide bond |
| **Group2** | No disulfide bond, Cysteines: 6  (40,56) & (43, 65) | 40-280, 43-130, 56-65 | No disulfide bond | No disulfide bond | No disulfide bond |
| **Group3** | No disulfide bond, Cysteines: 6  (40,56) & (43, 65) | 40-280, 43-130, 56-65 | No disulfide bond | No disulfide bond | No disulfide bond |
| **Group4** | No disulfide bond, Cysteines: 6  (40,56) & (43, 65) | 40-280, 43-130, 56-65 | No disulfide bond | No disulfide bond | No disulfide bond |
| **Group5** | No disulfide bond, Cysteines: 6  (40,56) & (43, 65) | 40-280, 43-130, 56-65 | No disulfide bond | No disulfide bond | No disulfide bond |
| **Group6** | No disulfide bond, Cysteines: 6  (40,56) & (43, 65) | 40-280, 43-130, 56-65 | No disulfide bond | No disulfide bond | No disulfide bond |
| **Group 7** | No disulfide bond, Cysteines: 5  (40,56) & (43, 65) | 40-43, 56-65 | 40, 43 | 40, 43 | 40-43 |
| **Group 8** | No disulfide bond, Cysteines: 6  (56,65) & (130, 280) | 40-280, 43-130, 56-65 | 43, 40 | 40, 43 | 40-43 |
| **Group 9** | No disulfide bond, Cysteines: 6  (40,56) & (65, 130) | 40-43, 56-281, 65-130 | 43, 40 | 40, 43 | 40-43 |
| **Group 10** | No disulfide bond, Cysteines: 5  (40,56) & (43, 65) | 40-43, 56-65 | 43, 40 | 40, 43 | 40-43 |
| **Group 11** | No disulfide bond, Cysteines: 6  (40, 65) & (43, 56) | 40-280, 43-130, 56-65 | 43, 40 | No disulfide bond | 40-43 |
| **Mutant 1** | No disulfide bond, Cysteines: 5  (40,56) & (43, 65) | 40-65, 43-56 | 40, 43 | 40, 43 | 43, 40 |
| **Mutant 2** | No disulfide bond, Cysteines: 5  (40,56) & (43, 65) | 40-65, 43-56 | 40, 43 | No disulfide bond | 43, 40 |
| **BIC mutated model** | No disulfide bond, Cysteines: 5  (40,56) & (43, 65) | 40-65, 43-56 | 40, 43 | 40, 43 | No disulfide bond |
| **DTG mutated model** | No disulfide bond, Cysteines: 5  (40,56) & (43, 65) | 40-43, 56-65 | 40, 43 | 40, 43 | No disulfide bond |
| **EVG mutated model** | No disulfide bond, Cysteines: 5  (40,56) & (43, 65) | 40-43, 56-65 | 40, 43 | 40, 43 | 40, 43 |
| **RAL mutated model** | No disulfide bond, Cysteines: 5  (40,56) & (43, 65) | 40-43, 56-65 | 40, 43 | 40, 43 | 40, 43 |
| **CAB mutated model** | No disulfide bond, Cysteines: 5  (40,56) & (43, 65) | 40-43, 56-65 | No disulfide bond | 40, 43 | 40, 43 |

**Supplemental Table 5:** The Glycosylation sites predicted for all INT mutated proteins

|  | **O-glycosylation sites** | | | **C-glycosylation sites** | | **N-glycosylation sites** | | |
| --- | --- | --- | --- | --- | --- | --- | --- | --- |
|  | **NetOGlyc 4.0 Server** | **GlycoMine** | **GPP Prediction Server** | **NetCGlyc 1.0 Server** | **GlycoMine** | **NetNGlyc 1.0 Server** | **GPP Prediction Server** | **GlycoMine** |
| **Reference: AB703607** | 218 | 195 | 17SG, 24SG, 81SG, 93TG, 122TG, 123SG, 174TG, 206TG, 210TG, 255SG | _ | 19 | 117, 120 | 27NG, 39NG, 117NG, 120NG, 254NG | 155 |
| **Group 1** | _ | 195 | 17SG, 24SG, 81SG, 93TG, 119SG, 122TG, 123SG, 174TG, 206TG | _ | 19 | 117, 120, 216 | 27NG, 117NG, 120NG, 216NG, 254NG | 155 |
| **Group 2** | _ | 195 | 17SG, 24SG, 81SG, 93TG, 119SG, 122TG, 123SG, 174TG, 206TG, 210TG, 255SG | _ | 19 | 117, 120 | 27NG, 117NG, 120NG, 134NG, 254NG | 155 |
| **Group 3** | _ | 195 | 17SG, 24SG, 93TG, 81SG, 93TG, 112TG, 119SG, 122TG, 123SG, 174TG, 206TG, 210 TG, 255SG | _ | 19 | 117, 120 | 27NG, 39NG, 117NG, 120NG, 254NG | 155 |
| **Group 4** | 218 | 195 | 17SG, 24SG, 81SG, 93TG, 122TG, 123SG, 174TG, 206TG, 210TG, 255SG | _ | 19 | 117, 120 | 27NG, 39NG, 117NG, 120NG,254NG | 155 |
| **Group 5** | 218 | 195 | 17SG, 81SG, 93TG, 122TG, 123SG, 174TG, 206TG, 210TG, 255SG | _ | 61 | 117, 120 | 27NG, 39NG, 117NG, 120NG, 134NG, 254NG, | 155 |
| **Group 6** | 218 | 195 | 17SG, 24SG, 81SG, 93TG, 119SG, 122TG, 123SG, 174TG, 206TG, 210TG, 255SG | _ | 19 | 117, 120 | 27NG, 39NG, 117NG, 120NG, 254NG | 155 |
| **Group 7** | _ | 122 | 17SG, 24SG, 81SG, 93TG, 97TG, 119SG, 122TG, 123SG, 174TG, 206TG, 210TG, 255SG | _ | _ | 117, 120 | 27, 117, 120, 254 | 155 |
| **Group 8** | _ | _ | 17SG, 24SG, 81SG, 90SG, 93TG, 97TG, 112TG, 119SG, 122TG, 123SG, 174TG, 206TG, 210TG, 255SG | _ | _ | 117, 120, 216 | 27, 39, 117, 120, 216, 254 | 155 |
| **Group 9** | _ |  | 17SG, 24SG, 81SG, 93TG, 112TG, 122TG, 123SG, 174TG, 206TG, 210TG, 255SG | _ | _ | 117, 120 | 27, 39, 117, 120, 134, 254 | 155 |
| **Group 10** | _ | _ | 17SG, 24SG, 81SG, 93TG, 97TG, 122TG, 174TG, 206TG, 210TG, 255SG | _ | _ | 120 | 27, 39, 120, 254 | 155 |
| **Group 11** | _ | _ | 17SG, 24SG, 81SG, 119SG, 123SG, 93TG, 122TG, 174TG, 206TG, 210TG, 255SG | _ | _ | 117, 120 | 27, 117, 120, 254 | _ |
| **Mutant 1** | _ | _ | 17SG, 24SG, 81SG, 119SG, 123SG, 66TG, 93TG, 97TG, 122TG, 174TG, 206TG, 210TG, 255SG | _ | _ | 117, 120 | 27, 117, 120, 254 | 155 |
| **Mutant 2** | _ | _ | 17SG, 24SG, 81SG, 119SG, 123SG, 66TG, 93TG, 97TG, 112TG, 122TG, 174TG, 206TG, 210TG, 255SG | _ | _ | 117, 120 | 27, 117, 120, 254 | 155 |
| **BIC mutated model** | 218 | _ | 17SG, 24SG, 81SG, 123SG, 66TG, 93TG, 122TG, 174TG, 206TG, 210TG, 255SG | _ | _ | 117, 120 | 27, 39, 117, 120, 254 | 155 |
| **DTG mutated model** | 218 | _ | 17SG, 24SG, 81SG, 123SG, 66TG, 93TG, 122TG, 174TG, 206TG, 210TG, 255SG | _ | _ | 117, 120 | 27, 39, 117, 120, 254 | 155 |
| **EVG mutated model** | _ | _ | 17SG, 24SG, 81SG, 123SG, 93TG, 97TG, 122TG, 174TG, 206TG, 210TG, 255SG | _ | _ | 117, 120 | 27, 39, 117, 120, 254 | 155 |
| **RAL mutated model** | _ | _ | 17SG, 24SG, 81SG, 123SG, 93TG, 122TG, 174TG, 206TG, 210TG, 255SG | _ | _ | 117, 120 | 27, 39, 117, 120, 254 | 155 |
| **CAB mutated model** | _ | _ | 17SG, 24SG, 81SG, 123SG, 93TG, 122TG, 174TG, 206TG, 210TG, 255SG | _ | _ | 117, 120 | 27, 39, 117, 120, 254 | 155 |

**Supplemental Table 6:** SUMOylation sites suggested for mutated INT proteins

| **Groups** | **JASSA** | **SUMOgo** | **SUMOplot** | **GPS-SUMO** |
| --- | --- | --- | --- | --- |
| **Reference: AB703607** | K71, K258 | 46, 244 | High: 46, Low: 244, 236, 188 | 46, 244, 72-76 |
| **Group1** | K71, K258 | 46, 244 | High: 46, Low: 244, 236, 188 | 46, 244, 72-76, 201-205 |
| **Group2** | K71, K258 | 46, 244 | High: 46, Low: 244, 236, 188 | 46, 244, 72-76 |
| **Group3** | K71, K258 | 46, 244 | High: 46, Low: 244, 236, 188, 187 | 46, 244, 72-76 |
| **Group4** | K71, K258 | 46, 244 | High: 46, Low: 244, 236, 188 | 46, 244, 72-76, 200-204 |
| **Group5** | K71, K258 | 46 | High: 46, Low: 236, 188 | 46, 72-76 |
| **Group6** | K71, K258 | 46, 244 | High: 46, Low: 244, 236, 188 | 46, 244 |
| **Group 7** | K71, K258 | 46, 244 | High: 46, Low: 244, 236, 188 | 46, 244, 72-76 |
| **Group 8** | K71, K258 | 46, 244 | High: 46, Low: 244, 236, 188 | 46, 159, 244, 72-76, 201-205 |
| **Group 9** | K71, K258 | 46, 244 | High: 46, Low: 244, 236, 188 | 46, 244, 72-76 |
| **Group 10** | K258 | 46, 244 | High: 46, Low: 244, 236, 188 | 46, 244, 72-76 |
| **Group 11** | K71, K258 | 46, 244 | High: 46, Low: 244, 236, 188 | 46, 244, 72-76 |
| **Mutant 1** | K71, K258 | 46, 244 | High: 49, Low: 247, 239, 191 | 46, 244, 72-76, 200-204 |
| **Mutant 2** | K71, K258 | 46, 244 | High: 49, Low: 247, 239, 191 | 46, 244, 72-76, 200-204 |
| **BIC mutated model** | K71, K258 | 46, 244 | High: 51, Low: 241, 249, 193 | 46, 244, 72-76 |
| **DTG mutated model** | K71, K258 | 46, 244 | High: 51, Low: 241, 249, 193 | 46, 244, 72-76 |
| **EVG mutated model** | K71, K258 | 46, 244 | High: 51, Low: 241, 249, 193 | 46, 244, 72-76 |
| **RAL mutated model** | K71, K258 | 46, 244 | High: 51, Low: 241, 249, 193 | 46, 244, 72-76 |
| **CAB mutated model** | K71, K258 | 46, 244 | High: 51, Low: 241, 249, 193 | 46, 244, 72-76, 200-204 |

**Supplemental Table 7:** Ubiquitination sites suggested for mutated INT proteins

| **Groups** | **The number of ubiquitination sites and a.a positoins** |
| --- | --- |
| **Reference: AB703607** | 6 (25%): K186, K211, K236, K240, K244, K258 |
| **Group1** | 7 (29.2%): K186, K211, K236, K240, K244, K258, K273 |
| **Group2** | 6  (25%): K186, K211, K236, K240, K244, K258 |
| **Group3** | 6  (24%): K111, K211, K236, K240, K244, K258 |
| **Group4** | 5  (20.8%): K186, K211, K240, K244, K258 |
| **Group5** | 2 (8.3%): K186, K240 |
| **Group6** | 5  (20.8%): K186, K236, K240, K244, K258 |
| **Group 7** | 5  (20.8%): K186, K236, K240, K244, K258 |
| **Group 8** | 7  (28%): K110, K186, K211, K236, K240, K244, K258 |
| **Group 9** | 5   (20.8%): K186, K236, K240, K244, K258 |
| **Group 10** | 6   (25%): K111, K186, K236, K240, K244, K258 |
| **Group 11** | 6  (26.08%): K111, K186, K236, K240, K244, K258 |
| **Mutant 1** | 5   (19.23%): K186, K236, K211, K240, K258 |
| **Mutant 2** | 6  (23.07%): K111, K186, K211, K236, K240, K258 |
| **BIC mutated model** | 4  (16%): K186, K236, K240, K258 |
| **DTG mutated model** | 4  (16%): K186, K236, K240, K258 |
| **EVG mutated model** | 5  (19.23%): K186, K236, K240, K244, K258 |
| **RAL mutated model** | 5  (20.83%): K186, K236, K240, K244, K258 |
| **CAB mutated model** | 6  (24%): K186, K211, K236, K240, K244, K258 |

**Supplemental Table 8:** Validation results of the suggested models for INT Reference and INT mutated proteins

| **Groups and Models** | **QMEAN (Z-score)** | **ERRAT (QMEAN4 Value)** | **PROSA-WEB (Quality Factor: A)** | **Ramachandran** | |
| --- | --- | --- | --- | --- | --- |
|  |  |  |  | **Number of amino acids (Favored%)** | **Number of amino acids (Allowed%)** |
| **Reference (AB703607)** | -0.58 | 96.25 | -6.75 | 89.70% | 8.20% |
| **Group 1** | 0.03 | 94.521 | -6.22 | 90.9% | 6.8% |
| **Group 2** | -0.51 | 99.53 | -6.26 | 94.1% | 5% |
| **Group 3** | -0.17 | 98.2063 | -6.33 | 93.2% | 6.4% |
| **Group 4** | -0.11 | 95.95 | -6.69 | 92% | 6.2% |
| **Group 5** | -0.61 | 97.35 | -6.57 | 91.1% | 8.5% |
| **Group 6** | -1.42 | 95.43 | -6.31 | 88.2% | 8.6% |
| **Group 7** | -0.16 | 99.194 | -7.19 | 92.20% | 7% |
| **Group 8** | -0.62 | 96.457 | -7.2 | 91.50% | 7.30% |
| **Group 9** | 0.36 | 97.255 | -7.33 | 93.10% | 6.50% |
| **Group 10** | -1.42 | 97.119 | -6.84 | 92.20% | 7% |
| **Group 11** | -0.82 | 94.737 | -6.94 | 91.90% | 7.30% |
| **BIC mutated model** | 0.04 | 96.72 | -6.95 | 93.40% | 5.80% |
| **DTG mutated model** | -0.48 | 97.58 | -6.87 | 91.80% | 6.60% |
| **EVG mutated model** | -0.43 | 99.59 | -7.52 | 92.60% | 4.20% |
| **RAL mutated model** | -0.26 | 97.89 | -7.06 | 92.60% | 5.70% |
| **CAB mutated model** | -0.28 | 95.32 | -7.02 | 89.80% | 8.20% |
| **Mutant 1** | -1.13 | 95.78 | -6.54 | 92.20% | 6.90% |
| **Mutant 2** | 0.38 | 97.15 | -6.88 | 95.10% | 4.50% |

**Supplemental Table 9:** The list of amino acids involved in proteins-drugs interaction

| **RAL** | **CAB** | **EVG** | **DTG** | **BIC** |  |
| --- | --- | --- | --- | --- | --- |
| **AA** | **AA** | **AA** | **AA** | **AA** |  |
| HIS51, VAL54, VAL79, SER81, GLY149, VAL150, MET154, HIS183, ARG187, ALA196 | GLN136, GLN137, GLU138, PHE139 | CYS56, PRO58, ALA80  ILE191, ARG199, ASP202, ILE203, THR206 | ASP64, ASP116, PRO145, GLU152, ASN155, LYS156, LYS159 | LYS127, TRP131, ASN134, ILE135, GLN136, GLN137 | **Group 1** |
| CYS56, PRO58, ILE191, ARG199, ILE203, ASP207, THR210, LYS211 | ASP116, ASN117, GLY140, ILE141, ASN144, PRO145, SER147, LYS156, LYS159 | ASP64, THR115, ASP116, ASN117, GLY118, GLY140, PRO142, TYR143, PRO145 | ASP64, ASP116, ASN117, GLY118, PRO142, TYR143, PRO145, GLU152, ASN155, LYS156, LYS159 | ASP64, ASP116, ASN117, GLY140, PRO142, TYR143, ASN144, GLN148, ASN155, LYS156, LYS159 | **Group 2** |
| GLY54, MET126, ARG171, ASP174, THR178 | GLY162, ILE163, TYR166, ARG171, ILE175, THR178, ASP179, THR182 | GLY24, GLN25, VAL26, ASP27, MET32, HIS86, TYR111, GLY112, ILE113, ASN116, GLN118, SER119 | ASP64, ASP116, ASN117  GLY118, PRO142, TYR143, PRO145, GLU152, ASN155, LYS156, LYS159 | ASP27, PRO30, ASP174, ILE175, ASP179 | **Group 3** |
| PRO109 | ASN117, GLY140, ILE141, PRO145, LYS159 | PRO58, GLY59, GLY106, LYS211 | CYS56, PRO58, GLY190, ILE191, GLY192, ARG199, ILE203, ASP207 | ILE191, ARG199, ASP202, ILE203, THR210 | **Group 4** |
| VAL150, ILE191, ARG199, ASP202 | LYS127, TRP131, GLN136, GLN137, PHE139 | LYS127, TRP131, GLN136, GLN137, PHE139 | VAL54, CYS56, ALA80, VAL150, ILE191, TYR194, ARG199 | LYS127, GLN136, GLN137 | **Group 5**  **(Major)** |
| ASP64, ASP116, TYR143, GLU152, LYS156, LYS159 | GLY82, VAL150, SER153, MET154, ARG199 | GLY106, TRP108, LYS111, ASP207, ILE208, LYS211 | SER57, PRO58, GLY59, LYS111, ILE112, ASP207, LYS211 | TRP108 | **Group 6** |
| ARG199, ASP202, THR206, ASP207 | GLY52, VAL79, ALA80, SER81, GLN146, GLY149, SER153 | GLN53, VAL54, VAL79, ALA80, GLY149, GLY190, ILE191 | PRO109, VAL110, ALA133, ASP207, LYS211 | GLU138, PHE139 | **Group 7** |
| GLN146, GLU152 | PRO58, ILE191, GLY192, ARG199, THR206, ASP207, THR210 | CYS65, HIS67, GLU92, ASP116, ASN117, SER119, TYR143 | VAL54, VAL79, SER81  SER153, MET154, HIS183, ARG199 | ASP55, LYS111, GLU138, ASP207, LYS211 | **Group 8 (Minor)** |
| LYS127, GLU138 | TRP131, GLN136, GLN137, PHE139 | SER57, PRO58, GLY59, LYS111, ASP207 | GLN137, PHE139, ILE141 | VAL54, GLU157, HIS183, ARG187, ARG199 | **Group 9** |
| LYS127, GLU138, PHE139 | TRP131, GLN136, GLN137, GLU138, PHE139, ILE141 | ALA105, VAL110, LYS211 | LYS127, GLN137, PHE139, GLY140, ILE141, PRO142 | GLU138, LYS127, PHE139 | **Group 10** |
| ASP64, THR115, ASP116, ASN117, GLY118, PHE121, THR122, GLY140, ILE141, PRO142, PRO145, GLN148 | HIS78, VAL79, ALA80, SER81, VAL150, SER153, GLY192, ARG199 | ASP64, THR115, ASP116, ASN117, GLY118, GLY140, ILE141, PRO142, PRO145, GLN148 | ALA80, SER81, VAL150, GLY190, ILE191, GLY192, ARG199 | GLY190, ILE191, ARG199, ASP202 | **Group 11** |
| NA | NA | NA | NA | THR66, TYR143, GLU152, ASN155 | **BIC mutated model** |
| NA | NA | NA | ASP116, ASN117, ILE141, PRO145, GLU152, ASN155, LYS156 | NA | **DTG mutated model** |
| NA | CYS65, THR66, ASN117, GLU152, ASN155 | NA | NA | NA | **CAB mutated model** |
| NA | NA | GLU92, ASP116, GLY118, SER119, ASN120, TYR143, ASN144 | NA | NA | **EVG mutated model** |
| VAL54, SER153, ARG187, TYR194, SER195, ARG199 | NA | NA | NA | NA | **RAL mutated model** |
| ASP64, ASP116, ILE141, TYR143, ASN144, PRO145, GLN148, VAL151, ASN155 | ARG199, ASP207, THR210, LYS211 | ASP64, ASP116, TYR143, ASN144, PRO145, GLU152, ASN155, LYS159 | ASP64, ASP116, PRO145, GLN148, ASN155, LYS156 | PRO58, ARG199, ASP207, THR210, LYS211 | **Reference (AB703607)** |
| LEU68, LYS71, VAL88, GLU92, THR97, ASP116, ASN117, SER119, ASN120, PHE121, ILE141, TYR143, ASN144, LEU172 | HIS51, VAL54, VAL79, ALA80, SER81, GLY149, VAL150, SER153, MET154, HIS183 | SER153, MET154, ARG187, ARG199 | ASP64, ASP116, ASN117, GLY118, SER119, GLU152, ASN155, LYS156 | SER81, VAL150, ARG187 | **Mutant 1** |
| LYS127, TRP131, SER134, GLN136, GLN137, PHE139 | LYS127, ILE135, GLN136, PHE139, GLY140 | ASP64, HIS67, ASP116, ASN117, PRO142, TYR143, GLU152 | GLN136, GLN137, GLU138, PHE139, ILE141 | GLN137, PHE139, ILE141 | **Mutant 2** |
| VAL54, ASP55, CYS56, SER57, ILE60, VAL79, GLU138 | ASN117, GLY118, TYR143 | SER57 | ALA80, TRP108, LYS111, ILE200, ILE203, ILE204, ASP207 | CYS56, ALA80, HIS183, ARG187, ILE191, ALA196 | **Subtype A1** |
| VAL54, CYS56, SER57, ILE60, VAL79, HIS114, GLU138 | ASP64, ASN117, GLY118, ASN155, LYS156 | TRP61, VAL79 | ASP64, PRO142, GLU152, LYS156 | PRO58, TRP61, HIS78, TYR83, TRP108, ILE200, ASP207 | **Subtype B** |
| GLY52, GLN53, VAL54, ASN55, CYS56, SER57, GLY59, ILE60 | HIS78, ILE204, ILE208, LYS211 | ALA80 | ILE208 | PRO58, ARG111, ILE208, LYS211 | **Subtype C** |
| GLN53, VAL54, SER57, ILE60 | PRO142 | SER57, SER81 | ILE141, PRO142 | ASP116, PRO142 | **Subtype AE** |

NA: Not applicable

**Supplemental Table 10:** Subtyping results of 78 patients using 8 different reliable tools

| **Samples** | **Standford HIV**  **Subtyping program** | **REGA HIV-1 Subtyping** | **NCBI Genotyping** | **Geno2pheno Integrase** | **HIV-GRADE** | **Phylogenetic Tree** | **COMET** | **jpHMM** | **Frequency** |
| --- | --- | --- | --- | --- | --- | --- | --- | --- | --- |
| **1** | 35_AD | [(A1)](http://dbpartners.stanford.edu:8080/RegaSubtyping/stanford-hiv/typingtool/job/1018240805/filter-HIV-1%20Subtype%20A%20(A1)) | [(A1)](http://dbpartners.stanford.edu:8080/RegaSubtyping/stanford-hiv/typingtool/job/1018240805/filter-HIV-1%20Subtype%20A%20(A1)) | [(A1)](http://dbpartners.stanford.edu:8080/RegaSubtyping/stanford-hiv/typingtool/job/1018240805/filter-HIV-1%20Subtype%20A%20(A1)) | A1 | AD | [(A1)](http://dbpartners.stanford.edu:8080/RegaSubtyping/stanford-hiv/typingtool/job/1018240805/filter-HIV-1%20Subtype%20A%20(A1))  Check for 35-AD | [(A1)](http://dbpartners.stanford.edu:8080/RegaSubtyping/stanford-hiv/typingtool/job/1018240805/filter-HIV-1%20Subtype%20A%20(A1)) | A1 (6), 35-AD (2) |
| **2** | 35_AD | (A1) | CRF01 | (D) | A1 | AD | [(A1)](http://dbpartners.stanford.edu:8080/RegaSubtyping/stanford-hiv/typingtool/job/1018240805/filter-HIV-1%20Subtype%20A%20(A1)) | [(A1)](http://dbpartners.stanford.edu:8080/RegaSubtyping/stanford-hiv/typingtool/job/1018240805/filter-HIV-1%20Subtype%20A%20(A1)) | A1 (4), 35-AD (1), D (1), CRF01 (2), BF (1) |
| **3** | 35_AD | [(A1)](http://dbpartners.stanford.edu:8080/RegaSubtyping/stanford-hiv/typingtool/job/1018240805/filter-HIV-1%20Subtype%20A%20(A1)) | [(A1)](http://dbpartners.stanford.edu:8080/RegaSubtyping/stanford-hiv/typingtool/job/1018240805/filter-HIV-1%20Subtype%20A%20(A1)) | [(A1)](http://dbpartners.stanford.edu:8080/RegaSubtyping/stanford-hiv/typingtool/job/1018240805/filter-HIV-1%20Subtype%20A%20(A1)) | A1 | AD | [(A1)](http://dbpartners.stanford.edu:8080/RegaSubtyping/stanford-hiv/typingtool/job/1018240805/filter-HIV-1%20Subtype%20A%20(A1))  Check for 35-AD | [(A1)](http://dbpartners.stanford.edu:8080/RegaSubtyping/stanford-hiv/typingtool/job/1018240805/filter-HIV-1%20Subtype%20A%20(A1)) | A1 (6), 35-AD (2) |
| **4** | 35_AD | [(A1)](http://dbpartners.stanford.edu:8080/RegaSubtyping/stanford-hiv/typingtool/job/1018240805/filter-HIV-1%20Subtype%20A%20(A1)) | [(A1)](http://dbpartners.stanford.edu:8080/RegaSubtyping/stanford-hiv/typingtool/job/1018240805/filter-HIV-1%20Subtype%20A%20(A1)) | [(A1)](http://dbpartners.stanford.edu:8080/RegaSubtyping/stanford-hiv/typingtool/job/1018240805/filter-HIV-1%20Subtype%20A%20(A1)) | A1 | AD | [(A1)](http://dbpartners.stanford.edu:8080/RegaSubtyping/stanford-hiv/typingtool/job/1018240805/filter-HIV-1%20Subtype%20A%20(A1))  Check for 35-AD | [(A1)](http://dbpartners.stanford.edu:8080/RegaSubtyping/stanford-hiv/typingtool/job/1018240805/filter-HIV-1%20Subtype%20A%20(A1)) | A1 (6), 35-AD (2) |
| **5** | 35_AD | [(A1)](http://dbpartners.stanford.edu:8080/RegaSubtyping/stanford-hiv/typingtool/job/1018240805/filter-HIV-1%20Subtype%20A%20(A1)) | [(A1)](http://dbpartners.stanford.edu:8080/RegaSubtyping/stanford-hiv/typingtool/job/1018240805/filter-HIV-1%20Subtype%20A%20(A1)) | [(A1)](http://dbpartners.stanford.edu:8080/RegaSubtyping/stanford-hiv/typingtool/job/1018240805/filter-HIV-1%20Subtype%20A%20(A1)) | A1 | AD | [(A1)](http://dbpartners.stanford.edu:8080/RegaSubtyping/stanford-hiv/typingtool/job/1018240805/filter-HIV-1%20Subtype%20A%20(A1))  Check for 35-AD | [(A1)](http://dbpartners.stanford.edu:8080/RegaSubtyping/stanford-hiv/typingtool/job/1018240805/filter-HIV-1%20Subtype%20A%20(A1)) | A1 (6), 35-AD (2) |
| **6** | 35_AD | [(A1)](http://dbpartners.stanford.edu:8080/RegaSubtyping/stanford-hiv/typingtool/job/1018240805/filter-HIV-1%20Subtype%20A%20(A1)) | [(A1)](http://dbpartners.stanford.edu:8080/RegaSubtyping/stanford-hiv/typingtool/job/1018240805/filter-HIV-1%20Subtype%20A%20(A1)) | [(A1)](http://dbpartners.stanford.edu:8080/RegaSubtyping/stanford-hiv/typingtool/job/1018240805/filter-HIV-1%20Subtype%20A%20(A1)) | A1 | AD | [(A1)](http://dbpartners.stanford.edu:8080/RegaSubtyping/stanford-hiv/typingtool/job/1018240805/filter-HIV-1%20Subtype%20A%20(A1))  Check for 35-AD | [(A1)](http://dbpartners.stanford.edu:8080/RegaSubtyping/stanford-hiv/typingtool/job/1018240805/filter-HIV-1%20Subtype%20A%20(A1)) | A1 (6), 35-AD (2) |
| **7** | 35_AD | NA | [(A1)](http://dbpartners.stanford.edu:8080/RegaSubtyping/stanford-hiv/typingtool/job/1018240805/filter-HIV-1%20Subtype%20A%20(A1)) | [(F1)](http://dbpartners.stanford.edu:8080/RegaSubtyping/stanford-hiv/typingtool/job/1018240805/filter-HIV-1%20Subtype%20A%20(A1)) | A1 | AD | (B)  Check for 71_BF1 | [(A1)](http://dbpartners.stanford.edu:8080/RegaSubtyping/stanford-hiv/typingtool/job/1018240805/filter-HIV-1%20Subtype%20A%20(A1)) | A1 (3), 35-AD (2), F1 (1), B/71-BF1 (1), NA (1) |
| **8** | 35_AD | [(A1)](http://dbpartners.stanford.edu:8080/RegaSubtyping/stanford-hiv/typingtool/job/1018240805/filter-HIV-1%20Subtype%20A%20(A1)) | [(A1)](http://dbpartners.stanford.edu:8080/RegaSubtyping/stanford-hiv/typingtool/job/1018240805/filter-HIV-1%20Subtype%20A%20(A1)) | [(A1)](http://dbpartners.stanford.edu:8080/RegaSubtyping/stanford-hiv/typingtool/job/1018240805/filter-HIV-1%20Subtype%20A%20(A1)) | A1 | AD | [(A1)](http://dbpartners.stanford.edu:8080/RegaSubtyping/stanford-hiv/typingtool/job/1018240805/filter-HIV-1%20Subtype%20A%20(A1))  Check for 35-AD | [(A1)](http://dbpartners.stanford.edu:8080/RegaSubtyping/stanford-hiv/typingtool/job/1018240805/filter-HIV-1%20Subtype%20A%20(A1)) | A1 (6), 35-AD (2) |
| **9** | 35_AD | [(A1)](http://dbpartners.stanford.edu:8080/RegaSubtyping/stanford-hiv/typingtool/job/1018240805/filter-HIV-1%20Subtype%20A%20(A1)) | [(A1)](http://dbpartners.stanford.edu:8080/RegaSubtyping/stanford-hiv/typingtool/job/1018240805/filter-HIV-1%20Subtype%20A%20(A1)) | [(A1)](http://dbpartners.stanford.edu:8080/RegaSubtyping/stanford-hiv/typingtool/job/1018240805/filter-HIV-1%20Subtype%20A%20(A1)) | A1 | AD | [(A1)](http://dbpartners.stanford.edu:8080/RegaSubtyping/stanford-hiv/typingtool/job/1018240805/filter-HIV-1%20Subtype%20A%20(A1))  Check for 35-AD | [(A1)](http://dbpartners.stanford.edu:8080/RegaSubtyping/stanford-hiv/typingtool/job/1018240805/filter-HIV-1%20Subtype%20A%20(A1)) | A1 (6), 35-AD (2) |
| **10** | 35_AD | NA | [(A1)](http://dbpartners.stanford.edu:8080/RegaSubtyping/stanford-hiv/typingtool/job/1018240805/filter-HIV-1%20Subtype%20A%20(A1)) | [(A1)](http://dbpartners.stanford.edu:8080/RegaSubtyping/stanford-hiv/typingtool/job/1018240805/filter-HIV-1%20Subtype%20A%20(A1)) | A1 | AD | [(A1)](http://dbpartners.stanford.edu:8080/RegaSubtyping/stanford-hiv/typingtool/job/1018240805/filter-HIV-1%20Subtype%20A%20(A1))  Check for 35-AD | [(A1)](http://dbpartners.stanford.edu:8080/RegaSubtyping/stanford-hiv/typingtool/job/1018240805/filter-HIV-1%20Subtype%20A%20(A1)) | A1 (5), 35-AD (1), NA (1), CRF69-01B (1), BF (1) |
| **11** | 35_AD | [(A1)](http://dbpartners.stanford.edu:8080/RegaSubtyping/stanford-hiv/typingtool/job/1018240805/filter-HIV-1%20Subtype%20A%20(A1)) | [(A1)](http://dbpartners.stanford.edu:8080/RegaSubtyping/stanford-hiv/typingtool/job/1018240805/filter-HIV-1%20Subtype%20A%20(A1)) | [(A1)](http://dbpartners.stanford.edu:8080/RegaSubtyping/stanford-hiv/typingtool/job/1018240805/filter-HIV-1%20Subtype%20A%20(A1)) | A1 | AD | [(A1)](http://dbpartners.stanford.edu:8080/RegaSubtyping/stanford-hiv/typingtool/job/1018240805/filter-HIV-1%20Subtype%20A%20(A1))  Check for 35-AD | [(A1)](http://dbpartners.stanford.edu:8080/RegaSubtyping/stanford-hiv/typingtool/job/1018240805/filter-HIV-1%20Subtype%20A%20(A1)) | A1 (6), 35-AD (2) |
| **12** | 35_AD | [(A1)](http://dbpartners.stanford.edu:8080/RegaSubtyping/stanford-hiv/typingtool/job/1018240805/filter-HIV-1%20Subtype%20A%20(A1)) | [(A1)](http://dbpartners.stanford.edu:8080/RegaSubtyping/stanford-hiv/typingtool/job/1018240805/filter-HIV-1%20Subtype%20A%20(A1)) | [(A1)](http://dbpartners.stanford.edu:8080/RegaSubtyping/stanford-hiv/typingtool/job/1018240805/filter-HIV-1%20Subtype%20A%20(A1)) | A1 | AD | [(A1)](http://dbpartners.stanford.edu:8080/RegaSubtyping/stanford-hiv/typingtool/job/1018240805/filter-HIV-1%20Subtype%20A%20(A1))  Check for 35-AD | [(A1)](http://dbpartners.stanford.edu:8080/RegaSubtyping/stanford-hiv/typingtool/job/1018240805/filter-HIV-1%20Subtype%20A%20(A1)) | A1 (6), 35-AD (2) |
| **13** | 35_AD | [(A1)](http://dbpartners.stanford.edu:8080/RegaSubtyping/stanford-hiv/typingtool/job/1018240805/filter-HIV-1%20Subtype%20A%20(A1)) | [(A1)](http://dbpartners.stanford.edu:8080/RegaSubtyping/stanford-hiv/typingtool/job/1018240805/filter-HIV-1%20Subtype%20A%20(A1)) | [(A1)](http://dbpartners.stanford.edu:8080/RegaSubtyping/stanford-hiv/typingtool/job/1018240805/filter-HIV-1%20Subtype%20A%20(A1)) | A1 | AD | [(A1)](http://dbpartners.stanford.edu:8080/RegaSubtyping/stanford-hiv/typingtool/job/1018240805/filter-HIV-1%20Subtype%20A%20(A1))  Check for 35-AD | [(A1)](http://dbpartners.stanford.edu:8080/RegaSubtyping/stanford-hiv/typingtool/job/1018240805/filter-HIV-1%20Subtype%20A%20(A1)) | A1 (6), 35-AD (2) |
| **14** | 35_AD | [(A1)](http://dbpartners.stanford.edu:8080/RegaSubtyping/stanford-hiv/typingtool/job/1018240805/filter-HIV-1%20Subtype%20A%20(A1)) | [(A1)](http://dbpartners.stanford.edu:8080/RegaSubtyping/stanford-hiv/typingtool/job/1018240805/filter-HIV-1%20Subtype%20A%20(A1)) | [(A1)](http://dbpartners.stanford.edu:8080/RegaSubtyping/stanford-hiv/typingtool/job/1018240805/filter-HIV-1%20Subtype%20A%20(A1)) | A1 | AD | [(A1)](http://dbpartners.stanford.edu:8080/RegaSubtyping/stanford-hiv/typingtool/job/1018240805/filter-HIV-1%20Subtype%20A%20(A1))  Check for 35-AD | [(A1)](http://dbpartners.stanford.edu:8080/RegaSubtyping/stanford-hiv/typingtool/job/1018240805/filter-HIV-1%20Subtype%20A%20(A1)) | A1 (6), 35-AD (2) |
| **15** | 35_AD | [(A1)](http://dbpartners.stanford.edu:8080/RegaSubtyping/stanford-hiv/typingtool/job/1018240805/filter-HIV-1%20Subtype%20A%20(A1)) | [(A1)](http://dbpartners.stanford.edu:8080/RegaSubtyping/stanford-hiv/typingtool/job/1018240805/filter-HIV-1%20Subtype%20A%20(A1)) | [(A1)](http://dbpartners.stanford.edu:8080/RegaSubtyping/stanford-hiv/typingtool/job/1018240805/filter-HIV-1%20Subtype%20A%20(A1)) | A1 | AD | [(A1)](http://dbpartners.stanford.edu:8080/RegaSubtyping/stanford-hiv/typingtool/job/1018240805/filter-HIV-1%20Subtype%20A%20(A1))  Check for 35-AD | [(A1)](http://dbpartners.stanford.edu:8080/RegaSubtyping/stanford-hiv/typingtool/job/1018240805/filter-HIV-1%20Subtype%20A%20(A1)) | A1 (6), 35-AD (2) |
| **16** | 35_AD | [(A1)](http://dbpartners.stanford.edu:8080/RegaSubtyping/stanford-hiv/typingtool/job/1018240805/filter-HIV-1%20Subtype%20A%20(A1)) | [(A1)](http://dbpartners.stanford.edu:8080/RegaSubtyping/stanford-hiv/typingtool/job/1018240805/filter-HIV-1%20Subtype%20A%20(A1)) | [(F1)](http://dbpartners.stanford.edu:8080/RegaSubtyping/stanford-hiv/typingtool/job/1018240805/filter-HIV-1%20Subtype%20A%20(A1)) | A1 | BF | [(A1)](http://dbpartners.stanford.edu:8080/RegaSubtyping/stanford-hiv/typingtool/job/1018240805/filter-HIV-1%20Subtype%20A%20(A1))  Check for 35-AD | [(A1)](http://dbpartners.stanford.edu:8080/RegaSubtyping/stanford-hiv/typingtool/job/1018240805/filter-HIV-1%20Subtype%20A%20(A1)) | A1 (5), 35-AD (1), F1 (1), CRF69-01B (1), BF (1) |
| **17** | 35_AD | [(A1)](http://dbpartners.stanford.edu:8080/RegaSubtyping/stanford-hiv/typingtool/job/1018240805/filter-HIV-1%20Subtype%20A%20(A1)) | [(A1)](http://dbpartners.stanford.edu:8080/RegaSubtyping/stanford-hiv/typingtool/job/1018240805/filter-HIV-1%20Subtype%20A%20(A1)) | [(A1)](http://dbpartners.stanford.edu:8080/RegaSubtyping/stanford-hiv/typingtool/job/1018240805/filter-HIV-1%20Subtype%20A%20(A1)) | A1 | AD | [(A1)](http://dbpartners.stanford.edu:8080/RegaSubtyping/stanford-hiv/typingtool/job/1018240805/filter-HIV-1%20Subtype%20A%20(A1))  Check for 35-AD | [(A1)](http://dbpartners.stanford.edu:8080/RegaSubtyping/stanford-hiv/typingtool/job/1018240805/filter-HIV-1%20Subtype%20A%20(A1)) | A1 (6), 35-AD (2) |
| **18** | 35_AD | [(A1)](http://dbpartners.stanford.edu:8080/RegaSubtyping/stanford-hiv/typingtool/job/1018240805/filter-HIV-1%20Subtype%20A%20(A1)) | [(A1)](http://dbpartners.stanford.edu:8080/RegaSubtyping/stanford-hiv/typingtool/job/1018240805/filter-HIV-1%20Subtype%20A%20(A1)) | [(A1)](http://dbpartners.stanford.edu:8080/RegaSubtyping/stanford-hiv/typingtool/job/1018240805/filter-HIV-1%20Subtype%20A%20(A1)) | A1 | AD | [(A1)](http://dbpartners.stanford.edu:8080/RegaSubtyping/stanford-hiv/typingtool/job/1018240805/filter-HIV-1%20Subtype%20A%20(A1))  Check for 35-AD | [(A1)](http://dbpartners.stanford.edu:8080/RegaSubtyping/stanford-hiv/typingtool/job/1018240805/filter-HIV-1%20Subtype%20A%20(A1)) | A1 (6), 35-AD (2) |
| **19** | A | (A1) | CRF01 | (A1) | A1 | AD | [(A1)](http://dbpartners.stanford.edu:8080/RegaSubtyping/stanford-hiv/typingtool/job/1018240805/filter-HIV-1%20Subtype%20A%20(A1))  Check for 35-AD | [(A1)](http://dbpartners.stanford.edu:8080/RegaSubtyping/stanford-hiv/typingtool/job/1018240805/filter-HIV-1%20Subtype%20A%20(A1)) | A1 (5), A (1), CRF01 (1), CRF69-01B (1), BF (1) |
| **20** | 35_AD | [(A1)](http://dbpartners.stanford.edu:8080/RegaSubtyping/stanford-hiv/typingtool/job/1018240805/filter-HIV-1%20Subtype%20A%20(A1)) | [(A1)](http://dbpartners.stanford.edu:8080/RegaSubtyping/stanford-hiv/typingtool/job/1018240805/filter-HIV-1%20Subtype%20A%20(A1)) | [(A1)](http://dbpartners.stanford.edu:8080/RegaSubtyping/stanford-hiv/typingtool/job/1018240805/filter-HIV-1%20Subtype%20A%20(A1)) | A1 | AD | [(A1)](http://dbpartners.stanford.edu:8080/RegaSubtyping/stanford-hiv/typingtool/job/1018240805/filter-HIV-1%20Subtype%20A%20(A1))  Check for 35-AD | [(A1)](http://dbpartners.stanford.edu:8080/RegaSubtyping/stanford-hiv/typingtool/job/1018240805/filter-HIV-1%20Subtype%20A%20(A1)) | A1 (6), 35-AD (2) |
| **21** | 35_AD | [(A1)](http://dbpartners.stanford.edu:8080/RegaSubtyping/stanford-hiv/typingtool/job/1018240805/filter-HIV-1%20Subtype%20A%20(A1)) | [(A1)](http://dbpartners.stanford.edu:8080/RegaSubtyping/stanford-hiv/typingtool/job/1018240805/filter-HIV-1%20Subtype%20A%20(A1)) | [(A1)](http://dbpartners.stanford.edu:8080/RegaSubtyping/stanford-hiv/typingtool/job/1018240805/filter-HIV-1%20Subtype%20A%20(A1)) | A1 | AD | [(A1)](http://dbpartners.stanford.edu:8080/RegaSubtyping/stanford-hiv/typingtool/job/1018240805/filter-HIV-1%20Subtype%20A%20(A1))  Check for 35-AD | [(A1)](http://dbpartners.stanford.edu:8080/RegaSubtyping/stanford-hiv/typingtool/job/1018240805/filter-HIV-1%20Subtype%20A%20(A1)) | A1 (6), 35-AD (2) |
| **22** | 35_AD | [(A1)](http://dbpartners.stanford.edu:8080/RegaSubtyping/stanford-hiv/typingtool/job/1018240805/filter-HIV-1%20Subtype%20A%20(A1)) | [(A1)](http://dbpartners.stanford.edu:8080/RegaSubtyping/stanford-hiv/typingtool/job/1018240805/filter-HIV-1%20Subtype%20A%20(A1)) | [(A1)](http://dbpartners.stanford.edu:8080/RegaSubtyping/stanford-hiv/typingtool/job/1018240805/filter-HIV-1%20Subtype%20A%20(A1)) | A1 | BF | [(A1)](http://dbpartners.stanford.edu:8080/RegaSubtyping/stanford-hiv/typingtool/job/1018240805/filter-HIV-1%20Subtype%20A%20(A1)) | [(A1)](http://dbpartners.stanford.edu:8080/RegaSubtyping/stanford-hiv/typingtool/job/1018240805/filter-HIV-1%20Subtype%20A%20(A1)) | A1 (6), 35-AD (1), CRF69-01B (1), BF (1) |
| **23** | 35_AD | [(A1)](http://dbpartners.stanford.edu:8080/RegaSubtyping/stanford-hiv/typingtool/job/1018240805/filter-HIV-1%20Subtype%20A%20(A1)) | [(A1)](http://dbpartners.stanford.edu:8080/RegaSubtyping/stanford-hiv/typingtool/job/1018240805/filter-HIV-1%20Subtype%20A%20(A1)) | [(A1)](http://dbpartners.stanford.edu:8080/RegaSubtyping/stanford-hiv/typingtool/job/1018240805/filter-HIV-1%20Subtype%20A%20(A1)) | A1 | AD | [(A1)](http://dbpartners.stanford.edu:8080/RegaSubtyping/stanford-hiv/typingtool/job/1018240805/filter-HIV-1%20Subtype%20A%20(A1))  Check for 35-AD | [(A1)](http://dbpartners.stanford.edu:8080/RegaSubtyping/stanford-hiv/typingtool/job/1018240805/filter-HIV-1%20Subtype%20A%20(A1)) | A1 (6), 35-AD (2) |
| **24** | 35_AD | [(A1)](http://dbpartners.stanford.edu:8080/RegaSubtyping/stanford-hiv/typingtool/job/1018240805/filter-HIV-1%20Subtype%20A%20(A1)) | [(A1)](http://dbpartners.stanford.edu:8080/RegaSubtyping/stanford-hiv/typingtool/job/1018240805/filter-HIV-1%20Subtype%20A%20(A1)) | [(A1)](http://dbpartners.stanford.edu:8080/RegaSubtyping/stanford-hiv/typingtool/job/1018240805/filter-HIV-1%20Subtype%20A%20(A1)) | A1 | AD | [(A1)](http://dbpartners.stanford.edu:8080/RegaSubtyping/stanford-hiv/typingtool/job/1018240805/filter-HIV-1%20Subtype%20A%20(A1))  Check for 35-AD | [(A1)](http://dbpartners.stanford.edu:8080/RegaSubtyping/stanford-hiv/typingtool/job/1018240805/filter-HIV-1%20Subtype%20A%20(A1)) | A1 (6), 35-AD (2) |
| **25** | 35_AD | [(A1)](http://dbpartners.stanford.edu:8080/RegaSubtyping/stanford-hiv/typingtool/job/1018240805/filter-HIV-1%20Subtype%20A%20(A1)) | [(A1)](http://dbpartners.stanford.edu:8080/RegaSubtyping/stanford-hiv/typingtool/job/1018240805/filter-HIV-1%20Subtype%20A%20(A1)) | [(A1)](http://dbpartners.stanford.edu:8080/RegaSubtyping/stanford-hiv/typingtool/job/1018240805/filter-HIV-1%20Subtype%20A%20(A1)) | A1 | AD | [(A1)](http://dbpartners.stanford.edu:8080/RegaSubtyping/stanford-hiv/typingtool/job/1018240805/filter-HIV-1%20Subtype%20A%20(A1))  Check for 35-AD | [(A1)](http://dbpartners.stanford.edu:8080/RegaSubtyping/stanford-hiv/typingtool/job/1018240805/filter-HIV-1%20Subtype%20A%20(A1)) | A1 (6), 35-AD (2) |
| **26** | 35_AD | [(A1)](http://dbpartners.stanford.edu:8080/RegaSubtyping/stanford-hiv/typingtool/job/1018240805/filter-HIV-1%20Subtype%20A%20(A1)) | [(A1)](http://dbpartners.stanford.edu:8080/RegaSubtyping/stanford-hiv/typingtool/job/1018240805/filter-HIV-1%20Subtype%20A%20(A1)) | [(A1)](http://dbpartners.stanford.edu:8080/RegaSubtyping/stanford-hiv/typingtool/job/1018240805/filter-HIV-1%20Subtype%20A%20(A1)) | A1 | AD | [(A1)](http://dbpartners.stanford.edu:8080/RegaSubtyping/stanford-hiv/typingtool/job/1018240805/filter-HIV-1%20Subtype%20A%20(A1))  Check for 35-AD | [(A1)](http://dbpartners.stanford.edu:8080/RegaSubtyping/stanford-hiv/typingtool/job/1018240805/filter-HIV-1%20Subtype%20A%20(A1)) | A1 (6), 35-AD (2) |
| **27** | 35_AD | [(A1)](http://dbpartners.stanford.edu:8080/RegaSubtyping/stanford-hiv/typingtool/job/1018240805/filter-HIV-1%20Subtype%20A%20(A1)) | [(A1)](http://dbpartners.stanford.edu:8080/RegaSubtyping/stanford-hiv/typingtool/job/1018240805/filter-HIV-1%20Subtype%20A%20(A1)) | [(A1)](http://dbpartners.stanford.edu:8080/RegaSubtyping/stanford-hiv/typingtool/job/1018240805/filter-HIV-1%20Subtype%20A%20(A1)) | A1 | AD | [(A1)](http://dbpartners.stanford.edu:8080/RegaSubtyping/stanford-hiv/typingtool/job/1018240805/filter-HIV-1%20Subtype%20A%20(A1))  Check for 35-AD | [(A1)](http://dbpartners.stanford.edu:8080/RegaSubtyping/stanford-hiv/typingtool/job/1018240805/filter-HIV-1%20Subtype%20A%20(A1)) | A1 (6), 35-AD (1), CRF69-01B (1), BF (1) |
| **28** | 35_AD | [(A1)](http://dbpartners.stanford.edu:8080/RegaSubtyping/stanford-hiv/typingtool/job/1018240805/filter-HIV-1%20Subtype%20A%20(A1)) | [(A1)](http://dbpartners.stanford.edu:8080/RegaSubtyping/stanford-hiv/typingtool/job/1018240805/filter-HIV-1%20Subtype%20A%20(A1)) | [(A1)](http://dbpartners.stanford.edu:8080/RegaSubtyping/stanford-hiv/typingtool/job/1018240805/filter-HIV-1%20Subtype%20A%20(A1)) | A1 | AD | [(A1)](http://dbpartners.stanford.edu:8080/RegaSubtyping/stanford-hiv/typingtool/job/1018240805/filter-HIV-1%20Subtype%20A%20(A1))  Check for 35-AD | [(A1)](http://dbpartners.stanford.edu:8080/RegaSubtyping/stanford-hiv/typingtool/job/1018240805/filter-HIV-1%20Subtype%20A%20(A1)) | A1 (6), 35-AD (2) |
| **29** | 35_AD | [(A1)](http://dbpartners.stanford.edu:8080/RegaSubtyping/stanford-hiv/typingtool/job/1018240805/filter-HIV-1%20Subtype%20A%20(A1)) | [(A1)](http://dbpartners.stanford.edu:8080/RegaSubtyping/stanford-hiv/typingtool/job/1018240805/filter-HIV-1%20Subtype%20A%20(A1)) | [(A1)](http://dbpartners.stanford.edu:8080/RegaSubtyping/stanford-hiv/typingtool/job/1018240805/filter-HIV-1%20Subtype%20A%20(A1)) | A1 | AD | [(A1)](http://dbpartners.stanford.edu:8080/RegaSubtyping/stanford-hiv/typingtool/job/1018240805/filter-HIV-1%20Subtype%20A%20(A1))  Check for 35-AD | [(A1)](http://dbpartners.stanford.edu:8080/RegaSubtyping/stanford-hiv/typingtool/job/1018240805/filter-HIV-1%20Subtype%20A%20(A1)) | A1 (6), 35-AD (2) |
| **30** | 35_AD | [(A1)](http://dbpartners.stanford.edu:8080/RegaSubtyping/stanford-hiv/typingtool/job/1018240805/filter-HIV-1%20Subtype%20A%20(A1)) | [(A1)](http://dbpartners.stanford.edu:8080/RegaSubtyping/stanford-hiv/typingtool/job/1018240805/filter-HIV-1%20Subtype%20A%20(A1)) | [(A1)](http://dbpartners.stanford.edu:8080/RegaSubtyping/stanford-hiv/typingtool/job/1018240805/filter-HIV-1%20Subtype%20A%20(A1)) | A1 | AD | [(A1)](http://dbpartners.stanford.edu:8080/RegaSubtyping/stanford-hiv/typingtool/job/1018240805/filter-HIV-1%20Subtype%20A%20(A1))  Check for 35-AD | [(A1)](http://dbpartners.stanford.edu:8080/RegaSubtyping/stanford-hiv/typingtool/job/1018240805/filter-HIV-1%20Subtype%20A%20(A1)) | A1 (6), 35-AD (2) |
| **31** | 35_AD | [(A1)](http://dbpartners.stanford.edu:8080/RegaSubtyping/stanford-hiv/typingtool/job/1018240805/filter-HIV-1%20Subtype%20A%20(A1)) | [(A1)](http://dbpartners.stanford.edu:8080/RegaSubtyping/stanford-hiv/typingtool/job/1018240805/filter-HIV-1%20Subtype%20A%20(A1)) | [(A1)](http://dbpartners.stanford.edu:8080/RegaSubtyping/stanford-hiv/typingtool/job/1018240805/filter-HIV-1%20Subtype%20A%20(A1)) | A1 | AD | [(A1)](http://dbpartners.stanford.edu:8080/RegaSubtyping/stanford-hiv/typingtool/job/1018240805/filter-HIV-1%20Subtype%20A%20(A1))  Check for 35-AD | [(A1)](http://dbpartners.stanford.edu:8080/RegaSubtyping/stanford-hiv/typingtool/job/1018240805/filter-HIV-1%20Subtype%20A%20(A1)) | A1 (6), 35-AD (2) |
| **32** | 35_AD | NA | [(A1)](http://dbpartners.stanford.edu:8080/RegaSubtyping/stanford-hiv/typingtool/job/1018240805/filter-HIV-1%20Subtype%20A%20(A1)) | [(A1)](http://dbpartners.stanford.edu:8080/RegaSubtyping/stanford-hiv/typingtool/job/1018240805/filter-HIV-1%20Subtype%20A%20(A1)) | A1 | AD | [(A1)](http://dbpartners.stanford.edu:8080/RegaSubtyping/stanford-hiv/typingtool/job/1018240805/filter-HIV-1%20Subtype%20A%20(A1))  Check for 35-AD | [(A1)](http://dbpartners.stanford.edu:8080/RegaSubtyping/stanford-hiv/typingtool/job/1018240805/filter-HIV-1%20Subtype%20A%20(A1)) | A1 (5), 35-AD (2), NA (1) |
| **33** | 35_AD | [(A1)](http://dbpartners.stanford.edu:8080/RegaSubtyping/stanford-hiv/typingtool/job/1018240805/filter-HIV-1%20Subtype%20A%20(A1)) | [(A1)](http://dbpartners.stanford.edu:8080/RegaSubtyping/stanford-hiv/typingtool/job/1018240805/filter-HIV-1%20Subtype%20A%20(A1)) | [(A1)](http://dbpartners.stanford.edu:8080/RegaSubtyping/stanford-hiv/typingtool/job/1018240805/filter-HIV-1%20Subtype%20A%20(A1)) | A1 | AD | [(A1)](http://dbpartners.stanford.edu:8080/RegaSubtyping/stanford-hiv/typingtool/job/1018240805/filter-HIV-1%20Subtype%20A%20(A1))  Check for 35-AD | [(A1)](http://dbpartners.stanford.edu:8080/RegaSubtyping/stanford-hiv/typingtool/job/1018240805/filter-HIV-1%20Subtype%20A%20(A1)) | A1 (6), 35-AD (2) |
| **34** | 35_AD | [(A1)](http://dbpartners.stanford.edu:8080/RegaSubtyping/stanford-hiv/typingtool/job/1018240805/filter-HIV-1%20Subtype%20A%20(A1)) | [(A1)](http://dbpartners.stanford.edu:8080/RegaSubtyping/stanford-hiv/typingtool/job/1018240805/filter-HIV-1%20Subtype%20A%20(A1)) | [(A1)](http://dbpartners.stanford.edu:8080/RegaSubtyping/stanford-hiv/typingtool/job/1018240805/filter-HIV-1%20Subtype%20A%20(A1)) | A1 | AD | [(A1)](http://dbpartners.stanford.edu:8080/RegaSubtyping/stanford-hiv/typingtool/job/1018240805/filter-HIV-1%20Subtype%20A%20(A1))  Check for 35-AD | [(A1)](http://dbpartners.stanford.edu:8080/RegaSubtyping/stanford-hiv/typingtool/job/1018240805/filter-HIV-1%20Subtype%20A%20(A1)) | A1 (6), 35-AD (2) |
| **35** | 35_AD | [(A1)](http://dbpartners.stanford.edu:8080/RegaSubtyping/stanford-hiv/typingtool/job/1018240805/filter-HIV-1%20Subtype%20A%20(A1)) | [(A1)](http://dbpartners.stanford.edu:8080/RegaSubtyping/stanford-hiv/typingtool/job/1018240805/filter-HIV-1%20Subtype%20A%20(A1)) | [(A1)](http://dbpartners.stanford.edu:8080/RegaSubtyping/stanford-hiv/typingtool/job/1018240805/filter-HIV-1%20Subtype%20A%20(A1)) | A1 | AD | [(A1)](http://dbpartners.stanford.edu:8080/RegaSubtyping/stanford-hiv/typingtool/job/1018240805/filter-HIV-1%20Subtype%20A%20(A1))  Check for 35-AD | [(A1)](http://dbpartners.stanford.edu:8080/RegaSubtyping/stanford-hiv/typingtool/job/1018240805/filter-HIV-1%20Subtype%20A%20(A1)) | A1 (6), 35-AD (2) |
| **36** | 35_AD | [(A1)](http://dbpartners.stanford.edu:8080/RegaSubtyping/stanford-hiv/typingtool/job/1018240805/filter-HIV-1%20Subtype%20A%20(A1)) | [(A1)](http://dbpartners.stanford.edu:8080/RegaSubtyping/stanford-hiv/typingtool/job/1018240805/filter-HIV-1%20Subtype%20A%20(A1)) | [(A1)](http://dbpartners.stanford.edu:8080/RegaSubtyping/stanford-hiv/typingtool/job/1018240805/filter-HIV-1%20Subtype%20A%20(A1)) | A1 | AD | [(A1)](http://dbpartners.stanford.edu:8080/RegaSubtyping/stanford-hiv/typingtool/job/1018240805/filter-HIV-1%20Subtype%20A%20(A1))  Check for 35-AD | [(A1)](http://dbpartners.stanford.edu:8080/RegaSubtyping/stanford-hiv/typingtool/job/1018240805/filter-HIV-1%20Subtype%20A%20(A1)) | A1 (6), 35-AD (2) |
| **37** | 35_AD | [(A1)](http://dbpartners.stanford.edu:8080/RegaSubtyping/stanford-hiv/typingtool/job/1018240805/filter-HIV-1%20Subtype%20A%20(A1)) | [(A1)](http://dbpartners.stanford.edu:8080/RegaSubtyping/stanford-hiv/typingtool/job/1018240805/filter-HIV-1%20Subtype%20A%20(A1)) | [(A1)](http://dbpartners.stanford.edu:8080/RegaSubtyping/stanford-hiv/typingtool/job/1018240805/filter-HIV-1%20Subtype%20A%20(A1)) | A1 | AD | [(A1)](http://dbpartners.stanford.edu:8080/RegaSubtyping/stanford-hiv/typingtool/job/1018240805/filter-HIV-1%20Subtype%20A%20(A1))  Check for 35-AD | [(A1)](http://dbpartners.stanford.edu:8080/RegaSubtyping/stanford-hiv/typingtool/job/1018240805/filter-HIV-1%20Subtype%20A%20(A1)) | A1 (6), 35-AD (2) |
| **38** | 35_AD | [(A1)](http://dbpartners.stanford.edu:8080/RegaSubtyping/stanford-hiv/typingtool/job/1018240805/filter-HIV-1%20Subtype%20A%20(A1)) | [(A1)](http://dbpartners.stanford.edu:8080/RegaSubtyping/stanford-hiv/typingtool/job/1018240805/filter-HIV-1%20Subtype%20A%20(A1)) | [(A1)](http://dbpartners.stanford.edu:8080/RegaSubtyping/stanford-hiv/typingtool/job/1018240805/filter-HIV-1%20Subtype%20A%20(A1)) | A1 | AD | [(A1)](http://dbpartners.stanford.edu:8080/RegaSubtyping/stanford-hiv/typingtool/job/1018240805/filter-HIV-1%20Subtype%20A%20(A1))  Check for 35-AD | [(A1)](http://dbpartners.stanford.edu:8080/RegaSubtyping/stanford-hiv/typingtool/job/1018240805/filter-HIV-1%20Subtype%20A%20(A1)) | A1 (6), 35-AD (2) |
| **39** | 35_AD | [(A1)](http://dbpartners.stanford.edu:8080/RegaSubtyping/stanford-hiv/typingtool/job/1018240805/filter-HIV-1%20Subtype%20A%20(A1)) | [(A1)](http://dbpartners.stanford.edu:8080/RegaSubtyping/stanford-hiv/typingtool/job/1018240805/filter-HIV-1%20Subtype%20A%20(A1)) | [(A1)](http://dbpartners.stanford.edu:8080/RegaSubtyping/stanford-hiv/typingtool/job/1018240805/filter-HIV-1%20Subtype%20A%20(A1)) | A1 | AD | [(A1)](http://dbpartners.stanford.edu:8080/RegaSubtyping/stanford-hiv/typingtool/job/1018240805/filter-HIV-1%20Subtype%20A%20(A1))  Check for 35-AD | [(A1)](http://dbpartners.stanford.edu:8080/RegaSubtyping/stanford-hiv/typingtool/job/1018240805/filter-HIV-1%20Subtype%20A%20(A1)) | A1 (6), 35-AD (2) |
| **40** | 35_AD | [(A1)](http://dbpartners.stanford.edu:8080/RegaSubtyping/stanford-hiv/typingtool/job/1018240805/filter-HIV-1%20Subtype%20A%20(A1)) | [(A1)](http://dbpartners.stanford.edu:8080/RegaSubtyping/stanford-hiv/typingtool/job/1018240805/filter-HIV-1%20Subtype%20A%20(A1)) | [(A1)](http://dbpartners.stanford.edu:8080/RegaSubtyping/stanford-hiv/typingtool/job/1018240805/filter-HIV-1%20Subtype%20A%20(A1)) | A1 | AD | [(A1)](http://dbpartners.stanford.edu:8080/RegaSubtyping/stanford-hiv/typingtool/job/1018240805/filter-HIV-1%20Subtype%20A%20(A1))  Check for 35-AD | [(A1)](http://dbpartners.stanford.edu:8080/RegaSubtyping/stanford-hiv/typingtool/job/1018240805/filter-HIV-1%20Subtype%20A%20(A1)) | A1 (6), 35-AD (2) |
| **41** | 35_AD | [(A1)](http://dbpartners.stanford.edu:8080/RegaSubtyping/stanford-hiv/typingtool/job/1018240805/filter-HIV-1%20Subtype%20A%20(A1)) | [(A1)](http://dbpartners.stanford.edu:8080/RegaSubtyping/stanford-hiv/typingtool/job/1018240805/filter-HIV-1%20Subtype%20A%20(A1)) | [(A1)](http://dbpartners.stanford.edu:8080/RegaSubtyping/stanford-hiv/typingtool/job/1018240805/filter-HIV-1%20Subtype%20A%20(A1)) | A1 | AD | [(A1)](http://dbpartners.stanford.edu:8080/RegaSubtyping/stanford-hiv/typingtool/job/1018240805/filter-HIV-1%20Subtype%20A%20(A1))  Check for 35-AD | [(A1)](http://dbpartners.stanford.edu:8080/RegaSubtyping/stanford-hiv/typingtool/job/1018240805/filter-HIV-1%20Subtype%20A%20(A1)) | A1 (6), 35-AD (2) |
| **42** | 35_AD | [(A1)](http://dbpartners.stanford.edu:8080/RegaSubtyping/stanford-hiv/typingtool/job/1018240805/filter-HIV-1%20Subtype%20A%20(A1)) | [(A1)](http://dbpartners.stanford.edu:8080/RegaSubtyping/stanford-hiv/typingtool/job/1018240805/filter-HIV-1%20Subtype%20A%20(A1)) | [(A1)](http://dbpartners.stanford.edu:8080/RegaSubtyping/stanford-hiv/typingtool/job/1018240805/filter-HIV-1%20Subtype%20A%20(A1)) | A1 | AD | [(A1)](http://dbpartners.stanford.edu:8080/RegaSubtyping/stanford-hiv/typingtool/job/1018240805/filter-HIV-1%20Subtype%20A%20(A1))  Check for 35-AD | [(A1)](http://dbpartners.stanford.edu:8080/RegaSubtyping/stanford-hiv/typingtool/job/1018240805/filter-HIV-1%20Subtype%20A%20(A1)) | A1 (6), 35-AD (2) |
| **43** | 35_AD | [(A1)](http://dbpartners.stanford.edu:8080/RegaSubtyping/stanford-hiv/typingtool/job/1018240805/filter-HIV-1%20Subtype%20A%20(A1)) | [(A1)](http://dbpartners.stanford.edu:8080/RegaSubtyping/stanford-hiv/typingtool/job/1018240805/filter-HIV-1%20Subtype%20A%20(A1)) | [(A1)](http://dbpartners.stanford.edu:8080/RegaSubtyping/stanford-hiv/typingtool/job/1018240805/filter-HIV-1%20Subtype%20A%20(A1)) | A1 | AD | [(A1)](http://dbpartners.stanford.edu:8080/RegaSubtyping/stanford-hiv/typingtool/job/1018240805/filter-HIV-1%20Subtype%20A%20(A1))  Check for 35-AD | [(A1)](http://dbpartners.stanford.edu:8080/RegaSubtyping/stanford-hiv/typingtool/job/1018240805/filter-HIV-1%20Subtype%20A%20(A1)) | A1 (6), 35-AD (2) |
| **44** | 35_AD | [(A1)](http://dbpartners.stanford.edu:8080/RegaSubtyping/stanford-hiv/typingtool/job/1018240805/filter-HIV-1%20Subtype%20A%20(A1)) | [(A1)](http://dbpartners.stanford.edu:8080/RegaSubtyping/stanford-hiv/typingtool/job/1018240805/filter-HIV-1%20Subtype%20A%20(A1)) | [(A1)](http://dbpartners.stanford.edu:8080/RegaSubtyping/stanford-hiv/typingtool/job/1018240805/filter-HIV-1%20Subtype%20A%20(A1)) | A1 | AD | [(A1)](http://dbpartners.stanford.edu:8080/RegaSubtyping/stanford-hiv/typingtool/job/1018240805/filter-HIV-1%20Subtype%20A%20(A1))  Check for 35-AD | [(A1)](http://dbpartners.stanford.edu:8080/RegaSubtyping/stanford-hiv/typingtool/job/1018240805/filter-HIV-1%20Subtype%20A%20(A1)) | A1 (6), 35-AD (2) |
| **45** | 35_AD | [(A1)](http://dbpartners.stanford.edu:8080/RegaSubtyping/stanford-hiv/typingtool/job/1018240805/filter-HIV-1%20Subtype%20A%20(A1)) | [(A1)](http://dbpartners.stanford.edu:8080/RegaSubtyping/stanford-hiv/typingtool/job/1018240805/filter-HIV-1%20Subtype%20A%20(A1)) | [(A1)](http://dbpartners.stanford.edu:8080/RegaSubtyping/stanford-hiv/typingtool/job/1018240805/filter-HIV-1%20Subtype%20A%20(A1)) | A1 | AD | [(A1)](http://dbpartners.stanford.edu:8080/RegaSubtyping/stanford-hiv/typingtool/job/1018240805/filter-HIV-1%20Subtype%20A%20(A1))  Check for 35-AD | [(A1)](http://dbpartners.stanford.edu:8080/RegaSubtyping/stanford-hiv/typingtool/job/1018240805/filter-HIV-1%20Subtype%20A%20(A1)) | A1 (6), 35-AD (2) |
| **46** | 35_AD | [(A1)](http://dbpartners.stanford.edu:8080/RegaSubtyping/stanford-hiv/typingtool/job/1018240805/filter-HIV-1%20Subtype%20A%20(A1)) | [(A1)](http://dbpartners.stanford.edu:8080/RegaSubtyping/stanford-hiv/typingtool/job/1018240805/filter-HIV-1%20Subtype%20A%20(A1)) | [(A1)](http://dbpartners.stanford.edu:8080/RegaSubtyping/stanford-hiv/typingtool/job/1018240805/filter-HIV-1%20Subtype%20A%20(A1)) | A1 | AD | [(A1)](http://dbpartners.stanford.edu:8080/RegaSubtyping/stanford-hiv/typingtool/job/1018240805/filter-HIV-1%20Subtype%20A%20(A1))  Check for 35-AD | [(A1)](http://dbpartners.stanford.edu:8080/RegaSubtyping/stanford-hiv/typingtool/job/1018240805/filter-HIV-1%20Subtype%20A%20(A1)) | A1 (6), 35-AD (2) |
| **47** | 35_AD | [(A1)](http://dbpartners.stanford.edu:8080/RegaSubtyping/stanford-hiv/typingtool/job/1018240805/filter-HIV-1%20Subtype%20A%20(A1)) | [(A1)](http://dbpartners.stanford.edu:8080/RegaSubtyping/stanford-hiv/typingtool/job/1018240805/filter-HIV-1%20Subtype%20A%20(A1)) | [(A1)](http://dbpartners.stanford.edu:8080/RegaSubtyping/stanford-hiv/typingtool/job/1018240805/filter-HIV-1%20Subtype%20A%20(A1)) | A1 | AD | [(A1)](http://dbpartners.stanford.edu:8080/RegaSubtyping/stanford-hiv/typingtool/job/1018240805/filter-HIV-1%20Subtype%20A%20(A1))  Check for 35-AD | [(A1)](http://dbpartners.stanford.edu:8080/RegaSubtyping/stanford-hiv/typingtool/job/1018240805/filter-HIV-1%20Subtype%20A%20(A1)) | A1 (6), 35-AD (2) |
| **48** | 35_AD | [(A1)](http://dbpartners.stanford.edu:8080/RegaSubtyping/stanford-hiv/typingtool/job/1018240805/filter-HIV-1%20Subtype%20A%20(A1)) | [(A1)](http://dbpartners.stanford.edu:8080/RegaSubtyping/stanford-hiv/typingtool/job/1018240805/filter-HIV-1%20Subtype%20A%20(A1)) | [(A1)](http://dbpartners.stanford.edu:8080/RegaSubtyping/stanford-hiv/typingtool/job/1018240805/filter-HIV-1%20Subtype%20A%20(A1)) | A1 | AD | [(A1)](http://dbpartners.stanford.edu:8080/RegaSubtyping/stanford-hiv/typingtool/job/1018240805/filter-HIV-1%20Subtype%20A%20(A1))  Check for 35-AD | [(A1)](http://dbpartners.stanford.edu:8080/RegaSubtyping/stanford-hiv/typingtool/job/1018240805/filter-HIV-1%20Subtype%20A%20(A1)) | A1 (6), 35-AD (1), CRF69-01B (1), BF (1) |
| **49** | 35_AD | [(A1)](http://dbpartners.stanford.edu:8080/RegaSubtyping/stanford-hiv/typingtool/job/1018240805/filter-HIV-1%20Subtype%20A%20(A1)) | [(A1)](http://dbpartners.stanford.edu:8080/RegaSubtyping/stanford-hiv/typingtool/job/1018240805/filter-HIV-1%20Subtype%20A%20(A1)) | [(A1)](http://dbpartners.stanford.edu:8080/RegaSubtyping/stanford-hiv/typingtool/job/1018240805/filter-HIV-1%20Subtype%20A%20(A1)) | A1 | AD | [(A1)](http://dbpartners.stanford.edu:8080/RegaSubtyping/stanford-hiv/typingtool/job/1018240805/filter-HIV-1%20Subtype%20A%20(A1))  Check for 35-AD | [(A1)](http://dbpartners.stanford.edu:8080/RegaSubtyping/stanford-hiv/typingtool/job/1018240805/filter-HIV-1%20Subtype%20A%20(A1)) | A1 (6), 35-AD (2) |
| **50** | 35_AD | [(A1)](http://dbpartners.stanford.edu:8080/RegaSubtyping/stanford-hiv/typingtool/job/1018240805/filter-HIV-1%20Subtype%20A%20(A1)) | [(A1)](http://dbpartners.stanford.edu:8080/RegaSubtyping/stanford-hiv/typingtool/job/1018240805/filter-HIV-1%20Subtype%20A%20(A1)) | [(A1)](http://dbpartners.stanford.edu:8080/RegaSubtyping/stanford-hiv/typingtool/job/1018240805/filter-HIV-1%20Subtype%20A%20(A1)) | A1 | AD | [(A1)](http://dbpartners.stanford.edu:8080/RegaSubtyping/stanford-hiv/typingtool/job/1018240805/filter-HIV-1%20Subtype%20A%20(A1))  Check for 35-AD | [(A1)](http://dbpartners.stanford.edu:8080/RegaSubtyping/stanford-hiv/typingtool/job/1018240805/filter-HIV-1%20Subtype%20A%20(A1)) | A1 (6), 35-AD (2) |
| **51** | 35_AD | [(A1)](http://dbpartners.stanford.edu:8080/RegaSubtyping/stanford-hiv/typingtool/job/1018240805/filter-HIV-1%20Subtype%20A%20(A1)) | [(A1)](http://dbpartners.stanford.edu:8080/RegaSubtyping/stanford-hiv/typingtool/job/1018240805/filter-HIV-1%20Subtype%20A%20(A1)) | [(A1)](http://dbpartners.stanford.edu:8080/RegaSubtyping/stanford-hiv/typingtool/job/1018240805/filter-HIV-1%20Subtype%20A%20(A1)) | A1 | AD | [(A1)](http://dbpartners.stanford.edu:8080/RegaSubtyping/stanford-hiv/typingtool/job/1018240805/filter-HIV-1%20Subtype%20A%20(A1))  Check for 35-AD | [(A1)](http://dbpartners.stanford.edu:8080/RegaSubtyping/stanford-hiv/typingtool/job/1018240805/filter-HIV-1%20Subtype%20A%20(A1)) | A1 (6), 35-AD (2) |
| **52** | 35_AD | [(A1)](http://dbpartners.stanford.edu:8080/RegaSubtyping/stanford-hiv/typingtool/job/1018240805/filter-HIV-1%20Subtype%20A%20(A1)) | [(A1)](http://dbpartners.stanford.edu:8080/RegaSubtyping/stanford-hiv/typingtool/job/1018240805/filter-HIV-1%20Subtype%20A%20(A1)) | [(A1)](http://dbpartners.stanford.edu:8080/RegaSubtyping/stanford-hiv/typingtool/job/1018240805/filter-HIV-1%20Subtype%20A%20(A1)) | A1 | AD | [(A1)](http://dbpartners.stanford.edu:8080/RegaSubtyping/stanford-hiv/typingtool/job/1018240805/filter-HIV-1%20Subtype%20A%20(A1))  Check for 35-AD | [(A1)](http://dbpartners.stanford.edu:8080/RegaSubtyping/stanford-hiv/typingtool/job/1018240805/filter-HIV-1%20Subtype%20A%20(A1)) | A1 (6), 35-AD (2) |
| **53** | 35_AD | [(A1)](http://dbpartners.stanford.edu:8080/RegaSubtyping/stanford-hiv/typingtool/job/1018240805/filter-HIV-1%20Subtype%20A%20(A1)) | [(A1)](http://dbpartners.stanford.edu:8080/RegaSubtyping/stanford-hiv/typingtool/job/1018240805/filter-HIV-1%20Subtype%20A%20(A1)) | [(A1)](http://dbpartners.stanford.edu:8080/RegaSubtyping/stanford-hiv/typingtool/job/1018240805/filter-HIV-1%20Subtype%20A%20(A1)) | A1 | AD | [(A1)](http://dbpartners.stanford.edu:8080/RegaSubtyping/stanford-hiv/typingtool/job/1018240805/filter-HIV-1%20Subtype%20A%20(A1))  Check for 35-AD | [(A1)](http://dbpartners.stanford.edu:8080/RegaSubtyping/stanford-hiv/typingtool/job/1018240805/filter-HIV-1%20Subtype%20A%20(A1)) | A1 (6), 35-AD (2) |
| **54** | 35_AD | [(A1)](http://dbpartners.stanford.edu:8080/RegaSubtyping/stanford-hiv/typingtool/job/1018240805/filter-HIV-1%20Subtype%20A%20(A1)) | [(A1)](http://dbpartners.stanford.edu:8080/RegaSubtyping/stanford-hiv/typingtool/job/1018240805/filter-HIV-1%20Subtype%20A%20(A1)) | [(A1)](http://dbpartners.stanford.edu:8080/RegaSubtyping/stanford-hiv/typingtool/job/1018240805/filter-HIV-1%20Subtype%20A%20(A1)) | A1 | AD | [(A1)](http://dbpartners.stanford.edu:8080/RegaSubtyping/stanford-hiv/typingtool/job/1018240805/filter-HIV-1%20Subtype%20A%20(A1))  Check for 35-AD | [(A1)](http://dbpartners.stanford.edu:8080/RegaSubtyping/stanford-hiv/typingtool/job/1018240805/filter-HIV-1%20Subtype%20A%20(A1)) | A1 (6), 35-AD (2) |
| **55** | 35_AD | [(A1)](http://dbpartners.stanford.edu:8080/RegaSubtyping/stanford-hiv/typingtool/job/1018240805/filter-HIV-1%20Subtype%20A%20(A1)) | [(A1)](http://dbpartners.stanford.edu:8080/RegaSubtyping/stanford-hiv/typingtool/job/1018240805/filter-HIV-1%20Subtype%20A%20(A1)) | [(A1)](http://dbpartners.stanford.edu:8080/RegaSubtyping/stanford-hiv/typingtool/job/1018240805/filter-HIV-1%20Subtype%20A%20(A1)) | A1 | AD | [(A1)](http://dbpartners.stanford.edu:8080/RegaSubtyping/stanford-hiv/typingtool/job/1018240805/filter-HIV-1%20Subtype%20A%20(A1))  Check for 35-AD | [(A1)](http://dbpartners.stanford.edu:8080/RegaSubtyping/stanford-hiv/typingtool/job/1018240805/filter-HIV-1%20Subtype%20A%20(A1)) | A1 (6), 35-AD (2) |
| **56** | 35_AD | [(A1)](http://dbpartners.stanford.edu:8080/RegaSubtyping/stanford-hiv/typingtool/job/1018240805/filter-HIV-1%20Subtype%20A%20(A1)) | [(A1)](http://dbpartners.stanford.edu:8080/RegaSubtyping/stanford-hiv/typingtool/job/1018240805/filter-HIV-1%20Subtype%20A%20(A1)) | [(A1)](http://dbpartners.stanford.edu:8080/RegaSubtyping/stanford-hiv/typingtool/job/1018240805/filter-HIV-1%20Subtype%20A%20(A1)) | A1 | AD | [(A1)](http://dbpartners.stanford.edu:8080/RegaSubtyping/stanford-hiv/typingtool/job/1018240805/filter-HIV-1%20Subtype%20A%20(A1))  Check for 35-AD | [(A1)](http://dbpartners.stanford.edu:8080/RegaSubtyping/stanford-hiv/typingtool/job/1018240805/filter-HIV-1%20Subtype%20A%20(A1)) | A1 (6), 35-AD (2) |
| **57** | 35_AD | NA | [(A1)](http://dbpartners.stanford.edu:8080/RegaSubtyping/stanford-hiv/typingtool/job/1018240805/filter-HIV-1%20Subtype%20A%20(A1)) | [(A1)](http://dbpartners.stanford.edu:8080/RegaSubtyping/stanford-hiv/typingtool/job/1018240805/filter-HIV-1%20Subtype%20A%20(A1)) | A1 | CRF-01B | [(A1)](http://dbpartners.stanford.edu:8080/RegaSubtyping/stanford-hiv/typingtool/job/1018240805/filter-HIV-1%20Subtype%20A%20(A1))  Check for 35-AD | [(A1)](http://dbpartners.stanford.edu:8080/RegaSubtyping/stanford-hiv/typingtool/job/1018240805/filter-HIV-1%20Subtype%20A%20(A1)) | A1 (5), 35-AD (1), NA (1), CRF34-01B (1) |
| **58** | 35_AD | [(A1)](http://dbpartners.stanford.edu:8080/RegaSubtyping/stanford-hiv/typingtool/job/1018240805/filter-HIV-1%20Subtype%20A%20(A1)) | [(A1)](http://dbpartners.stanford.edu:8080/RegaSubtyping/stanford-hiv/typingtool/job/1018240805/filter-HIV-1%20Subtype%20A%20(A1)) | [(A1)](http://dbpartners.stanford.edu:8080/RegaSubtyping/stanford-hiv/typingtool/job/1018240805/filter-HIV-1%20Subtype%20A%20(A1)) | A1 | AD | [(A1)](http://dbpartners.stanford.edu:8080/RegaSubtyping/stanford-hiv/typingtool/job/1018240805/filter-HIV-1%20Subtype%20A%20(A1))  Check for 35-AD | [(A1)](http://dbpartners.stanford.edu:8080/RegaSubtyping/stanford-hiv/typingtool/job/1018240805/filter-HIV-1%20Subtype%20A%20(A1)) | A1 (6), 35-AD (2) |
| **59** | 35_AD | [(A1)](http://dbpartners.stanford.edu:8080/RegaSubtyping/stanford-hiv/typingtool/job/1018240805/filter-HIV-1%20Subtype%20A%20(A1)) | [(A1)](http://dbpartners.stanford.edu:8080/RegaSubtyping/stanford-hiv/typingtool/job/1018240805/filter-HIV-1%20Subtype%20A%20(A1)) | [(A1)](http://dbpartners.stanford.edu:8080/RegaSubtyping/stanford-hiv/typingtool/job/1018240805/filter-HIV-1%20Subtype%20A%20(A1)) | A1 | AD | [(A1)](http://dbpartners.stanford.edu:8080/RegaSubtyping/stanford-hiv/typingtool/job/1018240805/filter-HIV-1%20Subtype%20A%20(A1))  Check for 35-AD | [(A1)](http://dbpartners.stanford.edu:8080/RegaSubtyping/stanford-hiv/typingtool/job/1018240805/filter-HIV-1%20Subtype%20A%20(A1)) | A1 (6), 35-AD (2) |
| **60** | 35_AD | [(A1)](http://dbpartners.stanford.edu:8080/RegaSubtyping/stanford-hiv/typingtool/job/1018240805/filter-HIV-1%20Subtype%20A%20(A1)) | [(A1)](http://dbpartners.stanford.edu:8080/RegaSubtyping/stanford-hiv/typingtool/job/1018240805/filter-HIV-1%20Subtype%20A%20(A1)) | [(A1)](http://dbpartners.stanford.edu:8080/RegaSubtyping/stanford-hiv/typingtool/job/1018240805/filter-HIV-1%20Subtype%20A%20(A1)) | A1 | AD | [(A1)](http://dbpartners.stanford.edu:8080/RegaSubtyping/stanford-hiv/typingtool/job/1018240805/filter-HIV-1%20Subtype%20A%20(A1)) | A1, B | A1 (6), 35-AD (2), B (1) |
| **61** | 35_AD | NA | [(A1)](http://dbpartners.stanford.edu:8080/RegaSubtyping/stanford-hiv/typingtool/job/1018240805/filter-HIV-1%20Subtype%20A%20(A1)) | [(A1)](http://dbpartners.stanford.edu:8080/RegaSubtyping/stanford-hiv/typingtool/job/1018240805/filter-HIV-1%20Subtype%20A%20(A1)) | A1 | AD | [(A1)](http://dbpartners.stanford.edu:8080/RegaSubtyping/stanford-hiv/typingtool/job/1018240805/filter-HIV-1%20Subtype%20A%20(A1))  Check for 35-AD | [(A1)](http://dbpartners.stanford.edu:8080/RegaSubtyping/stanford-hiv/typingtool/job/1018240805/filter-HIV-1%20Subtype%20A%20(A1)) | A1 (5), 35-AD (2), NA (1) |
| **62** | 35_AD | [(A1)](http://dbpartners.stanford.edu:8080/RegaSubtyping/stanford-hiv/typingtool/job/1018240805/filter-HIV-1%20Subtype%20A%20(A1)) | [(A1)](http://dbpartners.stanford.edu:8080/RegaSubtyping/stanford-hiv/typingtool/job/1018240805/filter-HIV-1%20Subtype%20A%20(A1)) | [(A1)](http://dbpartners.stanford.edu:8080/RegaSubtyping/stanford-hiv/typingtool/job/1018240805/filter-HIV-1%20Subtype%20A%20(A1)) | A1 | AD | [(A1)](http://dbpartners.stanford.edu:8080/RegaSubtyping/stanford-hiv/typingtool/job/1018240805/filter-HIV-1%20Subtype%20A%20(A1))  Check for 35-AD | [(A1)](http://dbpartners.stanford.edu:8080/RegaSubtyping/stanford-hiv/typingtool/job/1018240805/filter-HIV-1%20Subtype%20A%20(A1)) | A1 (6), 35-AD (2) |
| **63** | 35_AD | [(A1)](http://dbpartners.stanford.edu:8080/RegaSubtyping/stanford-hiv/typingtool/job/1018240805/filter-HIV-1%20Subtype%20A%20(A1)) | [(A1)](http://dbpartners.stanford.edu:8080/RegaSubtyping/stanford-hiv/typingtool/job/1018240805/filter-HIV-1%20Subtype%20A%20(A1)) | [(A1)](http://dbpartners.stanford.edu:8080/RegaSubtyping/stanford-hiv/typingtool/job/1018240805/filter-HIV-1%20Subtype%20A%20(A1)) | A1 | AD | [(A1)](http://dbpartners.stanford.edu:8080/RegaSubtyping/stanford-hiv/typingtool/job/1018240805/filter-HIV-1%20Subtype%20A%20(A1))  Check for 35-AD | [(A1)](http://dbpartners.stanford.edu:8080/RegaSubtyping/stanford-hiv/typingtool/job/1018240805/filter-HIV-1%20Subtype%20A%20(A1)) | A1 (6), 35-AD (2) |
| **64** | 35_AD | [(A1)](http://dbpartners.stanford.edu:8080/RegaSubtyping/stanford-hiv/typingtool/job/1018240805/filter-HIV-1%20Subtype%20A%20(A1)) | [(A1)](http://dbpartners.stanford.edu:8080/RegaSubtyping/stanford-hiv/typingtool/job/1018240805/filter-HIV-1%20Subtype%20A%20(A1)) | [(A1)](http://dbpartners.stanford.edu:8080/RegaSubtyping/stanford-hiv/typingtool/job/1018240805/filter-HIV-1%20Subtype%20A%20(A1)) | A1 | AD | [(A1)](http://dbpartners.stanford.edu:8080/RegaSubtyping/stanford-hiv/typingtool/job/1018240805/filter-HIV-1%20Subtype%20A%20(A1))  Check for 35-AD | [(A1)](http://dbpartners.stanford.edu:8080/RegaSubtyping/stanford-hiv/typingtool/job/1018240805/filter-HIV-1%20Subtype%20A%20(A1)) | A1 (6), 35-AD (2) |
| **65** | 35_AD | NA | [(A1)](http://dbpartners.stanford.edu:8080/RegaSubtyping/stanford-hiv/typingtool/job/1018240805/filter-HIV-1%20Subtype%20A%20(A1)) | [(A1)](http://dbpartners.stanford.edu:8080/RegaSubtyping/stanford-hiv/typingtool/job/1018240805/filter-HIV-1%20Subtype%20A%20(A1)) | A1 | AD | [(A1)](http://dbpartners.stanford.edu:8080/RegaSubtyping/stanford-hiv/typingtool/job/1018240805/filter-HIV-1%20Subtype%20A%20(A1))  Check for 35-AD | [(A1)](http://dbpartners.stanford.edu:8080/RegaSubtyping/stanford-hiv/typingtool/job/1018240805/filter-HIV-1%20Subtype%20A%20(A1)) | A1 (5), 35-AD (2), NA (1) |
| **66** | 35_AD | [(A1)](http://dbpartners.stanford.edu:8080/RegaSubtyping/stanford-hiv/typingtool/job/1018240805/filter-HIV-1%20Subtype%20A%20(A1)) | [(A1)](http://dbpartners.stanford.edu:8080/RegaSubtyping/stanford-hiv/typingtool/job/1018240805/filter-HIV-1%20Subtype%20A%20(A1)) | [(A1)](http://dbpartners.stanford.edu:8080/RegaSubtyping/stanford-hiv/typingtool/job/1018240805/filter-HIV-1%20Subtype%20A%20(A1)) | A1 | AD | [(A1)](http://dbpartners.stanford.edu:8080/RegaSubtyping/stanford-hiv/typingtool/job/1018240805/filter-HIV-1%20Subtype%20A%20(A1))  Check for 35-AD | [(A1)](http://dbpartners.stanford.edu:8080/RegaSubtyping/stanford-hiv/typingtool/job/1018240805/filter-HIV-1%20Subtype%20A%20(A1)) | A1 (6), 35-AD (2) |
| **67** | 35_AD | NA | [(A1)](http://dbpartners.stanford.edu:8080/RegaSubtyping/stanford-hiv/typingtool/job/1018240805/filter-HIV-1%20Subtype%20A%20(A1)) | [(A1)](http://dbpartners.stanford.edu:8080/RegaSubtyping/stanford-hiv/typingtool/job/1018240805/filter-HIV-1%20Subtype%20A%20(A1)) | A1 | AD | [(A1)](http://dbpartners.stanford.edu:8080/RegaSubtyping/stanford-hiv/typingtool/job/1018240805/filter-HIV-1%20Subtype%20A%20(A1))  Check for 35-AD | A1, K | A1 (5), 35-AD (2), NA (1), K (1) |
| **68** | 35_AD | [(A1)](http://dbpartners.stanford.edu:8080/RegaSubtyping/stanford-hiv/typingtool/job/1018240805/filter-HIV-1%20Subtype%20A%20(A1)) | [(A1)](http://dbpartners.stanford.edu:8080/RegaSubtyping/stanford-hiv/typingtool/job/1018240805/filter-HIV-1%20Subtype%20A%20(A1)) | [(A1)](http://dbpartners.stanford.edu:8080/RegaSubtyping/stanford-hiv/typingtool/job/1018240805/filter-HIV-1%20Subtype%20A%20(A1)) | A1 | AD | [(A1)](http://dbpartners.stanford.edu:8080/RegaSubtyping/stanford-hiv/typingtool/job/1018240805/filter-HIV-1%20Subtype%20A%20(A1))  Check for 35-AD | [(A1)](http://dbpartners.stanford.edu:8080/RegaSubtyping/stanford-hiv/typingtool/job/1018240805/filter-HIV-1%20Subtype%20A%20(A1)) | A1 (6), 35-AD (2) |
| **69** | A | NA | CRF01 | [(A1)](http://dbpartners.stanford.edu:8080/RegaSubtyping/stanford-hiv/typingtool/job/1018240805/filter-HIV-1%20Subtype%20A%20(A1)) | A1 | AD | [(A1)](http://dbpartners.stanford.edu:8080/RegaSubtyping/stanford-hiv/typingtool/job/1018240805/filter-HIV-1%20Subtype%20A%20(A1))  Check for 35-AD | [(A1)](http://dbpartners.stanford.edu:8080/RegaSubtyping/stanford-hiv/typingtool/job/1018240805/filter-HIV-1%20Subtype%20A%20(A1)) | A1 (4), A (1), NA (1), CRF69-01B (2), BF (1) |
| **70** | 35_AD | [(A1)](http://dbpartners.stanford.edu:8080/RegaSubtyping/stanford-hiv/typingtool/job/1018240805/filter-HIV-1%20Subtype%20A%20(A1)) | [(A1)](http://dbpartners.stanford.edu:8080/RegaSubtyping/stanford-hiv/typingtool/job/1018240805/filter-HIV-1%20Subtype%20A%20(A1)) | [(A1)](http://dbpartners.stanford.edu:8080/RegaSubtyping/stanford-hiv/typingtool/job/1018240805/filter-HIV-1%20Subtype%20A%20(A1)) | A1 | AD | [(A1)](http://dbpartners.stanford.edu:8080/RegaSubtyping/stanford-hiv/typingtool/job/1018240805/filter-HIV-1%20Subtype%20A%20(A1))  Check for 35-AD | [(A1)](http://dbpartners.stanford.edu:8080/RegaSubtyping/stanford-hiv/typingtool/job/1018240805/filter-HIV-1%20Subtype%20A%20(A1)) | A1 (6), 35-AD (2) |
| **71** | 35_AD | [(A1)](http://dbpartners.stanford.edu:8080/RegaSubtyping/stanford-hiv/typingtool/job/1018240805/filter-HIV-1%20Subtype%20A%20(A1)) | [(A1)](http://dbpartners.stanford.edu:8080/RegaSubtyping/stanford-hiv/typingtool/job/1018240805/filter-HIV-1%20Subtype%20A%20(A1)) | [(A1)](http://dbpartners.stanford.edu:8080/RegaSubtyping/stanford-hiv/typingtool/job/1018240805/filter-HIV-1%20Subtype%20A%20(A1)) | A1 | AD | [(A1)](http://dbpartners.stanford.edu:8080/RegaSubtyping/stanford-hiv/typingtool/job/1018240805/filter-HIV-1%20Subtype%20A%20(A1))  Check for 35-AD | [(A1)](http://dbpartners.stanford.edu:8080/RegaSubtyping/stanford-hiv/typingtool/job/1018240805/filter-HIV-1%20Subtype%20A%20(A1)) | A1 (6), 35-AD (2) |
| **72** | A | NA | [(A1)](http://dbpartners.stanford.edu:8080/RegaSubtyping/stanford-hiv/typingtool/job/1018240805/filter-HIV-1%20Subtype%20A%20(A1)) | [(A1)](http://dbpartners.stanford.edu:8080/RegaSubtyping/stanford-hiv/typingtool/job/1018240805/filter-HIV-1%20Subtype%20A%20(A1)) | A1 | AD | [(A1)](http://dbpartners.stanford.edu:8080/RegaSubtyping/stanford-hiv/typingtool/job/1018240805/filter-HIV-1%20Subtype%20A%20(A1))  Check for 35-AD | [(A1)](http://dbpartners.stanford.edu:8080/RegaSubtyping/stanford-hiv/typingtool/job/1018240805/filter-HIV-1%20Subtype%20A%20(A1)) | A1 (5), A (1), NA (1), CRF69-01B (1), BF (1) |
| **73** | A | [(A1)](http://dbpartners.stanford.edu:8080/RegaSubtyping/stanford-hiv/typingtool/job/1018240805/filter-HIV-1%20Subtype%20A%20(A1)) | [(A1)](http://dbpartners.stanford.edu:8080/RegaSubtyping/stanford-hiv/typingtool/job/1018240805/filter-HIV-1%20Subtype%20A%20(A1)) | [(A1)](http://dbpartners.stanford.edu:8080/RegaSubtyping/stanford-hiv/typingtool/job/1018240805/filter-HIV-1%20Subtype%20A%20(A1)) | A1 | AD | [(A1)](http://dbpartners.stanford.edu:8080/RegaSubtyping/stanford-hiv/typingtool/job/1018240805/filter-HIV-1%20Subtype%20A%20(A1))  Check for 35-AD | [(A1)](http://dbpartners.stanford.edu:8080/RegaSubtyping/stanford-hiv/typingtool/job/1018240805/filter-HIV-1%20Subtype%20A%20(A1)) | A1 (6), 35-AD (1), A (1) |
| **74** | 35_AD | NA | [(A1)](http://dbpartners.stanford.edu:8080/RegaSubtyping/stanford-hiv/typingtool/job/1018240805/filter-HIV-1%20Subtype%20A%20(A1)) | [(A1)](http://dbpartners.stanford.edu:8080/RegaSubtyping/stanford-hiv/typingtool/job/1018240805/filter-HIV-1%20Subtype%20A%20(A1)) | A1 | AD | [(A1)](http://dbpartners.stanford.edu:8080/RegaSubtyping/stanford-hiv/typingtool/job/1018240805/filter-HIV-1%20Subtype%20A%20(A1))  Check for 35-AD | [(A1)](http://dbpartners.stanford.edu:8080/RegaSubtyping/stanford-hiv/typingtool/job/1018240805/filter-HIV-1%20Subtype%20A%20(A1)) | A1 (5), 35-AD (2), NA (1) |
| **75** | 35_AD | NA | [(A1)](http://dbpartners.stanford.edu:8080/RegaSubtyping/stanford-hiv/typingtool/job/1018240805/filter-HIV-1%20Subtype%20A%20(A1)) | [(A1)](http://dbpartners.stanford.edu:8080/RegaSubtyping/stanford-hiv/typingtool/job/1018240805/filter-HIV-1%20Subtype%20A%20(A1)) | A1 | AD | [(A1)](http://dbpartners.stanford.edu:8080/RegaSubtyping/stanford-hiv/typingtool/job/1018240805/filter-HIV-1%20Subtype%20A%20(A1))  Check for 35-AD | [(A1)](http://dbpartners.stanford.edu:8080/RegaSubtyping/stanford-hiv/typingtool/job/1018240805/filter-HIV-1%20Subtype%20A%20(A1)) | A1 (5), 35-AD (2), NA (1) |
| **76** | 35_AD | NA | [(A1)](http://dbpartners.stanford.edu:8080/RegaSubtyping/stanford-hiv/typingtool/job/1018240805/filter-HIV-1%20Subtype%20A%20(A1)) | [(A1)](http://dbpartners.stanford.edu:8080/RegaSubtyping/stanford-hiv/typingtool/job/1018240805/filter-HIV-1%20Subtype%20A%20(A1)) | A1 | CRF-01B | [(A1)](http://dbpartners.stanford.edu:8080/RegaSubtyping/stanford-hiv/typingtool/job/1018240805/filter-HIV-1%20Subtype%20A%20(A1))  Check for 35-AD | [(A1)](http://dbpartners.stanford.edu:8080/RegaSubtyping/stanford-hiv/typingtool/job/1018240805/filter-HIV-1%20Subtype%20A%20(A1)) | A1 (5), AD (2), NA (1), CRF69-01B (1), BF (1) |
| **77** | 35_AD | [(A1)](http://dbpartners.stanford.edu:8080/RegaSubtyping/stanford-hiv/typingtool/job/1018240805/filter-HIV-1%20Subtype%20A%20(A1)) | [(A1)](http://dbpartners.stanford.edu:8080/RegaSubtyping/stanford-hiv/typingtool/job/1018240805/filter-HIV-1%20Subtype%20A%20(A1)) | [(A1)](http://dbpartners.stanford.edu:8080/RegaSubtyping/stanford-hiv/typingtool/job/1018240805/filter-HIV-1%20Subtype%20A%20(A1)) | A1 | AD | [(A1)](http://dbpartners.stanford.edu:8080/RegaSubtyping/stanford-hiv/typingtool/job/1018240805/filter-HIV-1%20Subtype%20A%20(A1))  Check for 35-AD | [(A1)](http://dbpartners.stanford.edu:8080/RegaSubtyping/stanford-hiv/typingtool/job/1018240805/filter-HIV-1%20Subtype%20A%20(A1)) | A1 (6), 35-AD (2) |
| **Frequency** | 35-AD (94.9%)  A (5.1%) | A1 (84.6%)  NA (15.4%) | A1 (96.2%)  CRF01 (3.8%) | A1 (96.2%)  F1 (2.6%)  D (1.3%) | A1 (100%) | AD (94.9%)  CRF-01B (2.6%)  BF (2.6%) | [(A1)](http://dbpartners.stanford.edu:8080/RegaSubtyping/stanford-hiv/typingtool/job/1018240805/filter-HIV-1%20Subtype%20A%20(A1))  Check for 35-AD (98.7%)  B (1.3%) | A1 (97.4%)  A1 & B (1.3%)  A1 & K (1.3%) |  |

**Supplemental Table 11:** Comparison of RAMS and NOPs between CRF 35-AD and other subtypes

| **Subtypes** | **Mutations in our study** | **Mutations in other studies** | **References** |
| --- | --- | --- | --- |
| **A** | G163R | G163R | Primary resistance to integrase strand transfer inhibitors in patients infected with diverse HIV-1 subtypes in sub-Saharan Africa |
| **B** | V72I, I201V | V72I, I201V | An HIV-1 integrase genotype assay for the detection of drug resistance mutations |
|  |  |  | Differences among HIV-1 subtypes indrug resistance against integrase inhibitors |
|  | M50I, R263K | M50I+ R263K | Structural Comparison of Diverse HIV-1 Subtypes using Molecular Modelling and Docking Analyses of Integrase Inhibitors |
|  | L74I/M, R263K, S230N | L74I/M, R263K, S230N | Differences among HIV-1 subtypes indrug resistance against integrase inhibitors |
| **M** | L74I | 74IV, 138D, 151I, 157Q and 163AE | A genotypic assay for the amplification and sequencing of integrase from diverse HIV-1 group M subtypes |
| **C** | R263K | R263K/R | Differences among HIV-1 subtypes indrug resistance against integrase inhibitors |
|  | G163R | G163R | Primary resistance to integrase strand transfer inhibitors in patients infected with diverse HIV-1 subtypes in sub-Saharan Africa |
|  | M50I | M50I | Structural Comparison of Diverse HIV-1 Subtypes using Molecular Modelling and Docking Analyses of Integrase Inhibitors |
| **D** | G163R | G163R | Primary resistance to integrase strand transfer inhibitors in patients infected with diverse HIV-1 subtypes in sub-Saharan Africa |
| **G** | L74I | L74I | High prevalence of integrase mutation L74I in West African HIV-1 subtypes prior to integrase inhibitor treatment |
| **Non B** | L101, T124A, V72I, L74I, S119P | L101I, T124A, V72I, L74I, S119P, | Differences among HIV-1 subtypes indrug resistance against integrase inhibitors |
| **M,O,N,P** | S230N, L74M, G163R | S230N, L74M in CRF43-02G  G163R/K in CRF-BF | Differences among HIV-1 subtypes indrug resistance against integrase inhibitors |
| **Recombinant**  **(AD,CRF-02AG, AG, DG, CD )** | Q95K  L74M | Q95K  L74M | Primary resistance to integrase strand transfer inhibitors in patients infected with diverse HIV-1 subtypes in sub-Saharan Africa First Report of Drug Resistance against HIV-1 Integrase Inhibitors in Iran |
| **A, C, D, RF** | R263K | R263K | Circulating HIV‐1 integrase genotypes in Tanzania: Implication on the introduction of Integrase Inhibitors‐based ART regimen |
| **All subtypes** | L74I/M | L74I/M | Primary resistance to integrase strand transfer inhibitors in patients infected with diverse HIV-1 subtypes in sub-Saharan Africa |
