# Supplementary material for: First report of computational protein–ligand docking to evaluate susceptibility to HIV integrase inhibitors in HIV-infected Iranian patients
Source: Biochem Biophys Rep. 2022 Mar 29;30:101254. doi: 10.1016/j.bbrep.2022.101254 (PMC8968007; doi:10.1016/j.bbrep.2022.101254)

**Supplemental figure 1:** Docking analysis between reference protein and CAB


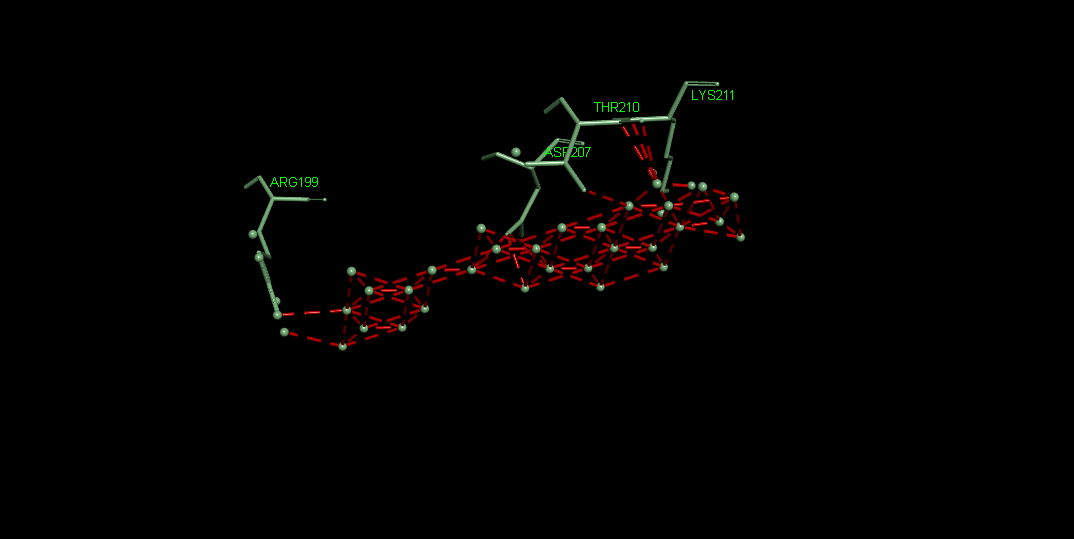


**Supplemental figure 2:** Docking analysis between reference protein and BIC
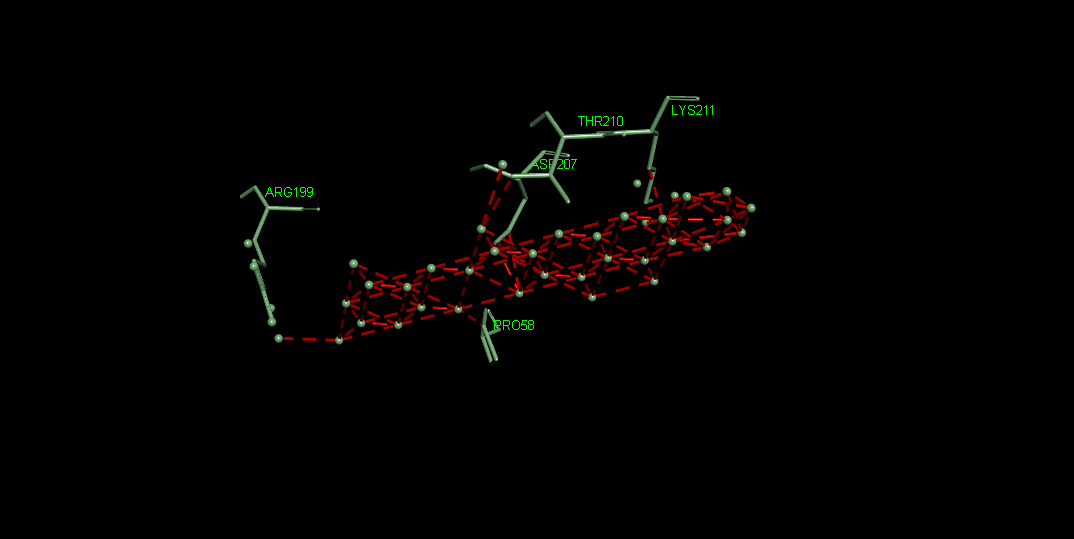


**Supplemental figure 3:** Docking analysis between reference protein and DTG
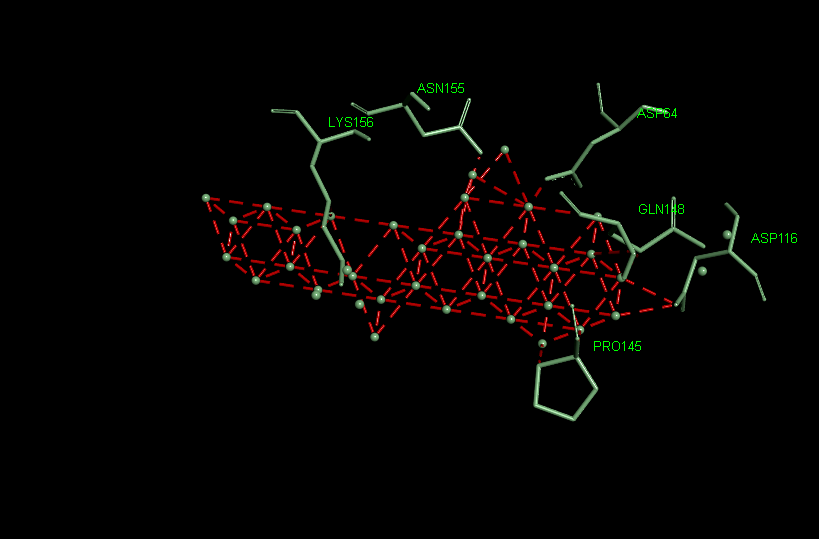


**Supplemental figure 4:** Docking analysis between reference protein and EVG


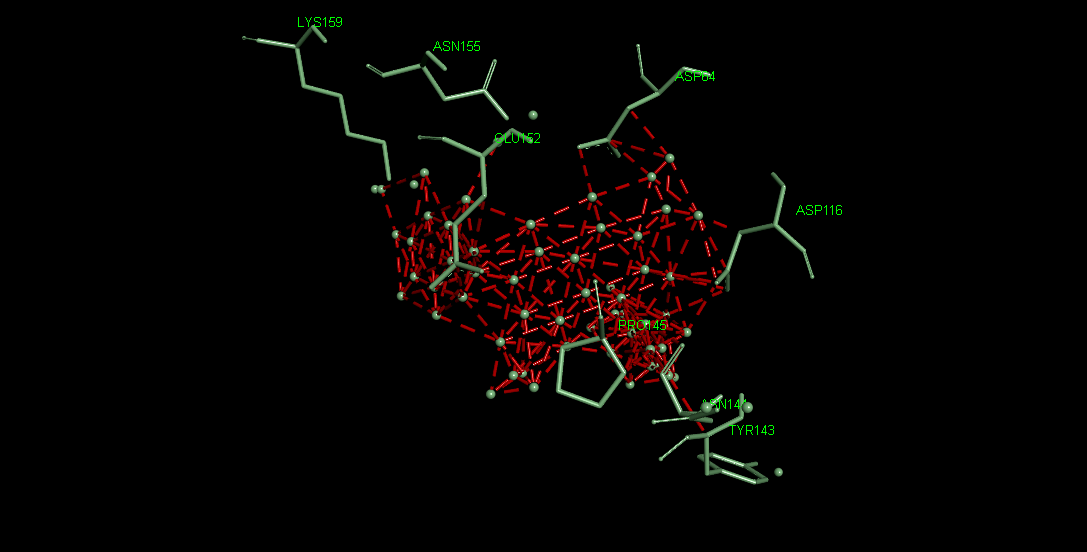


**Supplemental figure 5:** Docking analysis between reference protein and RAL


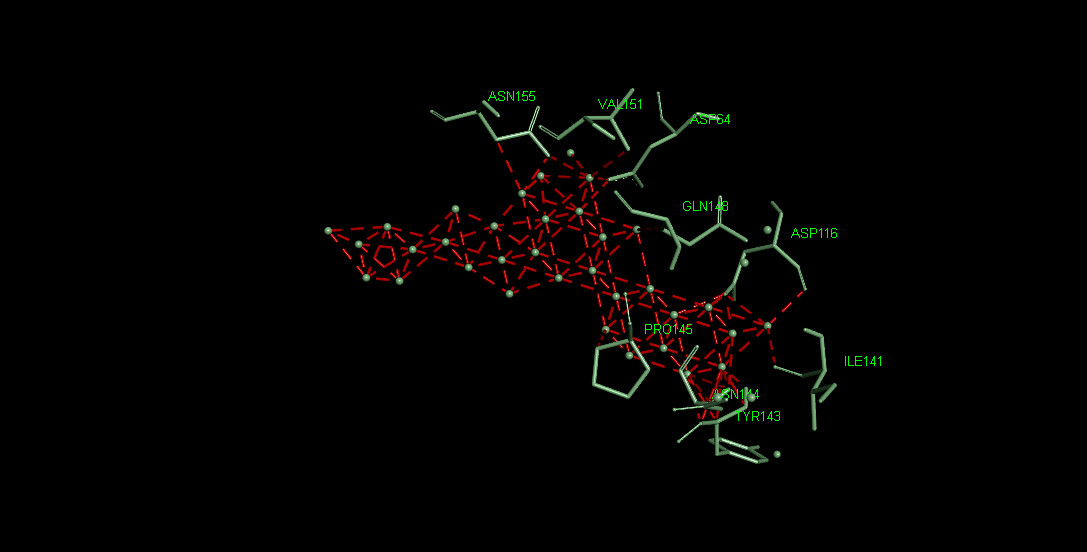

Supplement: Multimedia component 2 [file mmc2.docx]
